# Histone Deacetylase Inhibitor Alleviates the Neurodegenerative Phenotypes and Histone Dysregulation in Presenilins-Deficient Mice

**DOI:** 10.3389/fnagi.2018.00137

**Published:** 2018-05-15

**Authors:** Ting Cao, Xiaojuan Zhou, Xianjie Zheng, Yue Cui, Joe Z. Tsien, Chunxia Li, Huimin Wang

**Affiliations:** ^1^Shanghai Key Laboratory of Brain Functional Genomics, Key Laboratory of Brain Functional Genomics, Ministry of Education, School of Psychology and Cognitive Science, East China Normal University Shanghai, China; ^2^Brain and Behavior Discovery Institute and Department of Neurology, Medical College of Georgia at Augusta University Augusta, GA, United States; ^3^NYU-ECNU Institute of Brain and Cognitive Science, New York University Shanghai Shanghai, China; ^4^Shanghai Changning-ECNU Mental Health Center Shanghai, China

**Keywords:** Alzheimer’s disease, histone deacetylase inhibitor, fear memory, tau hyperphosphorylation, neuroinflammation, histone acetylation dysregulation

## Abstract

Histone acetylation has been shown to play a crucial role in memory formation, and histone deacetylase (HDAC) inhibitor sodium butyrate (NaB) has been demonstrated to improve memory performance and rescue the neurodegeneration of several Alzheimer’s Disease (AD) mouse models. The forebrain presenilin-1 and presenilin-2 conditional double knockout (cDKO) mice showed memory impairment, forebrain degeneration, tau hyperphosphorylation and inflammation that closely mimics AD-like phenotypes. In this article, we have investigated the effects of systemic administration of NaB on neurodegenerative phenotypes in cDKO mice. We found that chronic NaB treatment significantly restored contextual memory but did not alter cued memory in cDKO mice while such an effect was not permanent after treatment withdrawal. We further revealed that NaB treatment did not rescue reduced synaptic numbers and cortical shrinkage in cDKO mice, but significantly increased the neurogenesis in subgranular zone of dentate gyrus (DG). We also observed that tau hyperphosphorylation and inflammation related protein glial fibrillary acidic protein (GFAP) level were decreased in cDKO mice by NaB. Furthermore, GO and pathway analysis for the RNA-Seq data demonstrated that NaB treatment induced enrichment of transcripts associated with inflammation/immune processes and cytokine-cytokine receptor interactions. RT-PCR confirmed that NaB treatment inhibited the expression of inflammation related genes such as S100a9 and Ccl4 found upregulated in the brain of cDKO mice. Surprisingly, the level of brain histone acetylation in cDKO mice was dramatically increased and was decreased by the administration of NaB, which may reflect dysregulation of histone acetylation underlying memory impairment in cDKO mice. These results shed some lights on the possible molecular mechanisms of HDAC inhibitor in alleviating the neurodegenerative phenotypes of cDKO mice and provide a promising target for treating AD.

## Introduction

Alzheimer’s disease (AD) is one of the most common neurodegenerative disorders characterized by progressive cognitive decline and memory impairment. It is defined by the presence of neurofibrillary tangles (NFTs) and senile plaques in the brain, composed of hyperphosphorylated tau and extracellular insoluble β-Amyloid (Aβ), respectively (Selkoe, 2001). Presenilins are the core catalytic components of γ-secretase involved in the processing of amyloid precursor protein (APP). The malfunction of Presenilins ultimately causes amyloid to accumulate into plaques in the brain (De Strooper et al., 1998). Mutants in Presenilin 1 (PS1) or Presenilin 2 (PS2) genes have been identified as the major cause of familial AD (FAD; Levy-Lahad et al., 1995; Sherrington et al., 1995; Hardy, 1997). PS1 and PS2 conditional double knockout (cDKO) mice showed severe AD-like neurodegenerative phenotypes, including forebrain degeneration (e.g., cortical shrinkage), synaptic dysfunction, tau hyperphosphorylation and age-dependent memory decline (Feng et al., 2004; Saura et al., 2004), also exhibited significantly increased inflammatory responses (Beglopoulos et al., 2004; Jiang et al., 2009). But it is worth noting that memory deficits and neurodegeneration in cDKO mice are not caused by Aβ accumulation as PS deletion reduced Aβ production (Beglopoulos et al., 2004), which is inconsistent with the Aβ hypothesis of the pathogenesis of AD. Therefore, cDKO mice could serve as a particularly useful model to study the molecular mechanisms of memory deficits and neurodegenerative phenotype in Aβ-independent AD.

Histone acetylation, which is regulated by histone acetyltransferases (HATs) and histone deacetylases (HDACs), is one of the epigenetic modifications and plays a critical role in chromatin remodeling and transcriptional regulation of gene expression. Recently, accumulating studies have suggested that histone acetylation is involved in memory consolidation and may contribute to cognitive decline associated with neurodegenerative diseases. An increased acetylation of histone H3, H2B and H4 was found in hippocampus after learning processes (Levenson et al., 2004; Bousiges et al., 2010; Peleg et al., 2010), suggesting that histone acetylation may be essential for memory formation. Alterations of histone acetylation were also observed in AD mouse models and AD patients. Acetylation of hippocampal H4 was significantly reduced after fear conditioning training in APP/PS1 mouse models of familial AD (Francis et al., 2009). Furthermore, it has been demonstrated that lower histone H3 K18/K23 acetylation (Zhang et al., 2012), correlating with an increased HDAC2 and HDAC6 expression levels in post-mortem AD brain (Ding et al., 2008; Gräff et al., 2012). On the other hand, increased histone acetylation was also found in AD brains (Narayan et al., 2015).

Much evidence indicates that administration of HDAC inhibitors was capable of not only facilitating memory formation in normal mice (Levenson et al., 2004), but also reversing the histone acetylation abnormality and improving memory in aged mice (Peleg et al., 2010) and neurodegenerative mice (Mastroeni et al., 2011; Lu et al., 2015). HDAC inhibitors exerted a potential effect in attenuating memory deficits (Fischer et al., 2007; Francis et al., 2009; Gräff et al., 2012; Rumbaugh et al., 2015), improving dendrite sprouting and dendritic spine densities (Fischer et al., 2007; Ricobaraza et al., 2012; Song et al., 2016), increasing number of synapses and activating the transcription of synaptic plasticity markers (Fischer et al., 2007; Ricobaraza et al., 2012), decreasing tau hyperphosphorylation (Ricobaraza et al., 2009; Sung et al., 2013), and reducing neuroinflammation (Zhang and Schluesener, 2013), while having inconsistent effects on Aβ levels in different AD mouse models (Ricobaraza et al., 2009; Sung et al., 2013; Yang et al., 2014). In this study, we use a HDAC inhibitor NaB to test whether pharmacologic regulation of histone acetylation improves memory impairment and neurodegenerative phenotypes in cDKO mice, an Aβ-independent mouse model of AD.

## Materials and Methods

### Animals and Drugs

cDKO mice on B6CBA background were generated as described previously (Feng et al., 2004). Male mice were used in the experiments and age-matched mice with the same genetic background were served as wild-type (WT) control. All mice were housed at 20–26°C and in 40%–70% humidity under 12 h/12 h light/dark cycles. All the experiments were approved by the Animal Ethics Committee of East China Normal University.

Sodium butyrate (NaB) was purchased from Sigma (St. Louis, MO, USA) and freshly prepared before administration. NaB (dissolved in 0.9% saline) was administered to cDKO and WT mice via intraperitoneal (i.p.) injection in a volume of 100 ul/10 g of body weight at 1.2 g/kg dose once a day for continuous 21 days. Saline (Vehicle, Veh) was administered to cDKO and WT mice using the same procedure.

### Fear Conditioning

Before fear conditioning training, mice were individually habituated to the Med Associated fear-conditioning chambers for 2 days. During training sessions, mice were placed in the chamber for 3 min, followed by a 30 s tone (85 dB sound at 2800 Hz) with 2 s of 0.75 mA foot shock in the end, were then left in the chamber for additional 30 s. During the retention test, 24 h or 1 month after training, each mouse was first placed in the conditioned chamber for 3 min and the freezing time was measured (contextual memory test). Subsequently, the mice were placed into a novel chamber and stayed for 3 min. Then, a tone identical to the training session was delivered for 3 min and the time spent freezing was recorded (cued memory test).

### Open Field

The locomotor activity of mice was examined in a 27 × 27 × 38 cm open field chamber. After 3 weeks of NaB treatment, 1 h after the last injection, mice were placed in the open field arena for 30 min. The travel trace of mouse was measured and analyzed by Truscan Open Field software. Locomotor activity was evaluated as total distance traveled. Anxiety-like behavior was defined by time spent in the center area, which is 8.76 cm away from the open field chamber wall.

### Western Blotting

Six mice of NaB- and Veh-treated groups were used for western blotting. Mice were killed by cervical dislocation and the cortex and the hippocampus were quickly dissected. Histone protein and total protein were prepared from both tissues as previously described, respectively (Shechter et al., 2007; Li et al., 2011). Purified proteins were separated on SDS-PAGE and quantified using Odyssey LI-COR. GAPDH (1:5000, Bioworld, St. Louis Park, MN, USA) was used as loading control. Commercial primary antibodies used include Acetylated Histone 3 (Ac-H3; 1:2000, Upstate Biotechnology, Merck Millipore, Darmstadt, Germany), Histone 4 (1:1000, Bioworld, St. Louis Park, MN, USA), Tau-P (phospho S199 + S202; 1:1000, Abcam, Cambridge, UK), Caspase 3 (1:500, Bioworld, St. Louis Park, MN, USA), cleaved caspase 3(1:100, chemicon, Merck Millipore, Darmstadt, Germany), Bcl-2 (1:500, Bioworld, St. Louis Park, MN, USA) and glial fibrillary acidic protein (GFAP; 1:500, ZSGB-Bio, Beijing, China). Secondary antibodies used included anti-Mouse IRDye 800CW and anti-Rabbit IRDye 800CW (1:5000, Li-Cor Biosciences, Cambridge, UK).

### Histology

Three mice of NaB- and Veh-treated groups were used for histological analysis. Five-month-old mice were deeply anesthetized and perfused with ice-cold 1× PBS and 4% paraformaldehyde. Brains were then fixed overnight at 4°C, then immersed into 20% and 30% sucrose-PBS for dehydration. Coronal sections of 20 μm were cut using a cryostat (Leica Microsystems, Nussloch, Germany). The brain sections were used for Hematoxylin-Eosin (HE) staining. After staining, they were imaged under Leica DM400B fluorescent microscope (Leica Microsystems, Nussloch, Germany). The Leica Qwin software was used to measure the thicknesses of the cortex of the sections.

### Electron Microscopic Analysis

Five mice of NaB- and Veh-treated groups were used for electron microscopic analysis. Mice were anesthetized and perfused with ice-cold 0.9% saline followed by the fixative with 2.5% glutaraldehyde in 0.1 M phosphate buffer (pH 7.4). Brains were removed and post-fixed in the same fixative overnight. For electron microscopic analysis, the hippocampal CA1 stratum radiatum was cut and collected in the fixative for 1–2 h, and then washed for 15 min for three times in 0.1 M phosphate buffer and fixed in 1% osmium tetroxide for 1 h. After rinsing three times, 15 min each in phosphate buffer, the tissues were dehydrated in 30% ethanol, 50% ethanol, 70% ethanol, 90% ethanol, 90% ethanol + 90% acetone and 90% acetone for 15 min each and subsequently infiltrated and flat embedded with 100% acetone + Embed 812 resin (1:1) for 3 h, and Embed 812 for 2 h. They were polymerized for 18 h at 35°C, 24 h at 45°C, and 48 h at 60°C, and after that they were naturally cooled to room temperature. Ultrathin sections (70 nm) were cut and stained with 1% uranyl acetate and lead citrate. Thirty electron micrographs taken at low magnification (4000×) on a JEM-2100 Transmission electron microscope (JEOL, Akishima, Tokyo) for each mouse were used for counting synapses (excitatory synapses). Synaptic ultrastructure was verified by the images taken at high magnification (8000×). Synaptic density was analyzed as described previously (Rampon et al., 2000).

### Neurogenesis

Two days before the end of the treatment period, the Veh and NaB-treated cDKO mice were given BrdU (10 mg/ml freshly prepared in sterile 0.9% NaCl, Sigma, St. Louis, MO, USA) at a dosage of 100 mg/kg body weight. All mice were i.p. administrated BrdU for four times with a 2 h interval daily for 2 days. Eighteen hours after the final BrdU injection, mice were anaesthetized and perfused with 0.9% NaCl solution followed by 4% PFA for 2 h, and then immersed in 20% and 30% sucrose. Subsequently, 30 μm coronal sections from each mouse were cut from Bregma-1.34 to Bregma-2.46 by cryostat (Leica Microsystems, Nussloch, Germany). They were washed with 1× PBS with 0.1 Triton X-100 for 3 × 5 min and incubated in an antigen retrieval solution for 15 min (10 mM sodium citrate buffer, pH 6.0, at 99°C). After cooling, the sections were rinsed with PBS for 5 min, and treated with a blocking solution (5% goat serum, 3% Triton, 3% BSA in 0.1 M PBS) for 1 h at room temperature. The sections were then incubated overnight at 4°C in a primary antibody solution: mouse anti-BrdU (1:5000, Merck Millipore, Billerica, MA, USA), and rabbit anti-DCX (1:200, Abcam, Cambridge, MA, USA). After 8 × 5 min wash in 1× PBS with 0.1% Tween-20, the sections were incubated for 2 h at room temperature in a second antibody solution: goat anti mouse cy3 (1:500, Merck Millipore, Billerica, MA, USA), and goat anti-rabbit Alexa 488 (1:1000, Invitrogen, Waltham, MA, USA). The sections were once again washed 6 × 5 min in 1× PBS with 0.1% Tween-20 before, and mounted onto glass slides with Fluoro-Gel. Images were taken with Zeiss Axio Imager A1 microscope (Carl Zeiss Microscopy GmbH, Oberkochen, Germany). The number of BrdU-positive (BrdU^+^) cells and BrdU/DCX co-labeled (BrdU^+^/DCX^+^ cells) in dentate gyrus (DG) from each section was multiplied by 15 (there were 15 sections from each mouse) to estimate the total numbers of BrdU^+^ cells and BrdU^+^/DCX^+^ cells throughout the DG per mouse.

### RNA-Seq

Forebrains of cDKO mice treated with Veh or NaB for 3 weeks, three mice per group, were dissected and pooled. Total RNA was extracted using TRIzol Reagent (Life Technologies, Carlsbad, CA, USA) following the manufacturer’s instructions. RNA quality was confirmed using NanoDrop ND-1000 (Thermo Scientific, Waltham, MA, USA) and Agilent Bioanalyzer 2100 (Agilent Technologies, Santa Clara, CA, USA). Sequencing libraries were prepared using TruSeq RNA Sample Preparation Kit (Illumina, San Diego, CA, USA) and sequenced on Illumina HiSeq 2000 system (Illumina, San Diego, CA, USA). After filtering and removing of low-quality reads, clean reads were obtained and mapped to the UCSC mouse genome mm10 using Tophat (version 2.0.9). Mapped reads were then assembled by the Cufflinks program (version 2.2.1) and FPKM (Fragments Per Kilobase of exon model per Million mapped reads) was calculated to assess gene expression. Differential expression analysis was performed by DESeq. Comparisons were made between NaB-treated cDKO mice and Veh-treated cDKO mice. Genes with expression fold changes ≥2 were selected for further analysis. Differentially expressed genes were submitted to the DAVID website (DAVID 6.8) for GO classification analysis according to biological process and Kyoto Encyclopedia of Genes and Genomes (KEGG) pathway enrichment. GO and Pathways were considered significantly enriched with *p* < 0.05 and FDR < 0.05.

### Real Time PCR

Real time quantitative PCR (qPCR) was performed as described previously (Li et al., 2011). Briefly, forebrain of cDKO mice treated with Veh or NaB for 3 weeks was dissected and immediately frozen in liquid nitrogen. The brains were stored in a freezer at −70°C prior to use. Total RNA was extracted from frozen forebrain using Trizol (Invitrogen, USA) and cDNA was generated using moloney murine leukemia virus (M-MLV) reverse trancriptase (Invitrogen, USA). Diluted cDNA was used as a template for the SYBR Green qPCR analysis (The primer sequences are listed in Supplementary Material, Supplementary Table S1). The experiment was performed using CFX97 real time system (Bio-Rad, USA). Glyceraldehyde-3-phosphate dehydrogenase (GAPDH) was used as the reference gene. The gene expression levels were calculated and described as 2^−ΔΔCt^ values.

### Statistical Analysis

All data are presented as mean ± SEM. Two-way ANOVA followed by Tukey’s *post hoc* test was used to compare the effect of genotype and NaB treatment on contextual and cued memory, synaptic density, thickness of cortex, neurogenesis, protein levels of phosphorylation of Tau and GFAP, and levels of acetylation of histone 3. Statistical significance was set at *p* < 0.05.

## Results

### Impaired Fear Associative Memory in cDKO Mice

**Figure 1:**
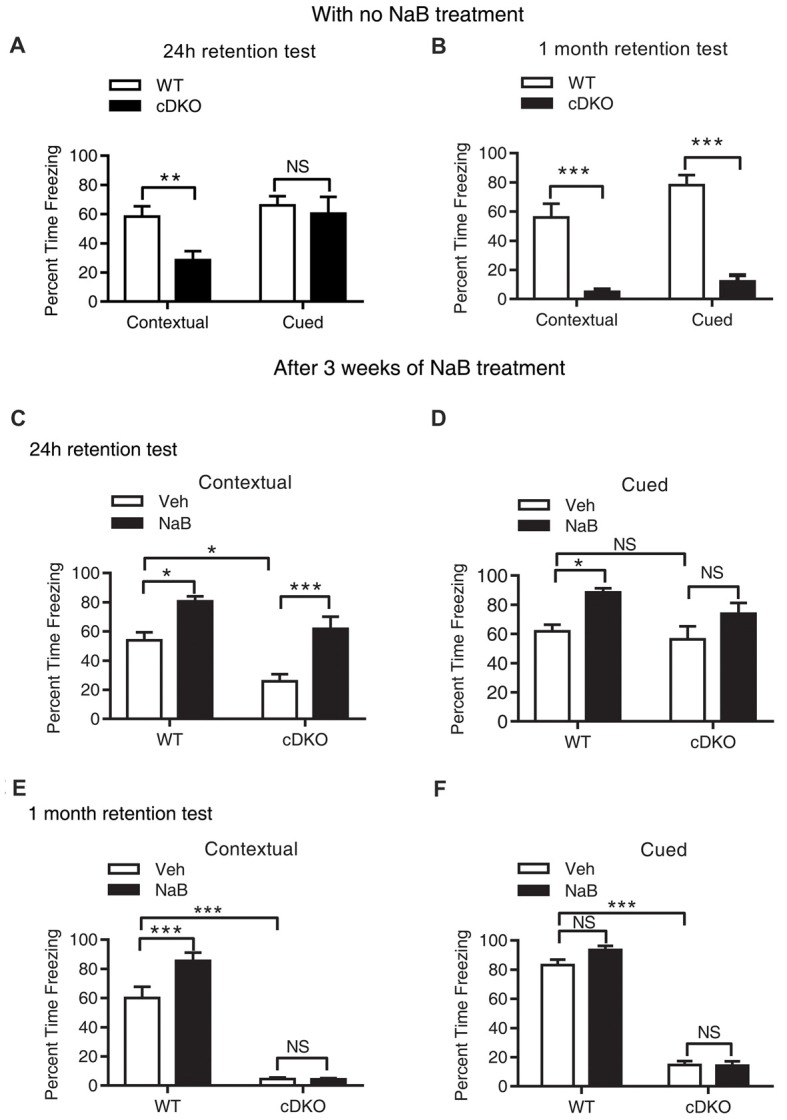
Long-term continuous treatment with sodium butyrate (NaB) reversed contextual memory deficits in presenilin conditional double knockout (cDKO) mice. **(A,B)** Wild-type (WT) and cDKO mice (5-month-old) were trained in a fear conditioning paradigm, 24 h **(A)** and 1 month **(B)** after training (24 h retention test: WT *n* = 8, cDKO *n* = 8; 1 month retention test: WT *n* = 8, cDKO *n* = 10), all mice were returned to the conditioning chamber and new chamber for contextual and cued memory test, respectively. Percent time freezing was measured for each animal. Compared with WT mice, cDKO mice showed contextual memory impairment in 24 h and 1 month retention test but exhibited cued memory deficits only in 1 month retention test. **(C–F)** WT and cDKO mice were injected with either vehicle (Veh) or 1.2 g/kg NaB daily for 3 weeks, 1 h after the last injection, all mice were then trained in a fear conditioning paradigm and tested for memory 24 h (**C,D**: Veh: WT *n* = 12, cDKO *n* = 11; NaB: WT *n* = 12, cDKO *n* = 13) or 1 month (**E,F**: Veh: WT *n* = 11, cDKO *n* = 18; NaB: WT *n* = 15, cDKO *n* = 15) later. Three weeks continuous treatment with NaB rescued contextual memory **(C)** and unchanged cued memory **(D)** in cDKO mice. One month after treatment withdrawal, cDKO mice with previous NaB treatment behaved similarly as Veh-treated cDKO mice during the contextual memory **(E)** and cued memory **(F)** test. Data was expressed as mean ± SEM. Two tailed student *t*-test for **(A,B)**, Two-way ANOVA with Tukey’s *post hoc* tests for **(C–F)**. NS, Not significant, *P < 0.05, **P < 0.01, ***P < 0.001.

Mild and severe impairments of associative memory were observed in presenilins cDKO mice at 2 months and 6 months, respectively (Saura et al., 2004). Using fear conditioning task, we found that 5-month-old cDKO mice exhibited contextual memory deficit in both 24-h and 1-month retention tests (*t* = 3.201, *df* = 14, *p* < 0.01 and *t* = 5.915, *df* = 16, *p* < 0.001, respectively; Figures 1A,B). However, cued memory impairment was only detected in the 1-month retention test (*t* = 8.564, *df* = 16, *p* < 0.001, Figure 1B), but not in the 24-h retention test (Figure 1A, *t* = 0.4252, *df* = 14, *p* = 0.1427).

### Long-Term Continuous Treatment With HDAC Inhibitor NaB Ameliorated Contextual Memory Deficits, Which Is Not Long-Lasting After Treatment Withdrawal in cDKO Mice

To investigate the effect of NaB on memory, we first tested whether NaB treatment could ameliorate memory deficits in cDKO mice. To this end, cDKO and WT mice were injected with NaB or vehicle daily for 3 weeks. One hour after the last injection, 5-month-old mice were trained in fear conditioning paradigm and 24 h later tested for contextual memory and cued memory, respectively.

During the 24-h retention test, two-way ANOVA showed that there was a significant effect of treatment (*F*_(1,44)_ = 27.2, *p* < 0.001) and genotype (*F*_(1,44)_ = 15.31, *p* < 0.001), but there was no effect of treatment × genotype interaction (*F*_(1,44)_ = 0.6097, *p* = 0.4391) in terms of the percentage (%) of time for freezing in contextual memory test (Figure 1C). On the other hand, for cued fear memory, there was a significant effect of treatment (*F*_(1,44)_ = 12.32, *p* < 0.01), but no effect of genotype (*F*_(1,44)_ = 2.514, *p* = 0.12), and treatment × genotype interaction (*F*_(1,44)_ = 0.5099, *p* = 0.4749; Figure 1D). These results indicate that chronic injection of HDAC inhibitor NaB significantly mitigated contextual memory deficits in cDKO mice. To examine whether NaB specifically improves hippocampus dependent memory, we also assessed hippocampus-dependent spatial memory in all four groups using water maze task. After 4 days training, on day 5, we did the probe test and found that NaB did not change the escape latency during training days, but significantly improved target quadrant time of cDKO mice in probe test (data was shown in Supplementary Figure S1). Consistent with our fear conditioning results, the water maze results also indicated that NaB did have beneficial effects on hippocampus dependent memory.

Further, we have investigated whether long term continuous treatment with NaB could have a long-lasting effect after treatment withdrawal. After a treatment with NaB for 3 weeks, 1 h after the last injection, 5-month-old mice were trained in fear conditioning paradigm and tested 1 month later.

In the 1-month memory retention test, cDKO mice had a dramatic loss of contextual and cued fear memory as shown by significant effect of genotype for both types of behavioral tests (contextual: *F*_(1,55)_ = 266.3, *p* < 0.001; cued: *F*_(1,51)_ = 580.6, *p* < 0.001). NaB could still enhance the contextual memory in WT mice, while having no impact on the memory deficits in cDKO mice (Figure 1E; treatment (*F*_(1,55)_ = 9.045, *p* < 0.01); treatment × genotype interaction (*F*_(1,55)_ = 9.245, *p* < 0.01). In the cued memory test, NaB could not improve the memory in both genotypes (Figure 1F) as shown by no effect of treatment (*F*_(1,51)_ = 2.658, *p* = 0.1092) and treatment × genotype interaction (*F*_(1,51)_ = 3.192, *p* = 0.08).

We next assessed whether locomotion activity or anxiety-like behavior was changed by NaB treatment using the open field test. We found that NaB-treated cDKO mice and WT mice did not exhibit significant differences in total distance traveled and time spent in the center compared with Veh-treated cDKO mice and WT mice, respectively (Supplementary Figure S2), suggesting that NaB treatment had no effect on locomotor activity and anxiety-like behavior in both cDKO mice and WT mice. The above results indicate that NaB may rescue the memory impairment, preferentially contextual fear memory, of cDKO mice; however, the beneficial effect of NaB could not last for 1 month.

### NaB Treatment Had No Rescue Effect on the Reduction in Synapse Numbers and Cortical Shrinkage, But Enhanced the Differentiation of Newborn Neurons in the Cortex of cDKO Mice

Because cDKO mice has forebrain degeneration phenotype especially cortical shrinkage, which is also accompanied with severe progressive neuron loss (Feng et al., 2004). In this regard, we examined whether NaB treatment affects the synapse number, cortical thickness and neurogenesis of cDKO mice.

**Figure 2:**
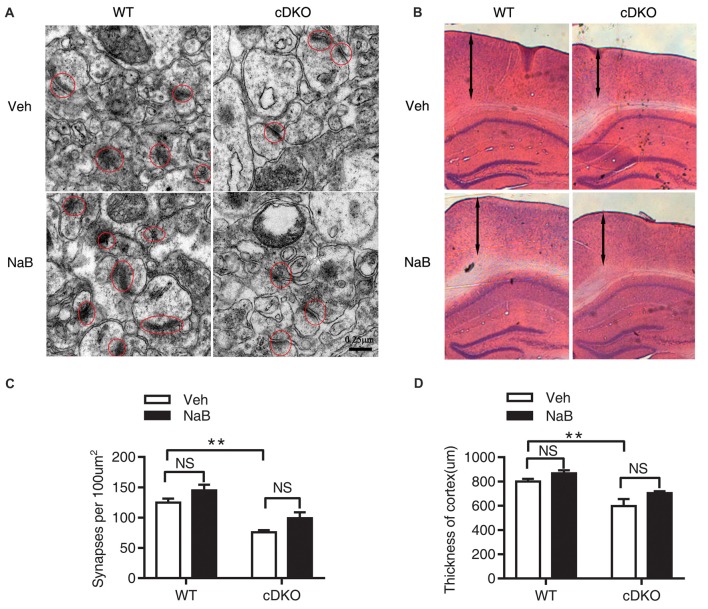
No rescue effects of NaB treatment on the reduction in synapse numbers and cortical shrinkage in the cortex of cDKO mice. Electron microscopic analysis **(A,C)** and HE staining **(B,D)** examining the synapses (red cycle) and cortical thickness (double-headed arrow) in the cortex of cDKO and WT mice after 3 weeks NaB treatment. **(A,C)** The synapse numbers in the cortex were significantly reduced in cDKO+veh mice when compared with WT+veh mice but NaB treatment did not change the synapse numbers in cDKO mice. *n* = 30 from 5 WT mice and *n* = 30 from 5 cDKO mice, respectively for **(A,C)**. **(B)** HE staining of brain slices in cDKO and WT mice with NaB or Vehicle treatment. **(D)** Quantification of the thickness of cortex. Scale bar is 100 μm. *n* = 15 from 3 WT mice and *n* = 15 from 3 cDKO mice, respectively for **(B,D)**. Data was expressed as mean ± SEM. Two-way ANOVA with Tukey’s *post hoc* tests. NS, not significant, **P < 0.01.

The synaptic number was determined using electron microscopic analysis. Two-way ANOVA revealed a significant effect of genotype (*F*_(1,16)_ = 38.04, *p* < 0.0001), treatment (*F*_(1,16)_ = 7.887, *p* < 0.05), but no effect of treatment × genotype interaction (*F*_(1,16)_ = 0.03465, *p* = 0.8547). Tukey’s *post hoc* analysis showed a significant decrease in synaptic number in Veh-treated cDKO mice compared with Veh-treated WT mice (*p* < 0.01). However, in both cDKO and WT groups, no differences were observed in NaB-treated mice relative to Veh-treated mice (Figures 2A,C).

We next examined the cortical thickness using HE staining. Two-way ANOVA revealed a significant effect of genotype (*F*_(1,32)_ = 22.8, *p* < 0.0001), treatment (*F*_(1,32)_ = 5.113, *p* < 0.05), but no effect of treatment × genotype interaction (*F*_(1,32)_ = 0.03465, *p* = 0.2635). Tukey’s *post hoc* analysis found a significant decrease in cortical thickness in Veh-treated cDKO mice compared with Veh-treated WT mice (*p* < 0.01). In contrast, NaB treatment had no effect on cortical shrinkage in cDKO mice (Figures 2B,D).

**Figure 3:**
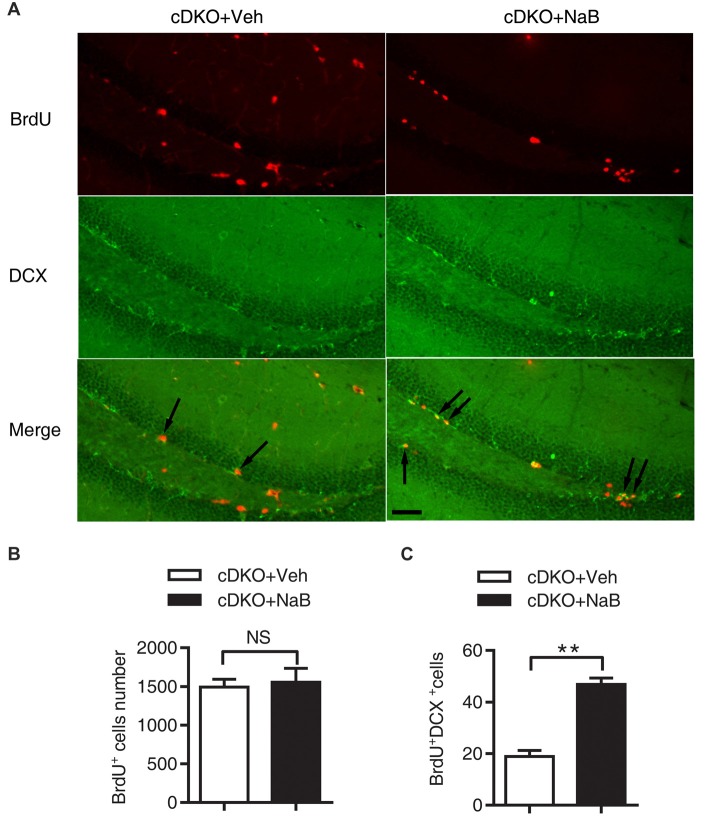
NaB treatment enhanced the differentiation of newborn neurons in dentate gyrus (DG) of cDKO mice. **(A)** BrdU and doublecortin (DCX) double staining (arrow) of coronal sections of brains from NaB or Vehicle treated cDKO mice at the age of 5 months old. **(B,C)** Quantitative analyses of the number of BrdU stained cells **(B)** and BrdU/DCX double stained cells **(C)** in the DG of cDKO mice. The proportion of BrdU^+^/DCX^+^ cells in total BrdU^+^ cells was higher in cDKO mice treated with NaB compared with Veh (*n* = 45 from 3 Vehicle treated cDKO mice and *n* = 45 from 3 NaB treated cDKO mice for **B,C**), but BrdU positive cells did not change after NaB treatment in cDKO mice. Scale bar = 50 μm. Data was expressed as mean ± SEM. Two-way ANOVA with Tukey’s *post hoc* tests. NS, not significant, **P < 0.01.

The impact of NaB treatment on neurogenesis in the cDKO mice was investigated. BrdU immunostaining in the DG revealed no significant difference in the number of BrdU-labeled cells between NaB-treated and Veh-treated cDKO mice (*t* = 0.3078, *df* = 4, *p* = 0.4742, Figures 3A,B). Interestingly, we found a significant increase of BrdU and DCX double stained cell number in NaB-treated cDKO mice compared with Veh-treated mice (*t* = 8.26, *df* = 4, *p* < 0.01, Figures 3A,C). Further, we also examined the effect of NaB on neuron apoptosis by detection of the protein expression levels of cleaved caspase 3 in cortex of cDKO mice and WT mice treated with NaB or vehicle. Surprisingly, the protein levels of cleaved caspase 3 was very low in both 5-month-old WT and cDKO mice and was not changed by NaB treatment (data not shown). Moreover, we also found that NaB treatment did not change the expression level of Bcl-2, a key antiapoptotic regulator, in cDKO mice (data was shown in Supplemental Figure S3). These results suggest that enhanced differentiation of newborn neurons may underlie memory enhancement by NaB treatment in cDKO mice.

### NaB Treatment Reduced Tau Hyperphosphorylation and Astrocyte Activation in cDKO Mice

The previous work has shown that memory deficits in cDKO mice is accompanied with increased tau hyperphosphorylation (Feng et al., 2004; Saura et al., 2004) and astrocyte activation (Beglopoulos et al., 2004; Jiang et al., 2009). We thus examined the effect of NaB treatment on the levels of phosphorylated tau (Ser-199 and Ser-202) and GFAP, a marker of astrocyte activation and inflammatory responses using Western blotting.

**Figure 4:**
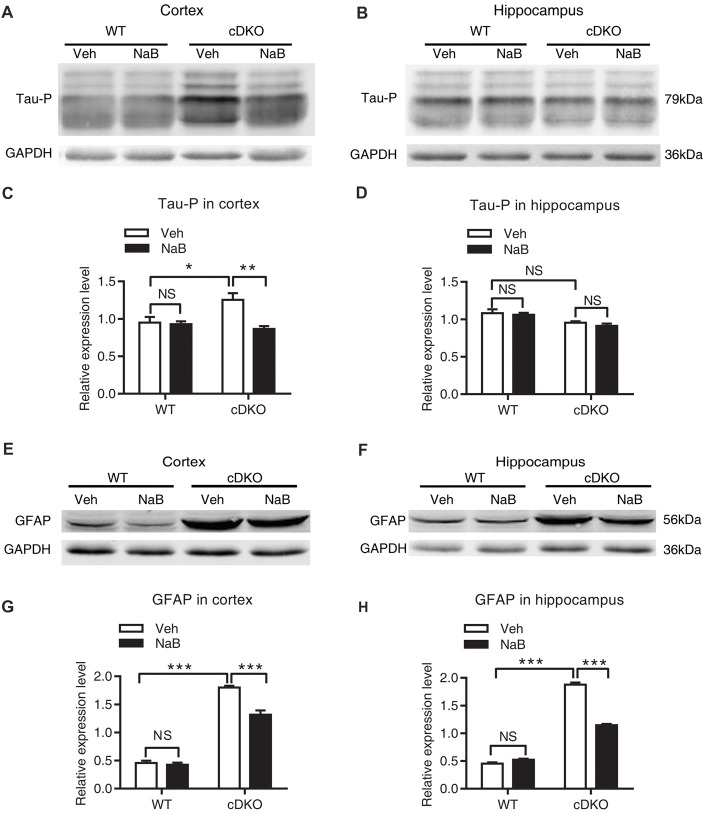
NaB treatment partially suppressed the elevated levels of phosphorylated Tau (Tau-P) and GFAP in cDKO mice. Cortical and hippocampus lysates were analyzed using western blot for phosphorylated Tau protein (Tau-P) **(A,B)** and astrocyte activation marker glial fibrillary acidic protein (GFAP) levels **(E,F)**. **(C,D,G,H)** Quantification of Tau-P and GFAP protein levels in **(A,B)** and **(E,F)**, respectively. All proteins were normalized using GAPDH (six mice per group, *n* = 3). Data was expressed as mean ± SEM. Two-way ANOVA with Tukey’s *post hoc* tests. NS, not significant, *P < 0.05, **P < 0.01, ***P < 0.001.

We found that treatment with NaB decreased tau hyperphosphorylation level only in the cortex of cDKO mice, but not in the hippocampus where the tau hyperphosphorylation phenotype was not observed. In cortex, two-way ANOVA revealed a significant effect of treatment (*F*_(1,12)_ = 10.05, *p* < 0.01), treatment × genotype interaction (*F*_(1,12)_ = 8.09, *p* < 0.05), but not in genotype (*F*_(1,12)_ = 3.43, *p* = 0.0888; Figures 4A,C). Tukey’s *post hoc* analysis showed that tau phosphorylation (Tau-P) was significantly increased in Veh-treated cDKO mice relative to Veh-treated WT mice (*p* < 0.05), which is consistent with the tau hyperphosphorylation observed by previous studies (Saura et al., 2004). NaB significantly decreased the phosphorylated tau (*p* < 0.01) in cDKO mice groups, while having no effect on WT mice. After NaB treatment, the level of tau phosphorylation of cDKO mice showed no difference with WT mice. In hippocampus (Figures 4B,D), two-way ANOVA revealed a significant effect of genotype (*F*_(1,8)_ = 16.53, *p* < 0.01), but not in treatment (*F*_(1,8)_ = 0.8266, *p* = 0.3898), and treatment × genotype interaction (*F*_(1,8)_ = 0.1182, *p* = 0.7398). Tukey’s *post hoc* analysis showed that no differences were observed in Veh-treated cDKO mice compared with Veh-treated WT mice or NaB-treated cDKO mice. WT mice treated with NaB did not change their expression levels of Tau-P as compared with Veh-treated WT mice.

For GFAP, an inflammatory responses marker in the brain, we found that NaB treatment decreased GFAP expression in both cortex and hippocampus in cDKO mice. Two way ANOVA revealed a main effect of genotype (cortex: *F*_(1,16)_ = 512.6, *p* < 0.0001; Hippocampus: *F*_(1,20)_ = 1500, *p* < 0.0001, Figures 4E–H, respectively), treatment (cortex: *F*_(1,16)_ = 27.31, *p* < 0.0001; Hippocampus: *F*_(1,20)_ = 154.4, *p* < 0.0001, Figures 4E–H, respectively), and treatment × genotype interaction (cortex: *F*_(1,16)_ = 21.22, *p* < 0.001; Hippocampus: *F*_(1,20)_ = 229.2, *p* < 0.0001, Figures 4E–H, respectively). In both cortex and hippocampus, Tukey’s *post hoc* analysis showed that GFAP protein levels were significantly increased in Veh-treated cDKO mice relative to Veh-treated WT mice (*p* < 0.0001), which supports the observed inflammation in cDKO mice in the previous studies (Beglopoulos et al., 2004). NaB treatment significantly decreased the expression levels of GFAP in cDKO mice (*p* < 0.0001) but had no effect on WT mice. However, NaB-treated cDKO mice still showed a higher expression levels of GFAP than Veh-treated WT mice (*p* < 0.0001).

### RNA-Seq Revealed Regulation of Immune and Inflammation Response Related Genes Expression by NaB Treatment in cDKO Mice

**Figure 5:**
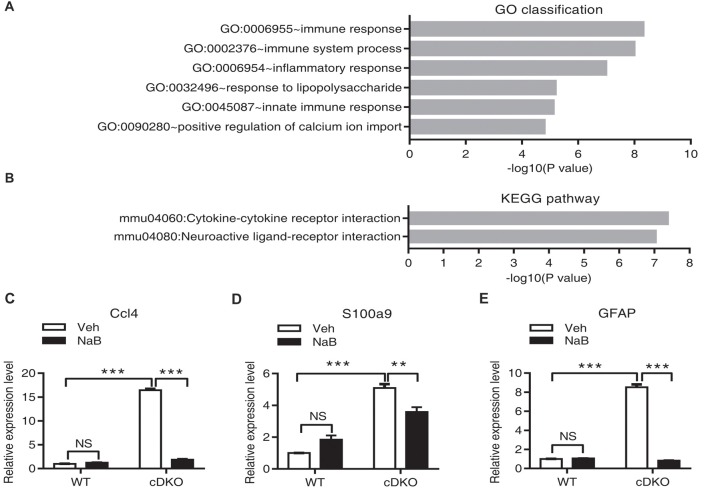
Effect of NaB treatment on the neuroinflammation related genes in cDKO mice. **(A,B)** GO category based on biological process and KEGG pathway enrichment for differentially expressed genes analyzed by deep sequencing in the forebrain of NaB treated cDKO mice compared with Veh treated mice. GO classification **(A)** and pathway enrichment **(B)** were determined using DAVID and a *p* value < 0.05. **(C,E)** The relative mRNA levels of Ccl4 **(C)**, S100a9 **(D)** and GFAP **(E)** were measured using real-time quantitative PCR (qPCR). Relative expression levels were calculated as the ratio of the target gene expression level to the GAPDH expression level in the same sample (*n* = 3 per treatment). Data was expressed as mean ± SEM. Two-way ANOVA with Tukey’s *post hoc* tests. NS, not significant, **P < 0.01, ***P < 0.001.

We performed RNA-Seq analysis to characterize transcriptome differences between NaB-treated cDKO mice and Veh-treated cDKO mice. Total 23,016,260 and 21,836,016 clean reads were generated in NaB-treated cDKO and Veh-treated cDKO mice, respectively. After subsequent removal of some reads with low quantity, 20,082,376 and 19,222,006 were mapped to the UCSC mouse genome mm10, and 87.3% and 88.0% of the transcripts mapped to the mouse genome, respectively. DEGseq analysis was used to determine the differential expressed genes. We found that NaB treatment changed expression of 1707 genes more than two folds in cDKO mice when compared to Veh-treated mice, 617 and 1090 genes were upregulated and downregulated, respectively (Supplementary Table S2). Interestingly, among these genes, inflammatory chemokine (C-C motif) ligand 4 (Ccl4) and inflammatory marker gene S100 calcium binding protein A9 (S100a9), had increased their expression levels in the hippocampus and cortex of 10-month-old cDKO mice when compared to WT mice (Dong et al., 2007), were both downregulated by NaB treatment in the forebrain of cDKO mice. We next performed real-time PCR experiments to confirm the RNA-seq data. Consistent with our previous study (Dong et al., 2007) and RNA-seq data, qPCR results confirmed that the expression levels of Ccl4, S100a9 and GFAP were significantly increased in the cDKO-Veh mice relative to WT-Veh mice and were down-regulated after NaB treatment in cDKO mice, respectively (Figures 5C–E).

To further characterize the function of differential expressed genes in NaB-treated cDKO mice, we used GO enrichment according to biological process which functionally classifies these differential expressed genes into different categories. The main GO classifications were immune response (44 transcripts), immune system process (54 transcripts), inflammatory response (48 transcripts), response to lipopolysaccharide (30 transcripts), innate immune response (48 transcripts), and positive regulation of calcium ion import (9 transcripts) (Figure 5A and Supplementary Table S3). Furthermore, we applied the KEGG database to determine the pathway enrichment for these differential expressed genes, with results shown in Figure 5B and Supplementary Table S4, two pathways were enriched, including cytokine-cytokine receptor interaction (38 transcripts) and neuroactive ligand-receptor interaction (41 transcripts).

### NaB Treatment Reversed Dysregulation of Histone Acetylation in the Cortex and Hippocampus of cDKO Mice

To determine whether the memory deficits observed in cDKO mice are related to the expression level changes of histone acetylation, we assessed the effects of NaB treatment on the histone acetylation. One hour after the last injection, all mice were trained in fear conditioning paradigm. One hour after training, the cortex and hippocampus of the four groups were collected and used for analysis of Ac-H3 levels by Western blotting.

**Figure 6:**
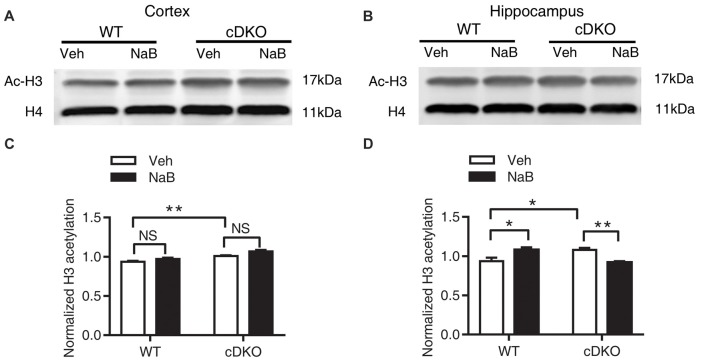
Effect of NaB treatment on expression levels of acetylated histone 3 (Ac-H3) in cDKO mice. **(A,B)** Western blot analysis of Ac-H3 in the cortex **(A)** and hippocampus **(B)**. After 3 weeks of NaB treatment, 1 h after the last injection, mice were trained with fear conditioning paradigm and then killed 1 h later. Purified histone proteins from the cortex and hippocampus were immunoblotted for Ac-H3 and total histone H4 (H4, as loading control). **(C,D)** Quantification of Ac-H3 levels in **(A,B)**, respectively. Normalized H3 acetylation was calculated by dividing the band density of Ac-H3 by the band density of H4 for each sample. Data was expressed as mean ± SEM. Two-way ANOVA with Tukey’s *post hoc* tests. NS, not significant, *P < 0.05, **P < 0.01.

In the cortex, two-way ANOVA revealed a main effect in genotype (*F*_(1,20)_ = 34.32, *p* < 0.0001, Figures 6A,C) and treatment (*F*_(1,20)_ = 9.955, *p* < 0.01, Figures 6A,C), but not in treatment × genotype interaction (*F*_(1,20)_ = 0.6109, *p* = 0.4436, Figures 6A,C). Tukey’s *post hoc* analysis revealed a significant increase in Ac-H3 protein levels in Veh-treated cDKO mice as compared with Veh-treated WT mice (*p* < 0.05). But no changes in Ac-H3 protein levels after NaB treatment were observed in both WT mice and cDKO mice.

In the hippocampus, two-way ANOVA revealed no significant effect in genotype (*F*_(1,14)_ = 0.09047, *p* = 0.7680, Figures 6B,D) and treatment (*F*_(1,14)_ = 0.1526, *p* = 0.7020, Figures 6B,D), but found a significant effect of treatment × genotype interaction (*F*_(1,14)_ = 30.35, *p* < 0.0001, Figures 6B,D). Tukey’s *post hoc* analysis showed a significant increase in Ac-H3 protein levels in Veh-treated cDKO mice when compared with Veh-treated WT mice (*p* < 0.05). Of interest, NaB treatment had a differential effect on WT mice and cDKO mice: it increased Ac-H3 protein levels in WT mice (*p* < 0.05), and in contrast, decreased the protein levels in cDKO mice (*p* < 0.01). It should be noted that NaB-treated cDKO mice showed similar Ac-H3 protein levels compared with Veh-treated WT mice.

**Figure 7:**
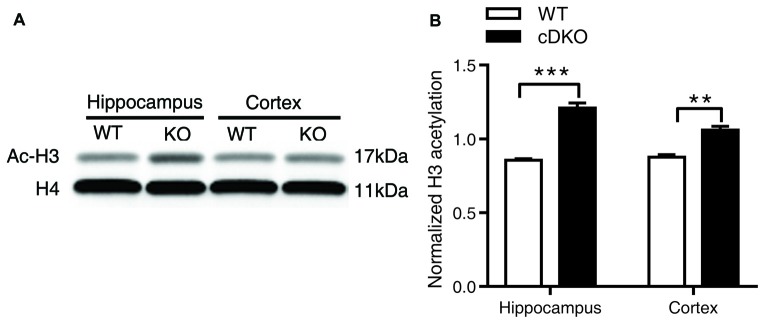
Ac-H3 was increased in the cortex and hippocampus of cDKO mice. **(A)** Western blot analysis of Ac-H3 in the cortex and hippocampus of 5-month-old cDKO mice and WT mice. Purified histone proteins from the cortex and hippocampus were immunoblotted for Ac-H3 and total histone H4 (H4, as loading control). **(B)** Quantification of Ac-H3 levels in **(A)**. Normalized H3 acetylation was calculated by dividing the band density of Ac-H3 by the band density of H4 for each sample. Data was expressed as mean ± SEM. Two tailed student’s *t*-test. **P < 0.01, ***P < 0.001.

Together, we surprisingly found that in Veh-treated group, histone H3 acetylation was significantly increased in the cortex and hippocamus of cDKO mice compared to the WT mice. We next tested whether the histone acetylation levels were changed in naive cDKO mice. Our western blot results showed that cDKO mice displayed elevated levels of Ac-H3 protein in the cortex and hippocampus relative to the WT mice (Figure 7). These data suggest that dysregulation of histone acetylation may contribute to the memory impairment observed in cDKO mice and can be rescued by HDAC inhibitor treatment.

## Discussion

Consistent with the previous study (Saura et al., 2004), we found that cDKO mice showed only contextual fear memory impairment in a 24-h retention test at 5-month-old age. Furthermore, cDKO mice exhibited more severe deficits in both contextual and cued fear memory when tested in a 1-month retention test in fear conditioning at 6-month-old age.

Accumulating evidences have shown that HDAC inhibitors have potential therapeutic roles and can restore memory function in several AD mouse models with amyloid deposition (Francis et al., 2009; Ricobaraza et al., 2009; Kilgore et al., 2010; Govindarajan et al., 2011; Sung et al., 2013). In this study, we evaluated whether HDAC inhibitors also have beneficial effects on PS cDKO mice—an Aβ-independent mouse model of AD-like phenotypes but with no amyloid deposition. Our study demonstrated that chronic systemic administration of HDAC inhibitor NaB enabled the recovery of impaired contextual fear memory in a 24-h retention test in cDKO mice. Using another corhort of mice to assess whether NaB treatment had long-lasting beneficial effects in cDKO mice, we found that 1 month after NaB treatment withdrawl, previous NaB treatment could still improve contextual fear memory in WT mice while having no impact on the memory deficits in cDKO mice. Our results indicate that the beneficial effect of a 3-week NaB treatment on memory deficits is not long-lasting, suggesting that NaB may have to be given to cDKO mice throughout their whole lifetime.

Progressive cortical shrinkage, accompanied by progressive neuron loss and age-dependent inflammatory response, was observed in cDKO mice at 6 and 9 months of age (Feng et al., 2004; Saura et al., 2004). In this study, we also observed decreased synaptic number in cDKO mice, but NaB had no effect on the reduction in synapse number and cortical atrophy. In addition, NaB treatment had no effect on the cell proliferation but increased newborn neurons in DG of cDKO mice. These results also indicate that the beneficial role of NaB treatment on associated fear memory should not necessarily rely on the restoration of neurons and their connections.

Accumulation of hyperphosphorylated tau is related to the cognitive impairment in AD patients and is a key hallmark of AD. Aberrantly hyperphosphorylated tau can be decreased to WT level in the hippocampus of 16-month-old Tg2576 transgenic mice by HDAC inhibitor 4-PBA treatment (Ricobaraza et al., 2009). Our study demonstrated that the phosphorylation of tau was increased in the cortex in 5-month-old cDKO mice and significantly reduced by NaB treatment. It has been shown that tau acetylation is elevated in the frontal cortex of AD patients and tau acetylation plays a role in inhibition of degradation of phosphorylated tau (Min et al., 2010). Future studies examining whether tau acetylation contributes to accumulation of hyperphosphorylated tau in the cortex of cDKO mice would be important.

Although the amyloid hypothesis has prevailed for many years, increasing evidence has supported the role of neuroinflammation in the AD etiology, which could be a promising therapeutic target in recent years. Astrocytes and microglia are activated in the brain of AD patients, correlated to cognitive impairment (Akiyama et al., 2000; Verkhratsky et al., 2010; Acosta et al., 2017). Astrocyte, once activated, results in the upregulation of cytokines, complement, acute phase reactants, and inflammatory mediators that contribute to neurodegeneration. The marker of reactive astrocyte GFAP was dramatically elevated in cDKO mice, which was significantly attenuated by NaB treatment in the hippocampus and cortex of cDKO mice. In parallel, further GO classification analysis for RNA-seq data has identified that lots of inflammation and immune related genes were altered in the forebrain of cDKO mice by NaB treatment. KEGG pathway enrichment analysis also highlighted the role of NaB in cytokine-cytokine receptor interaction and neuroactive ligand-receptor interaction signaling.

Previous studies have already employed microarray analysis to dissect the mechanism underlying memory deficits and neurogeneration and the molecular mechanism underlying the environmental enrichment and calorie restriction alleviated neuropathology in cDKO mice (Beglopoulos et al., 2004; Dong et al., 2007; Wu et al., 2008). All these studies found that a robust upregulation of a number of inflammatory and immunity related genes in the brain of cDKO mice and the expression of these genes could be decreased by both environment enrichment and calorie restriction. Among these inflammatory and immunity related genes, notably a high number of chemokine (C-C motif) ligand genes, cathepsin genes, complement component genes and S100 calcium binding protein A9 (S100a9) were upregulated in cDKO mice. It was interesting to note that our RNA-seq results were consistent with microarray findings that treatment with environmental enrichment and calorie restriction, our results showed that application of NaB also significantly down-regulated these genes (Supplementary Table S2). Most notably, chemokine (C-C motif) ligand 4 (Ccl4) and S100a9 were found dramatically increased in the brain of cDKO mice, which were down-regulated by both environment enrichment and calorie restriction (Dong et al., 2007; Wu et al., 2008), while in our study, both genes were also down-regulated by NaB treatment. It has been shown that several AD-like mouse models with hyperphosphorylated tau and/or increased GFAP levels had significantly higher levels of Ccl4 (one of inflammatory markers, also known as MIP-1β; Dong et al., 2007; Javidnia et al., 2017), which might be a potential cerebrospinal fluid biomarker of AD (Trombetta et al., 2018). S100a9, as an inflammation-associated calcium binding protein, was proposed to participate in inflammatory-mediated events, intracellular calcium increase and iNOS generation contributing to neurodegeneration. It has previously been shown to increase its expression in the brains of sporadic and familial AD patients (Shepherd et al., 2006) as well as AD mouse models (Ha et al., 2010). Both S100a9 knockdown and knockout attenuated spatial learning and memory deficits in Tg2576 AD mice (Ha et al., 2010; Kim et al., 2014). Its knockout also decreased amyloid beta peptide neuropathology including reduction of phophorylated tau and regulated expression of inflammatory related genes (Kim et al., 2014). Our results indicate that NaB chronic administration ameliorated memory deficits in cDKO mice, perhaps through regulating the astrocyte and microglia function, resulting in a decreased expression of GFAP, phophorylated tau, pro-inflammatory protein S100a9 and inflammatory marker Ccl4. This was accompanied by a modulation in the inflammation and immune response pathway in the brain of cDKO mice (Yin et al., 2017).

Furthermore, it has been reported that environmental enrichment could mimic the effect of HDAC inhibitor, probably by increasing histone 3 and 4 acetylation, restored the impaired learning and long-term memories in a neurodegenerative mice, which had already developed the significant brain atrophy and neuronal loss (Fischer et al., 2007). In addition, a recent study has shown that calorie restriction can increase the level of an endogenous HDAC inhibitor β-Hydroxybutyrate, which has similar structure and effects on histone H3 acetylation as NaB (Shimazu et al., 2013; Choudhary et al., 2014). Thus, enrichment, calorie restriction and HDAC inhibition by NaB are likely to regulate relatively specific genes and shared similar mechanism of ameliorating the neurodegenerative phenotypes in cDKO mice. And it is important to note that the observed improvement of memory performance by NaB treatment in cDKO mice could be a combination of acetylation of histone and non-histone proteins, because HAT and HDACs also have non-histone substrates, for example, Tau, GFAP, Nuclear factor kappa B (NF-κB) p65/p50 and α-Tubulin (Lopez-Atalaya and Barco, 2014).

In addition, it has been well shown that dysregulation of histone acetylation homeostasis is associated with the memory deficit and the treatment with HDAC inhibitors can increase histone H3/H4 acetylation as well as memory (Lu et al., 2015). However, there are no consistent results for the alteration of basal histone acetylation in AD patients’ brains (Sanchez-Mut and Gräff, 2015). In several AD mouse models, histone acetylation level was not altered, e.g., in APP/PS1 mice (H3 and H4; Francis et al., 2009; Kilgore et al., 2010) and 3xTg-AD mice (H3; Cadena-del-Castillo et al., 2014). But when mice were subjected to fear conditioning learning paradigms, histone H4 acetylation was increased in WT mice but not in APP/PS1 mice, which showed memory deficits; and HDAC inhibitor TSA rescued contextual fear memory by increasing H4 acetylation level (Francis et al., 2009). Our study demonstrated that both naive cDKO mice and cDKO mice after a fear conditioning learning task displayed an enhanced protein expression level of H3 acetylation in hippocampus and cortex. However, surprisingly, treatment with chronic NaB administration had opposite effects on the hippocampus of WT and cDKO mice: inducing acetylation of histone H3 in WT mice but decreasing in cDKO mice, while having no effect on the cortex. Nevertheless, chronic treatment of NaB, which enables the recovery of memory deficit, rescue of the inflammation and tau hyperphosphorylation phenotypes in the cDKO mice, at least in part, might be mediated by the reduction in H3 acetylation. Histone acetylation is a dynamic balance mediated by HAT and HDAC. Based on our findings, we propose that elevation of H3 acetylation in cDKO mice might be mediated by increased HAT activity and/or by reducing the ability of HDAC to deacetylate in chromatin. This increase is a consequence of changes to the HAT/HDAC themselves or their substrates, including nuclear histones or cytoplasmic non-histone proteins following double knockout PS1 and PS2 gene.

HDAC inhibitor also involves in the regulation of inflammation. It has been reported that orally administration of HDAC inhibitor MS-275 reduced amyloid plaque load and neuroinflammation, especially ameliorated microglial activation in APP/PS1-21 mice (Zhang and Schluesener, 2013). The anti-inflammatory property of HDAC inhibitor contributes to the reduction of tau phosphorylation (Tweedie et al., 2012). Changes of histone H3 lysine 27 (H3K27) acetylation were also found at regulatory regions of not only synapse plasticity and learning related genes, but also immune and inflammatory response genes in the AD mouse model (Gjoneska et al., 2015). In PS cDKO mice, which have no excessive amyloid deposition but with obvious astrocyte activation and increased inflammation response, NaB may exert an inhibitory effect on neuroinflammation, which may contribute to the reduced phosphorylated tau and reinstated memory function. The beneficial effects of NaB are likely to be mediated by attenuating striking astrogliosis, following downregulation of inflammatory and immune related genes and upregulation of anti-inflammatory genes, as a result of decreased abnormal H3 hyperacetylation. Meanwhile, we also don’t exclude the effects of NaB on genes associated with synaptic plasticity and memory and other events involved in memory process.

In summary, our results demonstrated that chronic NaB administration could reverse impaired contextual memory and ameliorated neuroinflammation in our amyloid independent AD mouse model, thus strongly supporting a role for the pathological synergistic interaction between dysregulation of histone acetylation and neuroinflammtion in AD pathology. Our studies suggest that NaB might be a potential drug for treating AD.

## Author Contributions

TC, XZhou and XZheng performed experiments and analyzed data. YC performed experiments. JT had intellectual input into the manuscript preparation. CL and HW contributed to the funding of the project, the experimental design, the manuscript preparation and manuscript writing.

## Conflict of Interest Statement

The authors declare that the research was conducted in the absence of any commercial or financial relationships that could be construed as a potential conflict of interest.

## Abbreviations

Aβ: β-Amyloid
Ac-H3: acetylated histone 3
AD: Alzheimer’s disease
cDKO mice: presenilin-1 and presenilin-2 conditional double knockout mice
GFAP: glial fibrillary acidic protein
H3: histone 3
H4: histone 4
HDAC: histone deacetylase
NaB: sodium butyrate
PS1: Presenilin 1
PS2: Presenilin 2.
