# Supplementary material for: Histone Deacetylase Inhibitor Alleviates the Neurodegenerative Phenotypes and Histone Dysregulation in Presenilins-Deficient Mice
Source: Front Aging Neurosci. 2018 May 15;10:137. doi: 10.3389/fnagi.2018.00137 (PMC5962686; doi:10.3389/fnagi.2018.00137)
Supplement: Supplementary file 1 [file Table_1.pdf]

Histone deacetylase inhibitor alleviates the neurodegenerative phenotypes and histone dysregulation in presenilins-deficient mice

Ting Cao[1†], Xiaojuan Zhou[1†,] Xianjie Zheng[1†], Yue Cui[1], Joe Z. Tsien[2], Chunxia Li[1*], Huimin Wang[1, 3, 4*]

[†] **These authors have contributed equally to this work.**
***Correspondence: Dr. Chunxia Li, cxli@brain.ecnu.edu.cn; Dr. Huimin Wang hmwang@nbic.ecnu.edu.cn**

[1] Shanghai Key Laboratory of Brain Functional Genomics, Key Laboratory of Brain Functional Genomics, Ministry of Education, School of Psychology and Cognitive Science, East China Normal University, Shanghai, China.
[2] Brain and Behavior Discovery Institute and Department of Neurology, Medical College of Georgia at Augusta University, Augusta, USA.
[3] NYU-ECNU Institute of Brain and Cognitive Science at NYU Shanghai, Shanghai, China.
[4] Shanghai Changning-ECNU Mental Health Center, Shanghai, China.

Supplementary TABLE S1: Primer sequences used for Real-Time PCR.

Gene name	Forward primer	Reverse primer
Ccl4	TGC TCG TGG CTG CCT TCT	CTG CCG GGA GGT GTA AGA GA
S100A9	CGCAGCATAACCACCATCAT	ACTTCCCACAGCCTTTGCC
GFAP	CGGAGACGCATCACCTCTG	AGGGAGTGGAGGAGTCATTCG
GAPDH	AGGAGCGAGACCCCACTAACAT	GTGATGGCATGGACTGTGGT
