# Supplementary material for: Histone Deacetylase Inhibitor Alleviates the Neurodegenerative Phenotypes and Histone Dysregulation in Presenilins-Deficient Mice
Source: Front Aging Neurosci. 2018 May 15;10:137. doi: 10.3389/fnagi.2018.00137 (PMC5962686; doi:10.3389/fnagi.2018.00137)
Supplement: Supplementary file 2 [file Table_2.pdf]

Histone deacetylase inhibitor alleviates the neurodegenerative phenotypes and histone dysregulation in presenilins-deficient mice

Ting Cao[1†], Xiaojuan Zhou[1†,] Xianjie Zheng[1†], Yue Cui[1], Joe Z. Tsien[2], Chunxia Li[1*], Huimin Wang[1, 3, 4*]

[†] **These authors have contributed equally to this work.**
***Correspondence: Dr. Chunxia Li, cxli@brain.ecnu.edu.cn; Dr. Huimin Wang hmwang@nbic.ecnu.edu.cn**

[1] Shanghai Key Laboratory of Brain Functional Genomics, Key Laboratory of Brain Functional Genomics, Ministry of Education, School of Psychology and Cognitive Science, East China Normal University, Shanghai, China.
[2] Brain and Behavior Discovery Institute and Department of Neurology, Medical College of Georgia at Augusta University, Augusta, USA.
[3] NYU-ECNU Institute of Brain and Cognitive Science at NYU Shanghai, Shanghai, China.
[4] Shanghai Changning-ECNU Mental Health Center, Shanghai, China.

Supplementary Table S2: The differential expressions of genes in the forebrain of NaB-treated cDKO mice

gene_id	gene_name	Description	cDKO+NaB_FPKM	cDKO+Veh_FPKM	log2(Fold_change)
11287	Pzp	pregnancy zone protein	1.00E-05	0.0236919	-1.12E+01
11298	Aanat	arylalkylamine N-acetyltransferase	1.00E-05	0.120599	-1.36E+01
11425	Apoc4	apolipoprotein C-IV	0.653894	0.323093	1.02E+00
11443	Chrnb1	cholinergic receptor, nicotinic, beta polypeptide 1 (muscle)	0.249182	0.582992	-1.23E+00
11450	Adipoq	adiponectin, C1Q and collagen domain containing	1.00E-05	0.102171	-1.33E+01
11474	Actn3	actinin alpha 3	0.689836	0.327955	1.07E+00
11486	Ada	adenosine deaminase	0.696215	0.215729	1.69E+00
11498	Adam4	a disintegrin and metallopeptidase domain 4	0.177311	0.0480351	1.88E+00
11499	Adam5	a disintegrin and metallopeptidase domain 5	0.244633	0.0877066	1.48E+00

11522	Adh1	alcohol dehydrogenase 1 (class I)	0.879219	0.401301	1.13E+00
11602	Angpt4	angiopoietin 4	1.00E-05	0.0824421	-1.30E+01
11604	Agrp	agouti related protein	1.00E-05	0.71336	-1.61E+01
11607	Agtr1a	angiotensin II receptor, type 1a	0.513456	0.236824	1.12E+00
11609	Agtr2	angiotensin II receptor, type 2	0.636941	0.307176	1.05E+00
11634	Aire	autoimmune regulator (autoimmune polyendocrinopathy candidiasis ectodermal dystrophy)	1.00E-05	0.155625	-1.39E+01
11670	Aldh3a1	aldehyde dehydrogenase family 3, subfamily A1	1.36944	0.557723	1.30E+00
11685	Alox12e	arachidonate lipoxygenase, epidermal	0.157323	0.0535339	1.56E+00
11698	Ambn	ameloblastin	1.00E-05	0.0687826	-1.27E+01
11705	Amh	anti-Mullerian hormone	0.547361	0.102966	2.41E+00
11720	Mat1a	methionine adenosyltransferase I, alpha	0.0947697	1.00E-05	1.32E+01
11811	Apobec2	apolipoprotein B mRNA editing enzyme, catalytic polypeptide 2	0.215166	0.442939	-1.04E+00
11814	Apoc3	apolipoprotein C-III	0.340834	0.687799	-1.01E+00
11818	Apoh	apolipoprotein H	0.0993539	0.629791	-2.66E+00
11828	Aqp3	aquaporin 3	0.15336	1.00E-05	1.39E+01
11832	Aqp7	aquaporin 7	0.152422	1.00E-05	1.39E+01
11854	Rhod	ras homolog gene family, member D	0.217153	0.657453	-1.60E+00
11923	Neurod4	neurogenic differentiation 4	0.0956605	0.21256	-1.15E+00
11924	Neurog2	neurogenin 2	0.522628	1.00E-05	1.57E+01
11925	Neurog3	neurogenin 3	1.00E-05	0.209591	-1.44E+01
11944	Atp4a	ATPase, H+/K+ exchanging, gastric, alpha polypeptide	0.242489	0.080479	1.59E+00
11998	Avp	arginine vasopressin	41.4849	19.6867	1.08E+00
12044	Bcl2a1a	B cell leukemia/lymphoma 2 related protein A1a	1.00E-05	0.171093	-1.41E+01

12045	Bcl2a1b	B cell leukemia/lymphoma 2 related protein A1b	0.691697	1.00E-05	1.61E+01
12051	Bcl3	B cell leukemia/lymphoma 3	0.873468	0.278489	1.65E+00
12061	Bdkrb1	bradykinin receptor, beta 1	1.00E-05	0.241836	-1.46E+01
12095	Bglap3	bone gamma-carboxyglutamate protein 3	0.551699	1.00E-05	1.58E+01
12155	Bmp15	bone morphogenetic protein 15	1.00E-05	0.133782	-1.37E+01
12160	Bmp5	bone morphogenetic protein 5	0.963161	0.460761	1.06E+00
12182	Bst1	bone marrow stromal cell antigen 1	0.044767	0.0963118	-1.11E+00
12223	Btc	betacellulin, epidermal growth factor family member	0.195444	0.471345	-1.27E+00
12228	Btg3	B cell translocation gene 3	0.130756	0.0175666	2.90E+00
12229	Btk	Bruton agammaglobulinemia tyrosine kinase	0.328503	0.707549	-1.11E+00
12235	Bub1	budding uninhibited by benzimidazoles 1 homolog (S. cerevisiae)	0.0233061	0.07676	-1.72E+00
12270	C4bp-ps1	complement component 4 binding protein, pseudogene 1	1.00E-05	0.175384	-1.41E+01
12273	C5ar1	complement component 5a receptor 1	0.410378	0.18168	1.18E+00
12310	Calca	calcitonin/calcitonin-related polypeptide, alpha	1.75085	0.561964	1.64E+00
12316	Aspm	asp (abnormal spindle)-like, microcephaly associated (Drosophila)	0.0706821	0.0109078	2.70E+00
12346	Car1	carbonic anhydrase 1	1.00E-05	0.105917	-1.34E+01
12352	Car5a	carbonic anhydrase 5a, mitochondrial	0.466814	1.00E-05	1.55E+01
12364	Casp12	caspase 12	0.114974	0.388272	-1.76E+00
12372	Casq1	calsequestrin 1	0.277402	0.121158	1.20E+00
12399	Runx3	runt related transcription factor 3	0.0345977	0.0697481	-1.01E+00
12442	Ccnb2	cyclin B2	0.452361	0.158722	1.51E+00
12458	Ccr6	chemokine (C-C motif) receptor 6	0.745518	1.82059	-1.29E+00
12479	Cd1d1	CD1d1 antigen	1.00E-05	0.380318	-1.52E+01

12480	Cd1d2	CD1d2 antigen	0.291904	0.0996631	1.55E+00
12503	Cd247	CD247 antigen	0.199021	0.0697629	1.51E+00
12506	Cd48	CD48 antigen	0.846316	1.83516	-1.12E+00
12507	Cd5	CD5 antigen	1.00E-05	0.0621327	-1.26E+01
12587	Mia	melanoma inhibitory activity	2.2441	0.981909	1.19E+00
12623	Ces1g	carboxylesterase 1G	1.00E-05	0.0490504	-1.23E+01
12638	Cftr	cystic fibrosis transmembrane conductance regulator	0.230109	0.102168	1.17E+00
12640	Cga	glycoprotein hormones, alpha subunit	8.57089	1.00E-05	1.97E+01
12642	Ch25h	cholesterol 25-hydroxylase	0.860114	0.377472	1.19E+00
12715	Ckm	creatine kinase, muscle	1.00E-05	0.0961584	-1.32E+01
12722	Clca1	chloride channel calcium activated 1	0.0971931	0.311258	-1.68E+00
12733	Clcnka	chloride channel Ka	0.324807	0.126896	1.36E+00
12739	Cldn3	claudin 3	0.672107	0.157071	2.10E+00
12763	Cmah	cytidine monophospho-N-acetylneuraminic acid hydroxylase	0.0641294	0.0234367	1.45E+00
12766	Cxcr3	chemokine (C-X-C motif) receptor 3	0.194817	1.00E-05	1.42E+01
12789	Cnga2	cyclic nucleotide gated channel alpha 2	0.0351853	0.112234	-1.67E+00
12796	Camp	cathelicidin antimicrobial peptide	1.00E-05	1.32023	-1.70E+01
12865	Cox7a1	cytochrome c oxidase subunit VIIa 1	4.06017	8.21917	-1.02E+00
12873	Cpa3	carboxypeptidase A3, mast cell	1.00E-05	0.0791352	-1.30E+01
12902	Cr2	complement receptor 2	0.0150463	0.1507	-3.32E+00
12954	Cryaa	crystallin, alpha A	1.00E-05	0.149882	-1.39E+01
12957	Cryba1	crystallin, beta A1	1.00E-05	0.227583	-1.45E+01
13025	Ctla2b	cytotoxic T lymphocyte-associated protein 2 beta	1.33708	0.590013	1.18E+00
13035	Ctsg	cathepsin G	1.00E-05	0.136556	-1.37E+01
13067	Cyct	cytochrome c, testis	1.00E-05	0.189297	-1.42E+01

13074	Cyp17a1	cytochrome P450, family 17, subfamily a, polypeptide 1	1.00E-05	0.0634266	-1.26E+01
13076	Cyp1a1	cytochrome P450, family 1, subfamily a, polypeptide 1	0.0431784	0.136157	-1.66E+00
13082	Cyp26a1	cytochrome P450, family 26, subfamily a, polypeptide 1	0.244342	1.00E-05	1.46E+01
13087	Cyp2a5	cytochrome P450, family 2, subfamily a, polypeptide 5	0.146318	0.308364	-1.08E+00
13106	Cyp2e1	cytochrome P450, family 2, subfamily e, polypeptide 1	1.00E-05	0.136478	-1.37E+01
13118	Cyp4a12b	cytochrome P450, family 4, subfamily a, polypeptide 12B	1.00E-05	0.0538488	-1.24E+01
13120	Cyp4b1	cytochrome P450, family 4, subfamily b, polypeptide 1	0.425346	0.144964	1.55E+00
13166	Dbh	dopamine beta hydroxylase	0.327444	0.0551129	2.57E+00
13184	Dcpp1	demilune cell and parotid protein 1	0.445483	1.00E-05	1.54E+01
13214	Defb1	defensin beta 1	0.676673	1.49153	-1.14E+00
13363	Dhh	desert hedgehog	0.204055	1.00E-05	1.43E+01
13393	Dlx3	distal-less homeobox 3	1.00E-05	0.0539402	-1.24E+01
13394	Dlx4	distal-less homeobox 4	0.145747	1.00E-05	1.38E+01
13409	Tmc1	transmembrane channel-like gene family 1	0.141825	0.0621609	1.19E+00
13419	Dnase1	deoxyribonuclease I	0.101911	0.627634	-2.62E+00
13421	Dnase1l3	deoxyribonuclease 1-like 3	1.00E-05	0.0317531	-1.16E+01
13436	Dnmt3b	DNA methyltransferase 3B	0.0855167	0.191744	-1.16E+00
13449	Dok2	docking protein 2	0.323983	0.0804101	2.01E+00
13479	Dpep1	dipeptidase 1 (renal)	0.0523733	0.159913	-1.61E+00
13487	Slc26a3	solute carrier family 26, member 3	0.0386697	0.125565	-1.70E+00
13491	Drd4	dopamine receptor D4	0.71177	0.164375	2.11E+00
13505	Dsc1	desmocollin 1	1.00E-05	0.0554559	-1.24E+01
13516	Epyc	epiphycan	0.596596	0.265777	1.17E+00
13590	Lefty1	left right determination factor 1	0.588311	0.190619	1.63E+00
13605	Ect2	ect2 oncogene	0.102427	0.229434	-1.16E+00

13706	Cela2a	chymotrypsin-like elastase family, member 2A	1.00E-05	0.174504	-1.41E+01
13835	Epha1	Eph receptor A1	0.0698884	0.212755	-1.61E+00
14063	F2rl1	coagulation factor II (thrombin) receptor-like 1	0.249586	0.0523112	2.25E+00
14065	F2rl3	coagulation factor II (thrombin) receptor-like 3	0.177108	1.00E-05	1.41E+01
14068	F7	coagulation factor VII	0.0627758	0.129568	-1.05E+00
14103	Fasl	Fas ligand (TNF superfamily, member 6)	1.00E-05	0.0605708	-1.26E+01
14120	Fbp2	fructose bisphosphatase 2	0.0995929	0.204731	-1.04E+00
14133	Fcna	ficolin A	0.27573	0.0957209	1.53E+00
14171	Fgf17	fibroblast growth factor 17	0.266016	0.0885084	1.59E+00
14174	Fgf3	fibroblast growth factor 3	0.25549	0.632307	-1.31E+00
14178	Fgf7	fibroblast growth factor 7	0.646269	0.126503	2.35E+00
14204	Il4i1	interleukin 4 induced 1	0.833243	0.31503	1.40E+00
14233	Foxi1	forkhead box I1	0.100669	1.00E-05	1.33E+01
14239	Foxs1	forkhead box S1	0.771229	0.374617	1.04E+00
14256	Flt3l	FMS-like tyrosine kinase 3 ligand	2.80663	0.715781	1.97E+00
14276	Folr2	folate receptor 2 (fetal)	0.355335	1.07802	-1.60E+00
14293	Fpr1	formyl peptide receptor 1	1.00E-05	0.111446	-1.34E+01
14308	Fshb	follicle stimulating hormone beta	0.626181	1.00E-05	1.59E+01
14313	Fst	follistatin	0.413954	0.941495	-1.19E+00
14317	Ftcd	formiminotransferase cyclodeaminase	0.0910771	1.00E-05	1.32E+01
14343	Fut1	fucosyltransferase 1	0.0438256	0.0926623	-1.08E+00
14347	Fut7	fucosyltransferase 7	0.256865	0.0855805	1.59E+00
14408	Gabrr1	gamma-aminobutyric acid (GABA) C receptor, subunit rho 1	1.00E-05	0.164434	-1.40E+01
14409	Gabrr2	gamma-aminobutyric acid (GABA) C receptor, subunit rho 2	0.743638	0.344151	1.11E+00
14422	B4galnt2	beta-1,4-N-acetyl-galactosaminyl transferase 2	0.122915	1.00E-05	1.36E+01

14429	Galr3	galanin receptor 3	1.00E-05	0.241729	-1.46E+01
14539	Opn1mw	opsin 1 (cone pigments), medium-wave-sensitive (color blindness, deutan)	1.00E-05	0.497567	-1.56E+01
14559	Gdf1	growth differentiation factor 1	0.412678	8.19046	-4.31E+00
14566	Gdf9	growth differentiation factor 9	0.286735	0.585457	-1.03E+00
14598	Ggt1	gamma-glutamyltransferase 1	0.945533	0.406638	1.22E+00
14599	Gh	growth hormone	156.315	0.187316	9.70E+00
14601	Ghrh	growth hormone releasing hormone	1.00E-05	1.92312	-1.76E+01
14602	Ghrhr	growth hormone releasing hormone receptor	0.187417	1.00E-05	1.42E+01
14611	Gja3	gap junction protein, alpha 3	0.192425	1.00E-05	1.42E+01
14613	Gja5	gap junction protein, alpha 5	0.199968	0.412807	-1.05E+00
14621	Gjb4	gap junction protein, beta 4	0.175526	1.00E-05	1.41E+01
14663	Glycam1	glycosylation dependent cell adhesion molecule 1	0.633185	0.228566	1.47E+00
14685	Gnat1	guanine nucleotide binding protein, alpha transducing 1	1.00E-05	0.0529071	-1.24E+01
14686	Gnat2	guanine nucleotide binding protein, alpha transducing 2	0.0596944	0.124897	-1.07E+00
14711	Gnmt	glycine N-methyltransferase	0.143453	0.421409	-1.55E+00
14725	Lrp2	low density lipoprotein receptor-related protein 2	0.0486056	0.021755	1.16E+00
14727	Gp49a	glycoprotein 49 A	0.160594	0.338108	-1.07E+00
14728	Lilrb4	leukocyte immunoglobulin-like receptor, subfamily B, member 4	0.0716109	0.386026	-2.43E+00
14829	Grpr	gastrin releasing peptide receptor	0.596118	0.295749	1.01E+00
14857	Gsta1	glutathione S-transferase, alpha 1 (Ya)	0.421895	0.14924	1.50E+00
14934	Gypa	glycophorin A	1.00E-05	0.0667364	-1.27E+01
14938	Gzma	granzyme A	0.243615	1.00E-05	1.46E+01
14955	H19	H19 fetal liver mRNA	1.00E-05	0.136793	-1.37E+01

14980	H2-L	histocompatibility 2, D region locus L	1.66441	1.00E-05	1.73E+01
15000	H2-DMb2	histocompatibility 2, class II, locus Mb2	0.355234	1.24004	-1.80E+00
15024	H2-T10	histocompatibility 2, T region locus 10	0.236576	0.492392	-1.06E+00
15061	Ifi44l	interferon-induced protein 44 like	0.288505	0.103215	1.48E+00
15109	Hal	histidine ammonia lyase	0.174396	0.044756	1.96E+00
15112	Hao1	hydroxyacid oxidase 1, liver	0.111922	1.00E-05	1.35E+01
15122	Hba-a1	hemoglobin alpha, adult chain 1	335.37	119.342	1.49E+00
15364	Hmga2	high mobility group AT-hook 2	1.00E-05	0.0690087	-1.28E+01
15373	Hmx3	H6 homeobox 3	0.336648	0.157281	1.10E+00
15376	Foxa2	forkhead box A2	0.0718376	0.215152	-1.58E+00
15464	Hrc	histidine rich calcium binding protein	0.0635209	0.141285	-1.15E+00
15494	Hsd3b3	hydroxy-delta-5-steroid dehydrogenase, 3 beta- and steroid delta-isomerase 3	1.00E-05	0.0781976	-1.29E+01
15559	Htr2b	5-hydroxytryptamine (serotonin) receptor 2B	0.0588461	0.124516	-1.08E+00
15567	Slc6a4	solute carrier family 6 (neurotransmitter transporter, serotonin), member 4	1.66241	0.728468	1.19E+00
15945	Cxcl10	chemokine (C-X-C motif) ligand 10	0.251745	0.526001	-1.06E+00
16006	Igfbp1	insulin-like growth factor binding protein 1	1.00E-05	0.093949	-1.32E+01
16156	Il11	interleukin 11	0.0612714	0.259489	-2.08E+00
16163	Il13	interleukin 13	1.00E-05	0.125511	-1.36E+01
16176	Il1b	interleukin 1 beta	0.0856973	0.362873	-2.08E+00
16178	Il1r2	interleukin 1 receptor, type II	0.084118	0.438355	-2.38E+00
16181	Il1rn	interleukin 1 receptor antagonist	0.0508609	0.162372	-1.67E+00
16182	Il18r1	interleukin 18 receptor 1	0.0680562	1.00E-05	1.27E+01
16184	Il2ra	interleukin 2 receptor, alpha chain	0.0480511	1.00E-05	1.22E+01

16185	Il2rb	interleukin 2 receptor, beta chain	1.00E-05	0.204804	-1.43E+01
16191	Il5	interleukin 5	0.15505	1.00E-05	1.39E+01
16193	Il6	interleukin 6	0.2058	1.00E-05	1.43E+01
16205	Gimap1	GTPase, IMAP family member 1	1.70998	0.831664	1.04E+00
16364	Irf4	interferon regulatory factor 4	0.096826	0.0257464	1.91E+00
16369	Irs3	insulin receptor substrate 3	1.00E-05	0.122092	-1.36E+01
16398	Itga2	integrin alpha 2	0.0242507	0.0517092	-1.09E+00
16427	Itih4	inter alpha-trypsin inhibitor, heavy chain 4	0.0685383	1.00E-05	1.27E+01
16516	Kcnj15	potassium inwardly-rectifying channel, subfamily J, member 15	1.00E-05	0.022898	-1.12E+01
16534	Kcnn4	potassium intermediate/small conductance calcium-activated channel, subfamily N, member 4	0.439888	0.145731	1.59E+00
16571	Kif4	kinesin family member 4	0.136665	0.297006	-1.12E+00
16619	Klk1b27	kallikrein 1-related peptidase b27	1.00E-05	0.154404	-1.39E+01
16621	Klkb1	kallikrein B, plasma 1	0.0419321	0.181719	-2.12E+00
16633	Klra2	killer cell lectin-like receptor, subfamily A, member 2	0.121536	1.00E-05	1.36E+01
16664	Krt14	keratin 14	0.150845	1.00E-05	1.39E+01
16666	Krt16	keratin 16	1.00E-05	0.0768832	-1.29E+01
16670	Krt32	keratin 32	1.00E-05	0.0692348	-1.28E+01
16840	Lect1	leukocyte cell derived chemotaxin 1	1.23423	0.572467	1.11E+00
16854	Lgals3	lectin, galactose binding, soluble 3	1.5697	0.753135	1.06E+00
16871	Lhx3	LIM homeobox protein 3	0.222737	1.00E-05	1.44E+01
16872	Lhx4	LIM homeobox protein 4	0.136215	1.00E-05	1.37E+01
16992	Lta	lymphotoxin A	1.00E-05	0.294091	-1.48E+01
16997	Ltbp2	latent transforming growth factor beta binding protein 2	0.0196462	0.0575602	-1.55E+00
17002	Ltf	lactotransferrin	1.00E-05	0.163197	-1.40E+01

17057	Klrb1a	killer cell lectin-like receptor subfamily B member 1A	1.00E-05	0.100422	-1.33E+01
17059	Klrb1c	killer cell lectin-like receptor subfamily B member 1C	0.0423548	0.0907907	-1.10E+00
17068	Ly6d	lymphocyte antigen 6 complex, locus D	0.51426	0.253135	1.02E+00
17082	Il1rl1	interleukin 1 receptor-like 1	0.105869	0.0228621	2.21E+00
17087	Ly96	lymphocyte antigen 96	1.80817	0.646217	1.48E+00
17147	Mageb3	melanoma antigen, family B, 3	1.00E-05	0.10696	-1.34E+01
17150	Mfap2	microfibrillar-associated protein 2	0.775846	2.56192	-1.72E+00
17172	Ascl1	achaete-scute complex homolog 1 (Drosophila)	1.76178	3.90461	-1.15E+00
17180	Matn1	matrilin 1, cartilage matrix protein	0.497041	0.212179	1.23E+00
17182	Matn3	matrilin 3	1.00E-05	0.172508	-1.41E+01
17189	Mb	myoglobin	0.238471	1.00E-05	1.45E+01
17199	Mc1r	melanocortin 1 receptor	1.00E-05	0.0339381	-1.17E+01
17200	Mc2r	melanocortin 2 receptor	1.00E-05	0.0898276	-1.31E+01
17203	Mc5r	melanocortin 5 receptor	1.00E-05	0.27223	-1.47E+01
17240	Mdfi	MyoD family inhibitor	0.12103	0.348311	-1.53E+00
17292	Mesp1	mesoderm posterior 1	0.370939	1.00E-05	1.52E+01
17293	Mesp2	mesoderm posterior 2	0.966705	0.345367	1.48E+00
17329	Cxcl9	chemokine (C-X-C motif) ligand 9	0.0405739	0.129785	-1.68E+00
17341	Bhlha15	basic helix-loop-helix family, member a15	0.0756847	1.00E-05	1.29E+01
17364	Trpm1	transient receptor potential cation channel, subfamily M, member 1	0.0799621	1.00E-05	1.30E+01
17386	Mmp13	matrix metallopeptidase 13	1.00E-05	0.0425885	-1.21E+01
17528	Mpz	myelin protein zero	0.147808	0.371164	-1.33E+00
17703	Msx3	homeobox, msh-like 3	1.00E-05	0.125934	-1.36E+01
17771	Mtl5	metallothionein-like 5, testis-specific (tesmin)	1.00E-05	0.133145	-1.37E+01

17842	Mup3	major urinary protein 3	0.127642	0.279984	-1.13E+00
17843	Mup4	major urinary protein 4	0.134483	0.295917	-1.14E+00
17857	Mx1	myxovirus (influenza virus) resistance 1	0.0756022	0.235293	-1.64E+00
17865	Mybl2	myeloblastosis oncogene-like 2	0.129237	0.297858	-1.20E+00
17877	Myf5	myogenic factor 5	1.00E-05	0.0691819	-1.28E+01
17884	Myh4	myosin, heavy polypeptide 4, skeletal muscle	0.0441376	0.110551	-1.32E+00
17885	Myh8	myosin, heavy polypeptide 8, skeletal muscle, perinatal	0.0721793	0.0309896	1.22E+00
17907	Mylpf	myosin light chain, phosphorylatable, fast skeletal muscle	0.153657	0.661732	-2.11E+00
17927	Myod1	myogenic differentiation 1	1.00E-05	0.281147	-1.48E+01
17940	Naip1	NLR family, apoptosis inhibitory protein 1	0.0386948	1.00E-05	1.19E+01
17960	Nat1	N-acetyl transferase 1	0.871217	1.74565	-1.00E+00
18022	Nfe2	nuclear factor, erythroid derived 2	1.00E-05	0.393208	-1.53E+01
18071	Nhlh1	nescient helix loop helix 1	0.617416	0.125438	2.30E+00
18094	Nkx2-9	NK2 transcription factor related, locus 9 (Drosophila)	1.00E-05	0.455487	-1.55E+01
18106	Cd244	CD244 natural killer cell receptor 2B4	0.0560747	1.00E-05	1.25E+01
18113	Nnmt	nicotinamide N-methyltransferase	1.29931	0.136736	3.25E+00
18126	Nos2	nitric oxide synthase 2, inducible	0.0570185	0.116625	-1.03E+00
18169	Npy6r	neuropeptide Y receptor Y6	1.00E-05	0.0455974	-1.22E+01
18241	Gpr143	G protein-coupled receptor 143	0.170345	1.00E-05	1.41E+01
18285	Odf1	outer dense fiber of sperm tails 1	1.00E-05	0.1396	-1.38E+01
18292	Sebox	SEBOX homeobox	2.24977	0.972583	1.21E+00
18392	Orc1	origin recognition complex, subunit 1	0.0693069	1.00E-05	1.28E+01
18436	P2rx1	purinergic receptor P2X, ligand-gated ion channel, 1	0.350999	1.00E-05	1.51E+01
18478	Pah	phenylalanine hydroxylase	0.210041	0.0565129	1.89E+00
18503	Pax1	paired box gene 1	1.00E-05	0.0622063	-1.26E+01

18504	Pax2	paired box gene 2	0.105994	1.00E-05	1.34E+01
18509	Pax7	paired box gene 7	0.114139	0.263107	-1.20E+00
18510	Pax8	paired box gene 8	0.0474317	0.192764	-2.02E+00
18587	Pde6b	phosphodiesterase 6B, cGMP, rod receptor, beta polypeptide	0.0350343	0.14896	-2.09E+00
18601	Padi3	peptidyl arginine deiminase, type III	0.0851078	1.00E-05	1.31E+01
18618	Pemt	phosphatidylethanolamine N-methyltransferase	2.69129	0.909856	1.56E+00
18712	Pim1	proviral integration site 1	2.26079	1.00889	1.16E+00
18725	Pira2	paired-Ig-like receptor A2	0.122798	1.00E-05	1.36E+01
18729	Pira6	paired-Ig-like receptor A6	1.00E-05	0.0680472	-1.27E+01
18781	Pla2g2c	phospholipase A2, group IIC	0.0752104	0.23247	-1.63E+00
18787	Serpine1	serine (or cysteine) peptidase inhibitor, clade E, member 1	0.889248	0.417737	1.09E+00
18815	Plg	plasminogen	0.11361	1.00E-05	1.35E+01
18843	Bpifa1	BPI fold containing family A, member 1	0.526789	1.00E-05	1.57E+01
18935	Phox2b	paired-like homeobox 2b	0.0553227	0.112877	-1.03E+00
18947	Pnliprp2	pancreatic lipase-related protein 2	1.00E-05	0.0736179	-1.28E+01
18976	Pomc	pro-opiomelanocortin-alpha	15.8627	3.10041	2.36E+00
19059	Ppp3r2	protein phosphatase 3, regulatory subunit B, alpha isoform (calcineurin B, type II)	0.0334823	0.182456	-2.45E+00
19109	Prl	prolactin	32.6266	0.320348	6.67E+00
19119	Prm2	protamine 2	1.00E-05	0.26601	-1.47E+01
19153	Prx	periaxin	0.330821	0.147302	1.17E+00
19207	Ptch2	patched homolog 2	0.216308	0.758625	-1.81E+00
19219	Ptger4	prostaglandin E receptor 4 (subtype EP4)	0.965497	0.387203	1.32E+00
19222	Ptgir	prostaglandin I receptor (IP)	0.0360011	0.0733668	-1.03E+00
19288	Ptx3	pentraxin related gene	0.134565	0.352793	-1.39E+00

19329	Rab17	RAB17, member RAS oncogene family	1.00E-05	0.0802452	-1.30E+01
19369	Raet1b	retinoic acid early transcript beta	1.00E-05	0.176906	-1.41E+01
19660	Rbp2	retinol binding protein 2, cellular	1.00E-05	0.19765	-1.43E+01
19674	Rcvrn	recoverin	1.00E-05	0.251743	-1.46E+01
19700	Rem1	rad and gem related GTP binding protein 1	0.284352	0.945529	-1.73E+00
19733	Rgn	regucalcin	0.419311	0.076621	2.45E+00
19746	Rhd	Rh blood group, D antigen	0.0955616	0.287476	-1.59E+00
19752	Rnase1	ribonuclease, RNase A family, 1 (pancreatic)	0.138977	0.286641	-1.04E+00
19888	Rp1	retinitis pigmentosa 1 (human)	1.00E-05	0.0138254	-1.04E+01
19941	Rpl26	ribosomal protein L26	24.3187	11.1593	1.12E+00
20186	Nr1h4	nuclear receptor subfamily 1, group H, member 4	0.0579175	0.121346	-1.07E+00
20197	S100a3	S100 calcium binding protein A3	0.240457	0.482761	-1.01E+00
20201	S100a8	S100 calcium binding protein A8 (calgranulin A)	2.01591	7.02276	-1.80E+00
20202	S100a9	S100 calcium binding protein A9 (calgranulin B)	1.24338	4.04354	-1.70E+00
20208	Saa1	serum amyloid A 1	1.00E-05	0.243125	-1.46E+01
20210	Saa3	serum amyloid A 3	1.00E-05	0.298906	-1.49E+01
20231	Nkx1-2	NK1 transcription factor related, locus 2 (Drosophila)	0.078152	0.157927	-1.01E+00
20264	Scn10a	sodium channel, voltage-gated, type X, alpha	0.036543	0.075307	-1.04E+00
20278	Scnn1g	sodium channel, nonvoltage-gated 1 gamma	0.195332	0.0402014	2.28E+00
20293	Ccl12	chemokine (C-C motif) ligand 12	1.00E-05	1.54165	-1.72E+01
20296	Ccl2	chemokine (C-C motif) ligand 2	0.348521	1.00E-05	1.51E+01
20303	Ccl4	chemokine (C-C motif) ligand 4	0.51742	1.63438	-1.66E+00
20304	Ccl5	chemokine (C-C motif) ligand 5	0.341506	1.42303	-2.06E+00
20307	Ccl8	chemokine (C-C motif) ligand 8	0.322369	0.677304	-1.07E+00
20343	Sell	selectin, lymphocyte	1.00E-05	0.0982406	-1.33E+01

20344	Selp	selectin, platelet	1.00E-05	0.0347365	-1.18E+01
20371	Foxp3	forkhead box P3	1.00E-05	0.0340109	-1.17E+01
20391	Sgca	sarcoglycan, alpha (dystrophin-associated glycoprotein)	1.00E-05	0.226176	-1.45E+01
20431	Pmel	premelanosome protein	0.114517	0.230257	-1.01E+00
20459	Ptk6	PTK6 protein tyrosine kinase 6	1.00E-05	0.0525601	-1.24E+01
20460	Stil	Scl/Tal1 interrupting locus	0.204633	0.0875645	1.22E+00
20495	Slc12a1	solute carrier family 12, member 1	1.00E-05	0.0759461	-1.29E+01
20521	Slc22a12	solute carrier family 22 (organic anion/cation transporter), member 12	0.119105	0.35596	-1.58E+00
20533	Slc4a1	solute carrier family 4 (anion exchanger), member 1	1.00E-05	0.0841129	-1.30E+01
20558	Slfn4	schlafen 4	1.00E-05	0.0586336	-1.25E+01
20612	Siglec1	sialic acid binding Ig-like lectin 1, sialoadhesin	0.040364	0.0198536	1.02E+00
20613	Snai1	snail homolog 1 (Drosophila)	0.596256	0.227381	1.39E+00
20671	Sox17	SRY-box containing gene 17	0.889988	1.80158	-1.02E+00
20700	Serpina1a	serine (or cysteine) peptidase inhibitor, clade A, member 1A	0.203649	0.0710587	1.52E+00
20717	Serpina3m	serine (or cysteine) peptidase inhibitor, clade A, member 3M	2.32933	1.04483	1.16E+00
20737	Spn	sialophorin	1.00E-05	0.0882907	-1.31E+01
20753	Sprr1a	small proline-rich protein 1A	0.649541	1.00E-05	1.60E+01
20755	Sprr2a1	small proline-rich protein 2A1	0.063928	1.00E-05	1.26E+01
20871	Aurkc	aurora kinase C	1.00E-05	0.103107	-1.33E+01
20878	Aurka	aurora kinase A	0.544725	0.251873	1.11E+00
20997	T	brachyury	1.00E-05	0.0613531	-1.26E+01
21337	Tacr2	tachykinin receptor 2	0.230689	1.00E-05	1.45E+01
21350	Tal2	T cell acute lymphocytic leukemia 2	1.00E-05	0.219019	-1.44E+01
21380	Tbx1	T-box 1	0.53054	1.47866	-1.48E+00

21384	Tbx15	T-box 15	0.452463	0.222984	1.02E+00
21387	Tbx4	T-box 4	1.00E-05	0.0435135	-1.21E+01
21401	Tcea3	transcription elongation factor A (SII), 3	1.00E-05	0.388026	-1.52E+01
21405	Hnf1a	HNF1 homeobox A	1.00E-05	0.0422382	-1.20E+01
21407	Tcf15	transcription factor 15	0.464657	0.220528	1.08E+00
21418	Tfap2a	transcription factor AP-2, alpha	1.00E-05	0.0526001	-1.24E+01
21420	Tfap2c	transcription factor AP-2, gamma	0.249452	0.0517802	2.27E+00
21645	Tcte1	t-complex-associated testis expressed 1	0.930908	0.445874	1.06E+00
21647	Tcte3	t-complex-associated testis expressed 3	1.00E-05	0.234002	-1.45E+01
21667	Tdgf1	teratocarcinoma-derived growth factor 1	1.00E-05	0.252089	-1.46E+01
21679	Tead4	TEA domain family member 4	0.0874856	0.222174	-1.34E+00
21684	Tectb	tectorin beta	0.0428331	0.180371	-2.07E+00
21789	Tfpi2	tissue factor pathway inhibitor 2	1.00E-05	0.0894266	-1.31E+01
21816	Tgm1	transglutaminase 1, K polypeptide	1.00E-05	0.0439316	-1.21E+01
21819	Tg	thyroglobulin	1.00E-05	0.0132097	-1.04E+01
21822	Tgtp1	T cell specific GTPase 1	2.64155	1.31435	1.01E+00
21830	Theg	testicular haploid expressed gene	0.103639	0.214013	-1.05E+00
21877	Tk1	thymidine kinase 1	0.109382	0.325718	-1.57E+00
21897	Tlr1	toll-like receptor 1	0.630621	0.279002	1.18E+00
21909	Tlx2	T cell leukemia, homeobox 2	0.354289	0.163169	1.12E+00
21935	Tnfrsf17	tumor necrosis factor receptor superfamily, member 17	1.00E-05	0.191905	-1.42E+01
21936	Tnfrsf18	tumor necrosis factor receptor superfamily, member 18	0.293495	0.910662	-1.63E+00
21957	Tnnt3	troponin T3, skeletal, fast	0.100062	0.220845	-1.14E+00
21990	Tph1	tryptophan hydroxylase 1	0.0477895	0.230144	-2.27E+00
22029	Traf1	TNF receptor-associated factor 1	0.189156	0.536988	-1.51E+00

22097	Tsix	X (inactive)-specific transcript, antisense	0.0502443	1.00E-05	1.23E+01
22116	Tsks	testis-specific serine kinase substrate	0.60325	0.292131	1.05E+00
22163	Tnfrsf4	tumor necrosis factor receptor superfamily, member 4	0.240244	0.119058	1.01E+00
22202	Uba1y	ubiquitin-activating enzyme, Chr Y	0.0279887	0.0871026	-1.64E+00
22226	Ucn	urocortin	0.391656	1.10801	-1.50E+00
22268	Upk1b	uroplakin 1B	0.765177	0.366382	1.06E+00
22270	Upk3a	uroplakin 3A	1.00E-05	0.128857	-1.37E+01
22271	Upp1	uridine phosphorylase 1	1.28985	2.63227	-1.03E+00
22296	Vmn1r51	vomeronasal 1 receptor 51	1.00E-05	0.0500157	-1.23E+01
22362	Vpreb1	pre-B lymphocyte gene 1	1.00E-05	0.190656	-1.42E+01
22403	Wisp2	WNT1 inducible signaling pathway protein 2	0.248213	1.00E-05	1.46E+01
22408	Wnt1	wingless-related MMTV integration site 1	0.249715	0.0784391	1.67E+00
22410	Wnt10b	wingless related MMTV integration site 10b	1.00E-05	0.211455	-1.44E+01
22412	Wnt9b	wingless-type MMTV integration site 9B	1.24519	0.547009	1.19E+00
22431	Wt1	Wilms tumor 1 homolog	0.0449324	0.0933071	-1.05E+00
22637	Zap70	zeta-chain (TCR) associated protein kinase	0.657922	1.62247	-1.30E+00
22787	Zp2	zona pellucida glycoprotein 2	1.00E-05	0.0521279	-1.23E+01
23919	Insl5	insulin-like 5	0.20501	0.42347	-1.05E+00
23920	Insrr	insulin receptor-related receptor	0.202303	0.0755548	1.42E+00
23923	Aadat	aminoadipate aminotransferase	1.00E-05	0.138015	-1.38E+01
23958	Nr2e3	nuclear receptor subfamily 2, group E, member 3	1.00E-05	0.0628641	-1.26E+01
23961	Oas1b	2'-5' oligoadenylate synthetase 1B	0.0685231	0.355002	-2.37E+00
24013	Grk1	G protein-coupled receptor kinase 1	0.109692	0.0440357	1.32E+00
24046	Scn11a	sodium channel, voltage-gated, type XI, alpha	0.182128	0.0429261	2.09E+00
24058	Sigirr	single immunoglobulin and toll-interleukin 1 receptor (TIR)	0.215346	0.496658	-1.21E+00

		domain			
24084	Tekt2	tektin 2	0.768164	0.316391	1.28E+00
24087	Tll2	tolloid-like 2	0.171109	0.0694284	1.30E+00
24113	Vax2	ventral anterior homeobox containing gene 2	1.00E-05	0.129682	-1.37E+01
26358	Aldh1a7	aldehyde dehydrogenase family 1, subfamily A7	0.309658	0.780261	-1.33E+00
26361	Avpr1b	arginine vasopressin receptor 1B	1.00E-05	0.0284346	-1.15E+01
26365	Ceacam1	carcinoembryonic antigen-related cell adhesion molecule 1	0.556138	1.69039	-1.60E+00
26366	Ceacam10	carcinoembryonic antigen-related cell adhesion molecule 10	1.00E-05	0.135231	-1.37E+01
26368	Ceacam9	carcinoembryonic antigen-related cell adhesion molecule 9	0.255106	1.00E-05	1.46E+01
26423	Nr5a1	nuclear receptor subfamily 5, group A, member 1	0.0922413	0.229963	-1.32E+00
26561	Mmp23	matrix metallopeptidase 23	0.324724	1.06734	-1.72E+00
26888	Clec4a2	C-type lectin domain family 4, member a2	0.522114	0.0983232	2.41E+00
26918	Ern2	endoplasmic reticulum (ER) to nucleus signalling 2	0.295126	0.126251	1.23E+00
26927	Foxl2	forkhead box L2	0.162004	0.713302	-2.14E+00
26944	Tinag	tubulointerstitial nephritis antigen	1.00E-05	0.0655203	-1.27E+01
26945	Tpsg1	tryptase gamma 1	1.00E-05	0.126738	-1.36E+01
26971	Pla2g2f	phospholipase A2, group IIF	0.192655	0.0491119	1.97E+00
27007	Klrk1	killer cell lectin-like receptor subfamily K, member 1	1.00E-05	0.0339944	-1.17E+01
27028	Ermap	erythroblast membrane-associated protein	0.0476141	0.152174	-1.68E+00
27052	Aoah	acyloxyacyl hydrolase	1.00E-05	0.151756	-1.39E+01
27083	Xlr4b	X-linked lymphocyte-regulated 4B	1.00E-05	0.172157	-1.41E+01
27140	Tlx3	T cell leukemia, homeobox 3	1.00E-05	0.160559	-1.40E+01
27218	Slamf1	signaling lymphocytic activation molecule family member 1	0.113381	1.00E-05	1.35E+01
27222	Atp1a4	ATPase, Na+/K+ transporting, alpha 4 polypeptide	0.0671954	1.00E-05	1.27E+01
27356	Insl6	insulin-like 6	0.226065	0.481539	-1.09E+00

27389	Dusp13	dual specificity phosphatase 13	1.00E-05	0.0797336	-1.30E+01
27390	Mmel1	membrane metallo-endopeptidase-like 1	0.310044	0.6734	-1.12E+00
29818	Hspb7	heat shock protein family, member 7 (cardiovascular)	0.306306	0.134261	1.19E+00
29857	Mapk12	mitogen-activated protein kinase 12	1.30847	2.86007	-1.13E+00
30044	Opn4	opsin 4 (melanopsin)	0.0673643	0.341149	-2.34E+00
30924	Angptl3	angiopoietin-like 3	0.0524472	0.117013	-1.16E+00
30943	Prss30	protease, serine, 30	0.092662	0.373617	-2.01E+00
50528	Tmprss2	transmembrane protease, serine 2	1.00E-05	0.0760097	-1.29E+01
50540	Igbp1b	immunoglobulin (CD79A) binding protein 1b	0.0806264	0.338884	-2.07E+00
50701	Elane	elastase, neutrophil expressed	1.00E-05	0.175862	-1.41E+01
50764	Fbxo15	F-box protein 15	1.00E-05	0.226174	-1.45E+01
50778	Rgs1	regulator of G-protein signaling 1	0.366936	1.00E-05	1.52E+01
51795	Srpx	sushi-repeat-containing protein	0.144498	0.0512865	1.49E+00
52033	Pbk	PDZ binding kinase	0.210652	0.464191	-1.14E+00
52276	Cdca8	cell division cycle associated 8	0.355871	0.751443	-1.08E+00
52285	D6Ertd474e	DNA segment, Chr 6, ERATO Doi 474, expressed	0.166921	1.00E-05	1.40E+01
52331	Stbd1	starch binding domain 1	2.78687	1.3449	1.05E+00
52570	Ccdc69	coiled-coil domain containing 69	0.0771462	0.161457	-1.07E+00
52685	Cd300lg	CD300 antigen like family member G	0.0537993	0.112703	-1.07E+00
53404	Atoh7	atonal homolog 7 (Drosophila)	1.87059	1.00E-05	1.75E+01
53603	Tslp	thymic stromal lymphopoietin	0.330987	1.00E-05	1.50E+01
53791	Tlr5	toll-like receptor 5	0.0390308	0.122695	-1.65E+00
54368	Gp9	glycoprotein 9 (platelet)	0.204666	0.582953	-1.51E+00
54378	Cacng6	calcium channel, voltage-dependent, gamma subunit 6	0.894582	1.00E-05	1.64E+01
54426	Hgfac	hepatocyte growth factor activator	1.00E-05	0.129374	-1.37E+01

54427	Dnmt3l	DNA (cytosine-5-)-methyltransferase 3-like	1.00E-05	0.124853	-1.36E+01
55938	Apom	apolipoprotein M	0.200855	0.627637	-1.64E+00
55993	Msh4	mutS homolog 4 (E. coli)	1.00E-05	0.0351541	-1.18E+01
56066	Cxcl11	chemokine (C-X-C motif) ligand 11	1.00E-05	0.0829727	-1.30E+01
56173	Cldn14	claudin 14	0.115065	0.43238	-1.91E+00
56190	Rbm38	RNA binding motif protein 38	0.484798	1.55995	-1.69E+00
56221	Ccl24	chemokine (C-C motif) ligand 24	0.189432	1.00E-05	1.42E+01
56318	Acpp	acid phosphatase, prostate	0.173389	0.079811	1.12E+00
56429	Dpt	dermatopontin	1.00E-05	0.0719796	-1.28E+01
56460	Pkp3	plakophilin 3	0.155689	0.0501795	1.63E+00
56522	Papolb	poly (A) polymerase beta (testis specific)	1.00E-05	0.0483234	-1.22E+01
56532	Ripk3	receptor-interacting serine-threonine kinase 3	0.196775	0.0663293	1.57E+00
56546	Sec1	secretory blood group 1	0.253528	0.121134	1.07E+00
56620	Clec4n	C-type lectin domain family 4, member n	1.00E-05	0.550765	-1.57E+01
56629	Dnase2b	deoxyribonuclease II beta	1.00E-05	0.0528018	-1.24E+01
56696	Gpr132	G protein-coupled receptor 132	1.00E-05	0.0581306	-1.25E+01
56734	Tulp2	tubby-like protein 2	1.00E-05	0.0680381	-1.27E+01
56760	Clec1b	C-type lectin domain family 1, member b	0.0955893	0.263486	-1.46E+00
56774	Slc6a14	solute carrier family 6 (neurotransmitter transporter), member 14	1.00E-05	0.0348406	-1.18E+01
56792	Stap1	signal transducing adaptor family member 1	0.270314	0.0988825	1.45E+00
56838	Ccl28	chemokine (C-C motif) ligand 28	0.343299	1.00E-05	1.51E+01
56843	Trpm5	transient receptor potential cation channel, subfamily M, member 5	0.0546076	1.00E-05	1.24E+01
57262	Retnla	resistin like alpha	1.00E-05	0.269003	-1.47E+01

57264	Retn	resistin	1.00E-05	0.117645	-1.35E+01
57269	Olfr1507	olfactory receptor 1507	0.323321	1.00E-05	1.50E+01
57294	Rps27	ribosomal protein S27	137.02	6.77406	4.34E+00
57349	Ppbp	pro-platelet basic protein	1.00E-05	0.437276	-1.54E+01
57442	Kcne3	potassium voltage-gated channel, Isk-related subfamily, gene 3	1.00E-05	0.16498	-1.40E+01
57444	Isg20	interferon-stimulated protein	0.782554	0.156281	2.32E+00
57746	Piwil2	piwi-like RNA-mediated gene silencing 2	0.0892669	0.0233927	1.93E+00
57749	Piwil1	piwi-like RNA-mediated gene silencing 1	0.028736	0.0599852	-1.06E+00
57756	Fhl5	four and a half LIM domains 5	0.122827	0.259771	-1.08E+00
57814	Kcne4	potassium voltage-gated channel, Isk-related subfamily, gene 4	0.876101	0.421895	1.05E+00
57890	Il17re	interleukin 17 receptor E	0.416479	0.154833	1.43E+00
57911	Gsdma	gasdermin A	0.0746011	1.00E-05	1.29E+01
58182	Prokr1	prokineticin receptor 1	1.00E-05	0.0295595	-1.15E+01
58203	Zbp1	Z-DNA binding protein 1	1.00E-05	0.0544741	-1.24E+01
58206	Zbtb32	zinc finger and BTB domain containing 32	0.147839	0.455785	-1.62E+00
58207	Slc43a3	solute carrier family 43, member 3	0.664553	0.263052	1.34E+00
58223	Mmp19	matrix metallopeptidase 19	0.102204	0.213479	-1.06E+00
58251	BC100451	cDNA sequence BC100451	0.153938	1.00E-05	1.39E+01
58865	Tdh	L-threonine dehydrogenase	1.00E-05	0.0679935	-1.27E+01
58866	Treh	trehalase (brush-border membrane glycoprotein)	1.00E-05	0.060355	-1.26E+01
58916	Myot	myotilin	1.00E-05	0.0519108	-1.23E+01
59011	Myoz1	myozenin 1	1.00E-05	0.0942836	-1.32E+01
60505	Il21	interleukin 21	0.068342	1.00E-05	1.27E+01

60531	Npvf	neuropeptide VF precursor	1.00E-05	0.520292	-1.57E+01
63857	Bcmo1	beta-carotene 15,15'-monooxygenase	0.121573	1.00E-05	1.36E+01
63859	Impg1	interphotoreceptor matrix proteoglycan 1	0.0549908	0.148396	-1.43E+00
64095	Gpr35	G protein-coupled receptor 35	0.0289493	0.0907906	-1.65E+00
64214	Rgs18	regulator of G-protein signaling 18	0.106439	1.00E-05	1.34E+01
64406	Sp5	trans-acting transcription factor 5	0.306339	0.780753	-1.35E+00
64454	Slc5a4b	solute carrier family 5 (neutral amino acid transporters, system A), member 4b	1.00E-05	0.137303	-1.37E+01
64705	Dpys	dihydropyrimidinase	0.160801	1.00E-05	1.40E+01
64918	Bhmt2	betaine-homocysteine methyltransferase 2	0.277538	0.114813	1.27E+00
65086	Lpar3	lysophosphatidic acid receptor 3	0.118293	0.244225	-1.05E+00
66106	Smpx	small muscle protein, X-linked	0.223717	0.110615	1.02E+00
66123	1110006024Rik	RIKEN cDNA 1110006024 gene	1.14265	0.556437	1.04E+00
66166	S100a14	S100 calcium binding protein A14	1.00E-05	0.272536	-1.47E+01
66338	Cdrt4	CMT1A duplicated region transcript 4	1.00E-05	0.173344	-1.41E+01
66442	Spc25	SPC25, NDC80 kinetochore complex component, homolog (S. cerevisiae)	0.39482	0.192405	1.04E+00
66451	2610528J11Rik	RIKEN cDNA 2610528J11 gene	0.544222	1.00E-05	1.57E+01
66561	2310042E22Rik	RIKEN cDNA 2310042E22 gene	0.548425	0.226438	1.28E+00
66605	1700017N19Rik	RIKEN cDNA 1700017N19 gene	1.00E-05	0.124123	-1.36E+01
66654	Tex12	testis expressed gene 12	1.00E-05	0.453584	-1.55E+01
66696	Snx31	sorting nexin 31	0.0820922	0.172964	-1.08E+00
66707	Nkapl	NFKB activating protein-like	0.274726	0.0760434	1.85E+00
66720	Klhl10	kelch-like 10	0.0568428	0.296459	-2.38E+00
66729	Ankrd61	ankyrin repeat domain 61	0.400452	0.0830068	2.27E+00

66732	4921530L21Rik	RIKEN cDNA 4921530L21 gene	0.0854423	0.186646	-1.13E+00
66766	Tmem239	transmembrane 239	0.249455	1.00E-05	1.46E+01
66779	4933432I09Rik	RIKEN cDNA 4933432I09 gene	0.544929	0.220744	1.30E+00
66809	Krt20	keratin 20	0.0515989	0.385977	-2.90E+00
66813	Bcl2l14	BCL2-like 14 (apoptosis facilitator)	1.00E-05	0.211204	-1.44E+01
66857	Plbd1	phospholipase B domain containing 1	1.00E-05	0.119354	-1.35E+01
66931	1700010I14Rik	RIKEN cDNA 1700010I14 gene	0.355559	0.165169	1.11E+00
67038	2010109I03Rik	RIKEN cDNA 2010109I03 gene	1.00E-05	0.063949	-1.26E+01
67052	Ndc80	NDC80 homolog, kinetochore complex component (S. cerevisiae)	0.202162	0.0897896	1.17E+00
67112	Fgf22	fibroblast growth factor 22	1.00E-05	0.920773	-1.65E+01
67133	Gp2	glycoprotein 2 (zymogen granule membrane)	0.0548275	0.115731	-1.08E+00
67194	2700038G22Rik	RIKEN cDNA 2700038G22 gene	0.394104	0.816869	-1.05E+00
67317	1700022I11Rik	RIKEN cDNA 1700022I11 gene	1.00E-05	0.0303068	-1.16E+01
67331	Atp8b3	ATPase, class I, type 8B, member 3	1.00E-05	0.0546125	-1.24E+01
67394	4930404I05Rik	RIKEN cDNA 4930404I05 gene	0.134317	0.276917	-1.04E+00
67523	1700094J05Rik	RIKEN cDNA 1700094J05 gene	1.00E-05	0.245543	-1.46E+01
67531	5730408K05Rik	RIKEN cDNA 5730408K05 gene	3.27625	0.67442	2.28E+00
67578	Patl2	protein associated with topoisomerase II homolog 2 (yeast)	0.0969978	0.304784	-1.65E+00
67592	4930524B15Rik	RIKEN cDNA 4930524B15 gene	1.00E-05	0.134423	-1.37E+01
67593	4930519G04Rik	RIKEN cDNA 4930519G04 gene	1.00E-05	0.185419	-1.42E+01
67638	4930483J18Rik	RIKEN cDNA 4930483J18 gene	1.00E-05	0.104641	-1.34E+01
67642	4930515G01Rik	RIKEN cDNA 4930515G01 gene	1.00E-05	0.257319	-1.47E+01
67701	Wfdc2	WAP four-disulfide core domain 2	2.97224	0.851569	1.80E+00
67715	2010106E10Rik	RIKEN cDNA 2010106E10 gene	1.00E-05	0.0977176	-1.33E+01
67745	4930583K01Rik	RIKEN cDNA 4930583K01 gene	1.00E-05	0.149237	-1.39E+01

67746	4930577N17Rik	RIKEN cDNA 4930577N17 gene	0.339397	1.13648	-1.74E+00
67749	Mgarp	mitochondria localized glutamic acid rich protein	1.00E-05	0.102362	-1.33E+01
67765	5830432E09Rik	RIKEN cDNA 5830432E09 gene	1.00E-05	0.0834735	-1.30E+01
68026	2810417H13Rik	RIKEN cDNA 2810417H13 gene	0.09871	0.218936	-1.15E+00
68054	Serpina12	serine (or cysteine) peptidase inhibitor, clade A (alpha-1 antiproteinase, antitrypsin), member 12	1.00E-05	0.0634495	-1.26E+01
68144	5031426D15Rik	RIKEN cDNA 5031426D15 gene	0.0366686	0.0812593	-1.15E+00
68222	Fam166a	family with sequence similarity 166, member A	1.00E-05	0.214453	-1.44E+01
68232	1700120K04Rik	RIKEN cDNA 1700120K04 gene	1.00E-05	0.362428	-1.51E+01
68233	Fam229a	family with sequence similarity 229, member A	1.30691	1.00E-05	1.70E+01
68265	Iqcf3	IQ motif containing F3	1.00E-05	0.101744	-1.33E+01
68270	Dnaaf1	dynein, axonemal assembly factor 1	0.0395245	0.160633	-2.02E+00
68306	4930565N06Rik	RIKEN cDNA 4930565N06 gene	1.00E-05	0.0855291	-1.31E+01
68311	Lypd2	Ly6/Plaur domain containing 2	0.804413	1.00E-05	1.63E+01
68352	Aspdh	aspartate dehydrogenase domain containing	1.00E-05	0.158582	-1.40E+01
68393	Mogat1	monoacylglycerol O-acyltransferase 1	0.815406	0.240503	1.76E+00
68436	Rpl34	ribosomal protein L34	11.9762	5.33589	1.17E+00
68487	Tmem140	transmembrane protein 140	1.00E-05	0.100971	-1.33E+01
68509	Ptx4	pentraxin 4	1.00E-05	0.154034	-1.39E+01
68527	Ucma	upper zone of growth plate and cartilage matrix associated	0.758866	0.181825	2.06E+00
68553	Col6a4	collagen, type VI, alpha 4	0.043623	0.106035	-1.28E+00
68612	Ube2c	ubiquitin-conjugating enzyme E2C	0.510401	1.00E-05	1.56E+01
68632	Myct1	myc target 1	0.119551	0.438333	-1.87E+00
68723	Hrnr	hornerin	1.00E-05	0.0100608	-9.97E+00
68764	Cdhr3	cadherin-related family member 3	0.384938	0.844088	-1.13E+00

68774	Ms4a6d	membrane-spanning 4-domains, subfamily A, member 6D	1.85729	0.783123	1.25E+00
68790	Fendrr	Foxf1 adjacent non-coding developmental regulatory RNA	0.1151	0.23268	-1.02E+00
68800	1110059M19Rik	RIKEN cDNA 1110059M19 gene	1.56039	0.7199	1.12E+00
68888	Gkn3	gastrokine 3	3.26626	1.27796	1.35E+00
68954	1500012K07Rik	RIKEN cDNA 1500012K07 gene	0.894322	0.357	1.32E+00
69032	Lyzl4	lysozyme-like 4	1.00E-05	0.295171	-1.48E+01
69047	Atp2c2	ATPase, Ca++ transporting, type 2C, member 2	0.120412	0.441972	-1.88E+00
69069	1810011H11Rik	RIKEN cDNA 1810011H11 gene	0.225231	0.554235	-1.30E+00
69073	1810019J16Rik	RIKEN cDNA 1810019J16 gene	0.0969011	0.276525	-1.51E+00
69083	Sult1c2	sulfotransferase family, cytosolic, 1C, member 2	1.00E-05	0.0716867	-1.28E+01
69121	Chrdl2	chordin-like 2	0.0851876	0.172891	-1.02E+00
69134	Fam25c	family with sequence similarity 25, member C	1.00E-05	0.637777	-1.60E+01
69142	Cd209f	CD209f antigen	0.238026	0.610806	-1.36E+00
69165	Cd209b	CD209b antigen	0.129775	0.280972	-1.11E+00
69187	Erp27	endoplasmic reticulum protein 27	1.00E-05	0.140235	-1.38E+01
69248	2610035F20Rik	RIKEN cDNA 2610035F20 gene	0.124131	0.25771	-1.05E+00
69301	Tescl	tescalcin-like	1.00E-05	0.19392	-1.42E+01
69306	Efcab9	EF-hand calcium binding domain 9	1.00E-05	0.550863	-1.57E+01
69319	1700001K23Rik	RIKEN cDNA 1700001K23 gene	1.00E-05	0.370603	-1.52E+01
69325	1700012B09Rik	RIKEN cDNA 1700012B09 gene	1.43304	0.554833	1.37E+00
69355	1700010J16Rik	RIKEN cDNA 1700010J16 gene	1.00E-05	0.131485	-1.37E+01
69376	Zpbp2	zona pellucida binding protein 2	1.00E-05	0.187779	-1.42E+01
69379	C8g	complement component 8, gamma polypeptide	0.187509	1.16855	-2.64E+00
69386	Hist1h4h	histone cluster 1, H4h	1.00E-05	4.08522	-1.86E+01
69387	Dnajb13	DnaJ (Hsp40) related, subfamily B, member 13	0.39136	0.842468	-1.11E+00

69397	1700019A02Rik	RIKEN cDNA 1700019A02 gene	1.00E-05	0.206458	-1.43E+01
69412	1700016L04Rik	RIKEN cDNA 1700016L04 gene	1.00E-05	0.148527	-1.39E+01
69428	1700016C15Rik	RIKEN cDNA 1700016C15 gene	1.00E-05	0.185182	-1.42E+01
69443	1700027J07Rik	RIKEN cDNA 1700027J07 gene	1.00E-05	0.117099	-1.35E+01
69457	2310005G13Rik	RIKEN cDNA 2310005G13 gene	1.00E-05	0.117292	-1.35E+01
69481	Actl9	actin-like 9	0.291177	0.0987773	1.56E+00
69496	Dydc1	DPY30 domain containing 1	0.228069	1.00E-05	1.45E+01
69540	Klk10	kallikrein related-peptidase 10	0.223503	1.00E-05	1.44E+01
69543	Capns2	calpain, small subunit 2	1.00E-05	0.141502	-1.38E+01
69553	Fam65c	family with sequence similarity 65, member C	0.0488778	0.190192	-1.96E+00
69563	2310015B20Rik	RIKEN cDNA 2310015B20 gene	1.00E-05	0.808528	-1.63E+01
69629	2310039L15Rik	RIKEN cDNA 2310039L15 gene	0.679725	0.241138	1.50E+00
69631	2310014F07Rik	RIKEN cDNA 2310014F07 gene	1.00E-05	0.10509	-1.34E+01
69640	Fam83g	family with sequence similarity 83, member G	1.00E-05	0.0505313	-1.23E+01
69671	Tmem52	transmembrane protein 52	0.543416	1.00E-05	1.57E+01
69681	Cdk3-ps	cyclin-dependent kinase 3, pseudogene	0.367455	0.742805	-1.02E+00
69739	2410004I01Rik	RIKEN cDNA 2410004I01 gene	1.00E-05	0.899304	-1.65E+01
69750	2410021H03Rik	RIKEN cDNA 2410021H03 gene	1.00E-05	0.519978	-1.57E+01
69774	Ms4a6b	membrane-spanning 4-domains, subfamily A, member 6B	0.81815	0.292603	1.48E+00
69797	1600029I14Rik	RIKEN cDNA 1600029I14 gene	1.00E-05	0.537256	-1.57E+01
69798	1810044D09Rik	RIKEN cDNA 1810044D09 gene	2.31615	1.00E-05	1.78E+01
69810	Clec4b1	C-type lectin domain family 4, member b1	1.00E-05	0.453454	-1.55E+01
69815	Krtcap3	keratinocyte associated protein 3	0.216409	0.622722	-1.52E+00
69824	Glod5	glyoxalase domain containing 5	0.359021	1.00E-05	1.51E+01
69885	Clhc1	clathrin heavy chain linker domain containing 1	1.00E-05	0.284341	-1.48E+01

69909	2610027K06Rik	RIKEN cDNA 2610027K06 gene	1.05935	0.429553	1.30E+00
69941	2810408I11Rik	RIKEN cDNA 2810408I11 gene	0.208035	1.54473	-2.89E+00
69964	2810403D21Rik	RIKEN cDNA 2810403D21 gene	0.146958	0.477717	-1.70E+00
69966	2810404M03Rik	RIKEN cDNA 2810404M03 gene	0.0916823	0.201963	-1.14E+00
69994	Rsc1a1	regulatory solute carrier protein, family 1, member 1	0.39639	0.795866	-1.01E+00
70004	1700028J19Rik	RIKEN cDNA 1700028J19 gene	0.134022	0.549078	-2.03E+00
70024	Mcm10	minichromosome maintenance deficient 10 (S. cerevisiae)	0.174741	0.0621078	1.49E+00
70040	2610037D02Rik	RIKEN cDNA 2610037D02 gene	1.00E-05	0.102231	-1.33E+01
70045	2610528A11Rik	RIKEN cDNA 2610528A11 gene	0.189471	0.59273	-1.65E+00
70054	Ccdc89	coiled-coil domain containing 89	0.738987	0.249486	1.57E+00
70061	Sdr9c7	4short chain dehydrogenase/reductase family 9C, member 7	1.00E-05	0.0407027	-1.20E+01
70080	Igsf23	immunoglobulin superfamily, member 23	0.205701	1.00E-05	1.43E+01
70129	Slc44a4	solute carrier family 44, member 4	0.124679	0.253862	-1.03E+00
70162	2210417K05Rik	RIKEN cDNA 2210417K05 gene	0.311614	1.55033	-2.31E+00
70163	Lypd8	LY6/PLAUR domain containing 8	1.00E-05	0.133468	-1.37E+01
70218	Kif18b	kinesin family member 18B	0.114638	1.00E-05	1.35E+01
70281	2310068J16Rik	RIKEN cDNA 2310068J16 gene	1.00E-05	0.39141	-1.53E+01
70419	2810408A11Rik	RIKEN cDNA 2810408A11 gene	0.0708436	0.361111	-2.35E+00
70487	5730403I07Rik	RIKEN cDNA 5730403I07 gene	0.0628922	0.136661	-1.12E+00
70489	5730405O15Rik	RIKEN cDNA 5730405O15 gene	0.140599	0.598243	-2.09E+00
70592	5730480H06Rik	RIKEN cDNA 5730480H06 gene	0.626602	0.305812	1.03E+00
70626	5730522E02Rik	RIKEN cDNA 5730522E02 gene	0.735646	0.240077	1.62E+00
70691	3830403N18Rik	RIKEN cDNA 3830403N18 gene	1.00E-05	0.0991899	-1.33E+01
70730	6330409D20Rik	RIKEN cDNA 6330409D20 gene	1.00174	2.12213	-1.08E+00
70753	6330415B21Rik	RIKEN cDNA 6330415B21 gene	1.00E-05	0.0583586	-1.25E+01

70789	Kynu	kynureninase (L-kynurenine hydrolase)	1.00E-05	0.0939024	-1.32E+01
70894	Efcab3	EF-hand calcium binding domain 3	1.00E-05	0.0674996	-1.27E+01
70902	Lpcat2b	lysophosphatidylcholine acyltransferase 2B	1.00E-05	0.042615	-1.21E+01
70928	Trim69	tripartite motif-containing 69	1.00E-05	0.0662238	-1.27E+01
70957	4921530L18Rik	RIKEN cDNA 4921530L18 gene	0.698419	1.00E-05	1.61E+01
70971	4931429P17Rik	RIKEN cDNA 4931429P17 gene	1.00E-05	0.0575757	-1.25E+01
70976	Ccdc105	coiled-coil domain containing 105	1.00E-05	0.160011	-1.40E+01
70979	Fancd2os	Fancd2 opposite strand	0.0828915	0.173006	-1.06E+00
71000	4931440J10Rik	RIKEN cDNA 4931440J10 gene	0.122626	0.270681	-1.14E+00
71003	Prss41	protease, serine, 41	0.270453	1.00E-05	1.47E+01
71027	Tmem30c	transmembrane protein 30C	1.00E-05	0.0917695	-1.32E+01
71030	4933403O08Rik	RIKEN cDNA 4933403O08 gene	0.135483	1.00E-05	1.37E+01
71045	4933406I18Rik	RIKEN cDNA 4933406I18 gene	0.128223	0.279408	-1.12E+00
71046	4933405L10Rik	RIKEN cDNA 4933405L10 gene	1.00E-05	0.119222	-1.35E+01
71082	4933416M07Rik	RIKEN cDNA 4933416M07 gene	0.417718	1.00E-05	1.54E+01
71133	4933422A05Rik	RIKEN cDNA 4933422A05 gene	1.00E-05	0.0885492	-1.31E+01
71145	Scara5	scavenger receptor class A, member 5 (putative)	0.294855	0.0921107	1.68E+00
71153	4933417O13Rik	RIKEN cDNA 4933417O13 gene	0.0776299	0.245129	-1.66E+00
71166	4933424G06Rik	RIKEN cDNA 4933424G06 gene	1.00E-05	0.341298	-1.51E+01
71186	4933417D19Rik	RIKEN cDNA 4933417D19 gene	0.202107	0.622548	-1.62E+00
71200	Dydc2	DPY30 domain containing 2	0.932499	0.244811	1.93E+00
71226	4933433G19Rik	RIKEN cDNA 4933433G19 gene	0.217743	1.00E-05	1.44E+01
71227	Daw1	dynein assembly factor with WDR repeat domains 1	0.340559	0.698963	-1.04E+00
71236	Rtdr1	rhabdoid tumor deletion region gene 1	0.228754	1.00E-05	1.45E+01
71264	4933432I03Rik	RIKEN cDNA 4933432I03 gene	0.215485	1.00E-05	1.44E+01

71274	4933433G15Rik	RIKEN cDNA 4933433G15 gene	0.216125	0.448277	-1.05E+00
71296	4933436C20Rik	RIKEN cDNA 4933436C20 gene	1.10906	0.494295	1.17E+00
71313	Fsip1	fibrous sheath-interacting protein 1	0.705259	0.191806	1.88E+00
71319	4933439K11Rik	RIKEN cDNA 4933439K11 gene	0.128659	0.404022	-1.65E+00
71326	Treml1	triggering receptor expressed on myeloid cells-like 1	0.215863	1.00E-05	1.44E+01
71405	Fam83c	family with sequence similarity 83, member C	1.00E-05	0.11117	-1.34E+01
71412	Dhrs2	dehydrogenase/reductase member 2	0.210835	1.00E-05	1.44E+01
71581	9130015A21Rik	RIKEN cDNA 9130015A21 gene	1.00E-05	0.156782	-1.39E+01
71601	Ceacam20	carcinoembryonic antigen-related cell adhesion molecule 20	1.00E-05	0.0865016	-1.31E+01
71661	0610005C13Rik	RIKEN cDNA 0610005C13 gene	0.556213	1.00E-05	1.58E+01
71761	Amdhd1	amidohydrolase domain containing 1	0.0476517	0.151467	-1.67E+00
71775	1300017J02Rik	RIKEN cDNA 1300017J02 gene	0.107378	1.00E-05	1.34E+01
71790	Anxa9	annexin A9	0.378264	0.0666242	2.51E+00
71818	3200001D21Rik	RIKEN cDNA 3200001D21 gene	0.0456991	0.145851	-1.67E+00
71819	Kif23	kinesin family member 23	0.443928	0.189895	1.23E+00
71828	Gtf2a1l	general transcription factor IIA, 1-like	0.291403	0.0759456	1.94E+00
71830	Pdilt	protein disulfide isomerase-like, testis expressed	0.096645	1.00E-05	1.32E+01
71840	Tekt4	tektin 4	0.606501	0.172924	1.81E+00
71856	Wfdc3	WAP four-disulfide core domain 3	0.357783	1.08909	-1.61E+00
71864	Fam217a	family with sequence similarity 217, member A	0.556371	0.139127	2.00E+00
71868	1700023E05Rik	RIKEN cDNA 1700023E05 gene	0.0750109	0.235458	-1.65E+00
71869	Serpinb12	serine (or cysteine) peptidase inhibitor, clade B (ovalbumin), member 12	0.188935	1.00E-05	1.42E+01
71874	2310007B03Rik	RIKEN cDNA 2310007B03 gene	1.00E-05	0.0576005	-1.25E+01
71884	Chit1	chitinase 1 (chitotriosidase)	1.00E-05	0.0653863	-1.27E+01

71898	Apol9b	apolipoprotein L 9b	1.00E-05	0.153263	-1.39E+01
71913	Tmem79	transmembrane protein 79	0.802422	0.31023	1.37E+00
71932	Ephx3	epoxide hydrolase 3	0.051569	0.157997	-1.62E+00
71939	Apol6	apolipoprotein L 6	0.200802	0.0629353	1.67E+00
71950	Nanog	Nanog homeobox	0.273144	1.00E-05	1.47E+01
71985	Acad10	acyl-Coenzyme A dehydrogenase family, member 10	0.714414	0.309737	1.21E+00
71988	Esco2	establishment of cohesion 1 homolog 2 (S. cerevisiae)	1.00E-05	0.0758511	-1.29E+01
71995	Erv3	endogenous retroviral sequence 3	0.0375154	0.200713	-2.42E+00
72002	Slc39a5	solute carrier family 39 (metal ion transporter), member 5	0.227619	0.0761662	1.58E+00
72054	Cyp4f18	cytochrome P450, family 4, subfamily f, polypeptide 18	0.260411	0.0864917	1.59E+00
72058	Igsf5	immunoglobulin superfamily, member 5	0.348933	0.157818	1.14E+00
72074	Anks4b	ankyrin repeat and sterile alpha motif domain containing 4B	0.0905028	0.254285	-1.49E+00
72080	Sapcd2	suppressor APC domain containing 2	0.477388	0.217994	1.13E+00
72088	Ush1c	Usher syndrome 1C	0.109481	0.286574	-1.39E+00
72104	2010106C02Rik	RIKEN cDNA 2010106C02 gene	1.00E-05	0.244014	-1.46E+01
72121	Dennd2d	DENN/MADD domain containing 2D	0.0996668	0.300351	-1.59E+00
72224	1700001J11Rik	ring finger protein 19A pseudogene	0.124558	0.0446651	1.48E+00
72236	Tsnaxip1	translin-associated factor X (Tsnax) interacting protein 1	0.0867101	0.234048	-1.43E+00
72240	1600014C23Rik	RIKEN cDNA 1600014C23 gene	1.00E-05	0.387227	-1.52E+01
72267	Lrrc8e	leucine rich repeat containing 8 family, member E	1.00E-05	0.0617088	-1.26E+01
72281	Sh2d4a	SH2 domain containing 4A	1.00E-05	0.0775051	-1.29E+01
72287	Plekhf1	pleckstrin homology domain containing, family F (with FYVE domain) member 1	3.27477	1.39653	1.23E+00
72310	Nkg7	natural killer cell group 7 sequence	1.00E-05	1.14188	-1.68E+01
72401	Slc43a1	solute carrier family 43, member 1	1.00E-05	0.113382	-1.35E+01

72433	Rab38	RAB38, member of RAS oncogene family	0.396612	0.828618	-1.06E+00
72518	2610307P16Rik	RIKEN cDNA 2610307P16 gene	1.00E-05	0.0490323	-1.23E+01
72575	C430049B03Rik	RIKEN cDNA C430049B03 gene	0.147277	0.0440279	1.74E+00
72608	2700069I18Rik	RIKEN cDNA 2700069I18 gene	1.11751	0.395417	1.50E+00
72648	2700081L22Rik	RIKEN cDNA 2700081L22 gene	1.00E-05	0.512997	-1.56E+01
72778	Dnajc22	DnaJ (Hsp40) homolog, subfamily C, member 22	0.187405	1.00E-05	1.42E+01
72789	Veph1	ventricular zone expressed PH domain-containing 1	1.00E-05	0.0169263	-1.07E+01
72807	Zfp429	zinc finger protein 429	0.270315	0.656048	-1.28E+00
73076	3100003L05Rik	RIKEN cDNA 3100003L05 gene	1.00E-05	0.218942	-1.44E+01
73106	Prss57	protease, serine 57	0.236621	0.621197	-1.39E+00
73121	Fam101a	family with sequence similarity 101, member A	0.458161	0.182721	1.33E+00
73144	3110039I08Rik	RIKEN cDNA 3110039I08 gene	0.336621	1.00E-05	1.50E+01
73149	Clec4a3	C-type lectin domain family 4, member a3	0.171661	0.556956	-1.70E+00
73167	Arhgap8	Rho GTPase activating protein 8	0.524283	1.00E-05	1.57E+01
73181	Nfatc4	nuclear factor of activated T cells, cytoplasmic, calcineurin dependent 4	0.53586	0.134277	2.00E+00
73183	5430402O13Rik	RIKEN cDNA 5430402O13 gene	0.277636	0.58737	-1.08E+00
73303	1700040N02Rik	RIKEN cDNA 1700040N02 gene	0.289026	0.103914	1.48E+00
73333	Slc25a31	solute carrier family 25 (mitochondrial carrier; adenine nucleotide translocator), member 31	0.0550465	0.241247	-2.13E+00
73358	1700054M17Rik	RIKEN cDNA 1700054M17 gene	1.00E-05	0.402052	-1.53E+01
73363	1700056E22Rik	RIKEN cDNA 1700056E22 gene	1.00E-05	0.225689	-1.45E+01
73366	1700051A21Rik	RIKEN cDNA 1700051A21 gene	0.112002	0.232218	-1.05E+00
73449	1700066B19Rik	RIKEN cDNA 1700066B19 gene	1.00E-05	0.0993766	-1.33E+01
73452	1700063O14Rik	RIKEN cDNA 1700063O14 gene	1.00E-05	0.377864	-1.52E+01

73458	1700055N04Rik	RIKEN cDNA 1700055N04 gene	1.00E-05	0.0623045	-1.26E+01
73523	Pebp4	phosphatidylethanolamine binding protein 4	1.00E-05	0.221509	-1.44E+01
73553	1700091H14Rik	RIKEN cDNA 1700091H14 gene	0.0737892	0.160264	-1.12E+00
73558	1700110K17Rik	RIKEN cDNA 1700110K17 gene	0.345722	1.00E-05	1.51E+01
73600	1700120C14Rik	RIKEN cDNA 1700120C14 gene	1.78967	0.628256	1.51E+00
73660	Cabp4	calcium binding protein 4	0.0871338	0.177309	-1.02E+00
73671	Sult6b1	sulfotransferase family, cytosolic, 6B, member 1	0.0517319	0.113402	-1.13E+00
73673	2410076I21Rik	RIKEN cDNA 2410076I21 gene	0.157425	0.480967	-1.61E+00
73815	4930404H11Rik	RIKEN cDNA 4930404H11 gene	1.00E-05	0.154211	-1.39E+01
73862	4930415F15Rik	RIKEN cDNA 4930415F15 gene	0.197374	0.61788	-1.65E+00
73887	4930417O22Rik	RIKEN cDNA 4930417O22 gene	1.00E-05	0.106365	-1.34E+01
73902	Psmb11	proteasome (prosome, macropain) subunit, beta type, 11	0.0540917	1.00E-05	1.24E+01
73904	4833412C05Rik	RIKEN cDNA 4833412C05 gene	0.124535	0.777758	-2.64E+00
73915	4833419F23Rik	RIKEN cDNA 4833419F23 gene	1.00E-05	0.160223	-1.40E+01
73924	4930401O12Rik	RIKEN cDNA 4930401O12 gene	1.00E-05	0.144076	-1.38E+01
73934	4930412D23Rik	RIKEN cDNA 4930412D23 gene	1.00E-05	0.1888	-1.42E+01
73936	Ccdc175	coiled-coil domain containing 175	0.0932545	0.0355778	1.39E+00
73989	4930452G13Rik	RIKEN cDNA 4930452G13 gene	0.513185	0.224385	1.19E+00
74016	Phf19	PHD finger protein 19	0.0928836	0.254963	-1.46E+00
74041	4632434I11Rik	RIKEN cDNA 4632434I11 gene	0.112992	0.249922	-1.15E+00
74050	4921525O09Rik	RIKEN cDNA 4921525O09 gene	0.21662	0.0756343	1.52E+00
74071	Ifltd1	intermediate filament tail domain containing 1	0.28778	0.762282	-1.41E+00
74072	4933407I05Rik	RIKEN cDNA 4933407I05 gene	1.00E-05	0.66307	-1.60E+01
74075	Syce1	synaptonemal complex central element protein 1	0.0683439	0.440761	-2.69E+00
74107	Cep55	centrosomal protein 55	0.209413	0.0928623	1.17E+00

74121	Acoxl	acyl-Coenzyme A oxidase-like	0.0391248	0.0806985	-1.04E+00
74152	1300002K09Rik	RIKEN cDNA 1300002K09 gene	1.00E-05	0.0706528	-1.28E+01
74176	Tgm5	transglutaminase 5	1.00E-05	0.0483653	-1.22E+01
74184	2310065F04Rik	RIKEN cDNA 2310065F04 gene	0.0799382	1.00E-05	1.30E+01
74220	1700009C05Rik	RIKEN cDNA 1700009C05 gene	1.00E-05	0.183203	-1.42E+01
74340	Ahcyl2	S-adenosylhomocysteine hydrolase-like 2	1.00E-05	40.6565	-2.20E+01
74362	Spag17	sperm associated antigen 17	0.0757974	0.215552	-1.51E+00
74364	4931431C16Rik	RIKEN cDNA 4931431C16 gene	0.231529	0.0479861	2.27E+00
74377	Hsf2bp	heat shock transcription factor 2 binding protein	1.00E-05	0.170244	-1.41E+01
74400	Zfp819	zinc finger protein 819	1.00E-05	0.143084	-1.38E+01
74434	Sohlh2	spermatogenesis and oogenesis specific basic helix-loop-helix 2	1.00E-05	0.0557094	-1.24E+01
74446	Slc9b1	solute carrier family 9, subfamily B (NHA1, cation proton antiporter 1), member 1	1.00E-05	0.0537083	-1.24E+01
74466	4933427G17Rik	RIKEN cDNA 4933427G17 gene	0.423782	0.150055	1.50E+00
74472	4933433C11Rik	RIKEN cDNA 4933433C11 gene	1.00E-05	0.108856	-1.34E+01
74481	Batf2	basic leucine zipper transcription factor, ATF-like 2	0.218353	1.00E-05	1.44E+01
74488	Lrrc15	leucine rich repeat containing 15	1.00E-05	0.0225828	-1.11E+01
74489	5430421F17Rik	RIKEN cDNA 5430421F17 gene	0.0462857	0.149674	-1.69E+00
74499	Sost	sclerostin	1.00E-05	0.148482	-1.39E+01
74548	Gsdmc4	gasdermin C4	1.00E-05	0.0581034	-1.25E+01
74561	Nkx6-3	NK6 homeobox 3	1.00E-05	0.109415	-1.34E+01
74568	Mlkl	mixed lineage kinase domain-like	0.171652	1.00E-05	1.41E+01
74589	Kbtbd12	kelch repeat and BTB (POZ) domain containing 12	0.0524615	0.225077	-2.10E+00
74597	4833418N02Rik	RIKEN cDNA 4833418N02 gene	0.178323	0.647778	-1.86E+00

74712	4930511A02Rik	RIKEN cDNA 4930511A02 gene	1.00E-05	0.0537434	-1.24E+01
74716	Wbp2nl	WBP2 N-terminal like	1.00E-05	0.103098	-1.33E+01
74735	Trim14	tripartite motif-containing 14	0.851583	0.354987	1.26E+00
74748	Slamf8	SLAM family member 8	0.0541517	0.285134	-2.40E+00
74757	5830416I19Rik	RIKEN cDNA 5830416I19 gene	0.0484218	0.1028	-1.09E+00
74843	Mss51	MSS51 mitochondrial translational activator	1.00E-05	0.0653837	-1.27E+01
74846	4930405A21Rik	RIKEN cDNA 4930405A21 gene	0.427974	1.00E-05	1.54E+01
74890	Morn3	MORN repeat containing 3	0.122127	0.378227	-1.63E+00
74925	4930479D17Rik	RIKEN cDNA 4930479D17 gene	1.08308	0.383517	1.50E+00
74945	4930471I20Rik	RIKEN cDNA 4930471I20 gene	1.00E-05	0.44371	-1.54E+01
74959	4930500J02Rik	RIKEN cDNA 4930500J02 gene	1.00E-05	0.129842	-1.37E+01
75013	4930502E18Rik	RIKEN cDNA 4930502E18 gene	1.00E-05	0.323932	-1.50E+01
75019	Rnase10	ribonuclease, RNase A family, 10 (non-active)	0.14748	1.00E-05	1.38E+01
75033	Mei4	meiosis-specific, MEI4 homolog (S. cerevisiae)	0.674784	0.287468	1.23E+00
75095	4930513D17Rik	RIKEN cDNA 4930513D17 gene	1.00E-05	0.0946003	-1.32E+01
75149	4930539M17Rik	RIKEN cDNA 4930539M17 gene	1.00E-05	0.171434	-1.41E+01
75173	Tex38	testis expressed 38	0.578223	0.195199	1.57E+00
75188	1700009J07Rik	RIKEN cDNA 1700009J07 gene	1.00E-05	0.158112	-1.39E+01
75199	Rhox2a	reproductive homeobox 2A	1.00E-05	0.166614	-1.40E+01
75266	Tomm20l	translocase of outer mitochondrial membrane 20 homolog (yeast)-like	1.00E-05	0.299417	-1.49E+01
75275	Tmco5b	transmembrane and coiled-coil domains 5B	1.00E-05	0.0831183	-1.30E+01
75286	4930549G23Rik	RIKEN cDNA 4930549G23 gene	0.231105	0.473438	-1.03E+00
75394	0610040F04Rik	RIKEN cDNA 0610040F04 gene	0.139646	0.294899	-1.08E+00
75396	Spp2	secreted phosphoprotein 2	0.473409	0.16496	1.52E+00

75456	Prps1l1	phosphoribosyl pyrophosphate synthetase 1-like 1	1.00E-05	0.0748568	-1.29E+01
75479	1700012D14Rik	RIKEN cDNA 1700012D14 gene	0.676263	1.00E-05	1.60E+01
75480	1700003F12Rik	RIKEN cDNA 1700003F12 gene	0.600588	1.41316	-1.23E+00
75504	1700013F07Rik	RIKEN cDNA 1700013F07 gene	0.65902	1.66824	-1.34E+00
75516	Ttc32	tetratricopeptide repeat domain 32	1.64737	3.49705	-1.09E+00
75528	Tex29	testis expressed 29	1.00E-05	0.170149	-1.41E+01
75571	Spata9	spermatogenesis associated 9	1.91461	0.73056	1.39E+00
75641	1700029I15Rik	RIKEN cDNA 1700029I15 gene	1.00E-05	0.737461	-1.62E+01
75642	Spata25	spermatogenesis associated 25	1.00E-05	0.217879	-1.44E+01
75647	Ssmem1	serine-rich single-pass membrane protein 1	0.259807	0.0944254	1.46E+00
75657	Speer4a	spermatogenesis associated glutamate (E)-rich protein 4a	0.205146	0.0752984	1.45E+00
75677	Cldn22	claudin 22	0.761792	0.305233	1.32E+00
75753	Klf17	Kruppel-like factor 17	0.0293642	0.0623068	-1.09E+00
75766	Dcstamp	dentrocyte expressed seven transmembrane protein	0.19276	0.483992	-1.33E+00
75799	4930444P10Rik	RIKEN cDNA 4930444P10 gene	0.907055	0.390263	1.22E+00
75801	4930447C04Rik	RIKEN cDNA 4930447C04 gene	0.936578	1.90192	-1.02E+00
75814	4930467D21Rik	RIKEN cDNA 4930467D21 gene	0.221656	0.0815942	1.44E+00
75823	Fam227b	family with sequence similarity 227, member B	0.729295	0.234144	1.64E+00
75862	4930572O13Rik	RIKEN cDNA 4930572O13 gene	0.171091	0.366163	-1.10E+00
75880	4930592A05Rik	RIKEN cDNA 4930592A05 gene	0.611839	0.13069	2.23E+00
75939	4930579G24Rik	RIKEN cDNA 4930579G24 gene	1.07705	0.530474	1.02E+00
75951	4930578M01Rik	RIKEN cDNA 4930578M01 gene	0.152719	0.317135	-1.05E+00
76024	Gm11346	X-linked lymphocyte-regulated 5 pseudogene	0.0877565	0.19025	-1.12E+00
76062	5830428M24Rik	RIKEN cDNA 5830428M24 gene	1.00E-05	0.12361	-1.36E+01
76100	5830454E08Rik	RIKEN cDNA 5830454E08 gene	1.79684	0.55526	1.69E+00

76294	Asb5	ankyrin repeat and SOCs box-containing 5	0.787445	0.250131	1.65E+00
76400	Pbp2	phosphatidylethanolamine binding protein 2	0.188953	1.00E-05	1.42E+01
76406	1700019B03Rik	RIKEN cDNA 1700019B03 gene	1.00E-05	0.0862578	-1.31E+01
76415	Fam187b	family with sequence similarity 187, member B	0.237547	1.00E-05	1.45E+01
76419	1700023L04Rik	RIKEN cDNA 1700023L04 gene	0.219151	0.460754	-1.07E+00
76432	2310001H17Rik	RIKEN cDNA 2310001H17 gene	0.326292	1.59492	-2.29E+00
76464	Casc5	cancer susceptibility candidate 5	0.013148	0.0297875	-1.18E+00
76509	1600029D21Rik	RIKEN cDNA 1600029D21 gene	0.191423	0.0666179	1.52E+00
76560	Prss8	protease, serine, 8 (prostasin)	0.160917	1.00E-05	1.40E+01
76615	Got1l1	glutamic-oxaloacetic transaminase 1-like 1	0.726392	0.286354	1.34E+00
76645	Pkd1l2	polycystic kidney disease 1 like 2	0.033721	0.0168487	1.00E+00
76681	Trim12a	tripartite motif-containing 12A	0.118808	0.387513	-1.71E+00
76703	Cpb1	carboxypeptidase B1 (tissue)	0.0713198	0.152164	-1.09E+00
76718	Catsperg2	catsper channel auxiliary subunit gamma 2	1.00E-05	0.0618532	-1.26E+01
76798	2410137F16Rik	RIKEN cDNA 2410137F16 gene	0.0689416	0.260152	-1.92E+00
76905	Lrg1	leucine-rich alpha-2-glycoprotein 1	0.317422	1.3222	-2.06E+00
76927	Tsacc	TSSK6 activating co-chaperone	1.91809	0.391019	2.29E+00
76964	2610028H24Rik	RIKEN cDNA 2610028H24 gene	1.00E-05	0.0701226	-1.28E+01
77011	Ticrr	TOPBP1-interacting checkpoint and replication regulator	0.0435672	0.0154947	1.49E+00
77037	Mrap	melanocortin 2 receptor accessory protein	1.00E-05	0.150099	-1.39E+01
77119	7630403G23Rik	RIKEN cDNA 7630403G23 gene	1.00E-05	0.106728	-1.34E+01
77132	2810433D01Rik	RIKEN cDNA 2810433D01 gene	0.0905904	0.290822	-1.68E+00
77382	C030018K13Rik	RIKEN cDNA C030018K13 gene	2.21892	0.902296	1.30E+00
77595	Nup210l	nucleoporin 210-like	0.0550165	0.116296	-1.08E+00
77647	Trat1	T cell receptor associated transmembrane adaptor 1	1.00E-05	0.192637	-1.42E+01

77675	5033406009Rik	RIKEN cDNA 5033406009 gene	0.140136	0.291257	-1.06E+00
77682	9230102004Rik	RIKEN cDNA 9230102004 gene	1.00E-05	0.217677	-1.44E+01
77717	6030408B16Rik	RIKEN cDNA 6030408B16 gene	1.00E-05	0.142727	-1.38E+01
77727	6030468B19Rik	RIKEN cDNA 6030468B19 gene	1.00E-05	0.145953	-1.38E+01
77741	6720483E21Rik	RIKEN cDNA 6720483E21 gene	0.232046	1.00E-05	1.45E+01
77798	A930009A15Rik	RIKEN cDNA A930009A15 gene	0.125275	0.387324	-1.63E+00
77836	Mlana	melan-A	1.00E-05	0.406227	-1.53E+01
77905	Fate1	fetal and adult testis expressed 1	1.00E-05	0.164306	-1.40E+01
77994	2810055G20Rik	RIKEN cDNA 2810055G20 gene	1.00E-05	0.158614	-1.40E+01
78004	Prr15	proline rich 15	0.362628	0.852196	-1.23E+00
78052	Tmem190	transmembrane protein 190	1.00E-05	0.292562	-1.48E+01
78058	4930578E11Rik	RIKEN cDNA 4930578E11 gene	0.0937891	1.00E-05	1.32E+01
78060	4930593C16Rik	RIKEN cDNA 4930593C16 gene	1.00E-05	0.307894	-1.49E+01
78174	Cox7b2	cytochrome c oxidase subunit VIIb2	0.814645	1.00E-05	1.63E+01
78194	4930546K05Rik	RIKEN cDNA 4930546K05 gene	1.00E-05	0.0316776	-1.16E+01
78217	Tmem210	transmembrane protein 210	1.00E-05	0.397435	-1.53E+01
78245	Acbd7	acyl-Coenzyme A binding domain containing 7	0.789944	1.00E-05	1.63E+01
78249	Gpr115	G protein-coupled receptor 115	0.349588	0.0814515	2.10E+00
78321	Ankrd23	ankyrin repeat domain 23	0.435113	1.11743	-1.36E+00
78329	2310010J17Rik	RIKEN cDNA 2310010J17 gene	2.15399	0.548129	1.97E+00
78365	1500016L03Rik	RIKEN cDNA 1500016L03 gene	0.23541	1.20318	-2.35E+00
78369	Icam4	intercellular adhesion molecule 4, Landsteiner-Wiener blood group	1.80304	0.784848	1.20E+00
78405	Ntf5	neurotrophin 5	0.139475	0.351991	-1.34E+00
78449	2700046A07Rik	RIKEN cDNA 2700046A07 gene	0.0979608	0.313572	-1.68E+00

78482	1700123L14Rik	nucleoporin 50 pseudogene	0.188674	0.0661302	1.51E+00
78500	1700063D05Rik	RIKEN cDNA 1700063D05 gene	1.21208	0.428092	1.50E+00
78547	E130304I02Rik	RIKEN cDNA E130304I02 gene	1.00E-05	0.382128	-1.52E+01
78575	B430319G15Rik	RIKEN cDNA B430319G15 gene	0.169231	0.546514	-1.69E+00
78600	Pde6h	phosphodiesterase 6H, cGMP-specific, cone, gamma	1.00E-05	0.286036	-1.48E+01
78625	1700061G19Rik	RIKEN cDNA 1700061G19 gene	0.142115	0.0491149	1.53E+00
78635	1700095B22Rik	RIKEN cDNA 1700095B22 gene	0.17352	0.50874	-1.55E+00
78753	Lipm	lipase, family member M	0.114002	0.0414193	1.46E+00
78754	Galnt15	UDP-N-acetyl-alpha-D-galactosamine:polypeptide N-acetylgalactosaminyltransferase 15	1.1426	0.520459	1.13E+00
78767	Efcab11	EF-hand calcium binding domain 11	1.00E-05	0.180239	-1.41E+01
78800	4930480K15Rik	RIKEN cDNA 4930480K15 gene	1.00E-05	0.0558446	-1.24E+01
78803	Fbxo43	F-box protein 43	1.00E-05	0.0292955	-1.15E+01
78806	Stpg1	sperm tail PG rich repeat containing 1	0.167042	0.961719	-2.53E+00
78977	Popdc3	popeye domain containing 3	1.49132	0.697273	1.10E+00
79202	Tnfrsf22	tumor necrosis factor receptor superfamily, member 22	0.497938	0.998931	-1.00E+00
79235	Lrat	lecithin-retinol acyltransferase (phosphatidylcholine-retinol-O-acyltransferase)	0.0581266	0.148258	-1.35E+00
79459	Aldoart2	aldolase 1 A retrogene 2	0.15993	1.00E-05	1.40E+01
80782	Klrb1b	killer cell lectin-like receptor subfamily B member 1B	0.047315	0.31344	-2.73E+00
80794	Cblc	Casitas B-lineage lymphoma c	1.00E-05	0.0840841	-1.30E+01
80797	Clca2	chloride channel calcium activated 2	1.00E-05	0.0292315	-1.15E+01
80893	Tmprss5	transmembrane protease, serine 5 (spinesin)	0.365308	0.862414	-1.24E+00
80903	Fgf16	fibroblast growth factor 16	0.294503	0.079518	1.89E+00
80978	Mrgprh	MAS-related GPR, member H	0.0743937	0.154416	-1.05E+00

80986	Ckap2	cytoskeleton associated protein 2	0.115339	0.291832	-1.34E+00
81014	Vmn1r58	vomeronasal 1 receptor 58	0.0376438	0.0813176	-1.11E+00
81701	Egfl8	EGF-like domain 8	1.23452	0.599878	1.04E+00
81799	C1qtnf3	C1q and tumor necrosis factor related protein 3	0.139936	0.0509705	1.46E+00
83379	Klb	klotho beta	1.00E-05	0.0687425	-1.27E+01
83395	Sp6	trans-acting transcription factor 6	0.277146	0.121841	1.19E+00
83408	Gimap3	GTPase, IMAP family member 3	0.162746	1.00E-05	1.40E+01
83430	Il23a	interleukin 23, alpha subunit p19	0.0856855	0.181513	-1.08E+00
83434	Rsph6a	radial spoke head 6 homolog A (Chlamydomonas)	0.159245	0.0746732	1.09E+00
83492	Gsdmc	gasdermin C	1.00E-05	0.097289	-1.32E+01
83553	Tktl1	transketolase-like 1	1.00E-05	0.0474417	-1.22E+01
83556	Tex16	testis expressed gene 16	1.00E-05	0.0253172	-1.13E+01
83558	Tex11	testis expressed gene 11	0.0391931	0.098133	-1.32E+00
83672	Sytl3	synaptotagmin-like 3	0.233514	1.00E-05	1.45E+01
83885	Slc25a2	solute carrier family 25 (mitochondrial carrier, ornithine transporter) member 2	1.00E-05	0.273848	-1.47E+01
84544	Cd96	CD96 antigen	1.00E-05	0.0493208	-1.23E+01
84682	Cox4i2	cytochrome c oxidase subunit IV isoform 2	1.11691	2.93691	-1.39E+00
84704	Snurf	SNRPN upstream reading frame	0.146482	127.927	-9.77E+00
85029	Rpph1	ribonuclease P RNA component H1	0.842209	2.48954	-1.56E+00
93675	Clec2i	C-type lectin domain family 2, member i	1.00E-05	0.281625	-1.48E+01
93721	Cpn1	carboxypeptidase N, polypeptide 1	0.510432	0.218726	1.22E+00
93735	Wnt16	wingless-related MMTV integration site 16	0.723045	0.326992	1.14E+00
93746	Gprc5d	G protein-coupled receptor, family C, group 5, member D	1.00E-05	0.114057	-1.35E+01
93841	Uchl4	ubiquitin carboxyl-terminal esterase L4	1.00E-05	0.0967461	-1.32E+01

94041	Allc	allantoicase	1.00E-05	0.0792504	-1.30E+01
94071	Clec2h	C-type lectin domain family 2, member h	1.00E-05	0.052687	-1.24E+01
94179	Krt23	keratin 23	1.00E-05	0.139902	-1.38E+01
94215	Ugt2a1	UDP glucuronosyltransferase 2 family, polypeptide A1	1.00E-05	0.0467224	-1.22E+01
94222	Olig3	oligodendrocyte transcription factor 3	1.00E-05	0.223249	-1.44E+01
94224	Srd5a2	steroid 5 alpha-reductase 2	1.00E-05	0.210354	-1.44E+01
94244	Fkbp6	FK506 binding protein 6	0.307633	0.113319	1.44E+00
94284	Ugt1a6a	UDP glucuronosyltransferase 1 family, polypeptide A6A	0.101382	0.331469	-1.71E+00
97243	Naa11	N(alpha)-acetyltransferase 11, NatA catalytic subunit	0.139301	1.00E-05	1.38E+01
97895	Nlrp4f	NLR family, pyrin domain containing 4F	0.0266555	0.0896407	-1.75E+00
98365	Slamf9	SLAM family member 9	1.59757	0.716918	1.16E+00
98452	B230209K01Rik	RIKEN cDNA B230209K01 gene	0.3428	1.04445	-1.61E+00
98558	Mael	maelstrom homolog (Drosophila)	0.162895	1.00E-05	1.40E+01
98870	AI182371	expressed sequence AI182371	0.126659	0.338671	-1.42E+00
99663	Clca6	chloride channel calcium activated 6	1.00E-05	0.0330459	-1.17E+01
100061	Lrrc19	leucine rich repeat containing 19	1.00E-05	0.0426252	-1.21E+01
100689	Spon2	spondin 2, extracellular matrix protein	1.00E-05	0.108679	-1.34E+01
100740	AI839979	expressed sequence AI839979	0.0946687	0.707717	-2.90E+00
101320	Dyrk4	dual-specificity tyrosine-(Y)-phosphorylation regulated kinase 4	0.0958447	1.00E-05	1.32E+01
101533	Klk9	kallikrein related-peptidase 9	1.00E-05	0.0981594	-1.33E+01
102570	Slc22a13	solute carrier family 22 (organic cation transporter), member 13	1.00E-05	0.27735	-1.48E+01
102920	Cenpi	centromere protein I	0.317266	0.114817	1.47E+00
102991	AU022751	expressed sequence AU022751	1.00E-05	0.0691601	-1.28E+01

103142	Rdh9	retinol dehydrogenase 9	0.0422988	0.226407	-2.42E+00
103149	Upb1	ureidopropionase, beta	0.243785	0.502452	-1.04E+00
103220	BC030307	cDNA sequence BC030307	0.551908	0.210172	1.39E+00
103655	Sec14l4	SEC14-like 4 (S. cerevisiae)	0.345567	0.118284	1.55E+00
103729	AI450353	expressed sequence AI450353	1.12594	2.39706	-1.09E+00
104158	Ces1d	carboxylesterase 1D	0.168747	1.00E-05	1.40E+01
104362	Meig1	meiosis expressed gene 1	2.76261	1.09687	1.33E+00
104382	Barhl2	BarH-like 2 (Drosophila)	0.881759	0.392585	1.17E+00
104522	AU040972	expressed sequence AU040972	0.446385	1.00E-05	1.54E+01
104943	Fam110c	family with sequence similarity 110, member C	0.213285	0.0903224	1.24E+00
105243	Slc9a3	solute carrier family 9 (sodium/hydrogen exchanger), member 3	0.0478365	0.148136	-1.63E+00
105418	E330034G19Rik	RIKEN cDNA E330034G19 gene	0.120978	1.00E-05	1.36E+01
105785	Kdelr3	KDEL (Lys-Asp-Glu-Leu) endoplasmic reticulum protein retention receptor 3	0.471708	0.19404	1.28E+00
106347	Ildr1	immunoglobulin-like domain containing receptor 1	0.234985	0.0482659	2.28E+00
106557	Ldhal6b	lactate dehydrogenase A-like 6B	1.00E-05	0.0885561	-1.31E+01
107221	Ffar4	free fatty acid receptor 4	1.00E-05	0.133906	-1.37E+01
107753	Lgals2	lectin, galactose-binding, soluble 2	1.00E-05	0.247783	-1.46E+01
107766	Haao	3-hydroxyanthranilate 3,4-dioxygenase	0.5359	1.00E-05	1.57E+01
107770	Tm6sf2	transmembrane 6 superfamily member 2	0.850784	0.241187	1.82E+00
107993	Bfsp2	beaded filament structural protein 2, phakinin	0.436326	1.03764	-1.25E+00
108072	Grm6	glutamate receptor, metabotropic 6	1.00E-05	0.0320589	-1.16E+01
108112	Eif4ebp3	eukaryotic translation initiation factor 4E binding protein 3	0.962402	2.34306	-1.28E+00

108114	Slc22a7	solute carrier family 22 (organic anion transporter), member 7	1.00E-05	0.213908	-1.44E+01
108153	Adamts7	a disintegrin-like and metallopeptidase (reprolysin type) with thrombospondin type 1 motif, 7	0.0236259	0.0705222	-1.58E+00
108670	Epsti1	epithelial stromal interaction 1 (breast)	0.162739	0.344755	-1.08E+00
108723	Card11	caspase recruitment domain family, member 11	0.0536631	0.164509	-1.62E+00
108900	Fam72a	family with sequence similarity 72, member A	0.60937	0.302929	1.01E+00
108961	E2f8	E2F transcription factor 8	0.208806	0.0929913	1.17E+00
108978	4930555G01Rik	RIKEN cDNA 4930555G01 gene	0.464709	0.206289	1.17E+00
109032	Sp110	Sp110 nuclear body protein	0.755263	1.58184	-1.07E+00
109052	Krt75	keratin 75	1.00E-05	0.0347719	-1.18E+01
109212	Fam64a	family with sequence similarity 64, member A	0.164524	0.585766	-1.83E+00
109225	Ms4a7	membrane-spanning 4-domains, subfamily A, member 7	0.77425	0.185295	2.06E+00
109245	Lrrc39	leucine rich repeat containing 39	0.033827	0.263971	-2.96E+00
109267	Srcrb4d	scavenger receptor cysteine rich domain containing, group B (4 domains)	0.211037	0.519362	-1.30E+00
109332	Cdcp1	CUB domain containing protein 1	0.172896	0.0300569	2.52E+00
110115	Cyp11b1	cytochrome P450, family 11, subfamily b, polypeptide 1	1.00E-05	0.0533856	-1.24E+01
110257	Hba-a2	hemoglobin alpha, adult chain 2	61.1632	145.931	-1.25E+00
110308	Krt5	keratin 5	1.00E-05	0.055732	-1.24E+01
110310	Krt7	keratin 7	0.078624	0.238994	-1.60E+00
110558	H2-Q9	histocompatibility 2, Q region locus 9	0.414717	1.00E-05	1.53E+01
110648	Lmx1a	LIM homeobox transcription factor 1 alpha	0.0995374	0.245629	-1.30E+00
111241	Hmga1-rs1	high mobility group AT-hook I, related sequence 1	18.6648	5.60629	1.74E+00
111368	Prn	prion protein gene complex	1.00E-05	0.077984	-1.29E+01

112418	1700102P08Rik	RIKEN cDNA 1700102P08 gene	0.107561	0.571829	-2.41E+00
112422	2610305D13Rik	RIKEN cDNA 2610305D13 gene	0.347171	0.839513	-1.27E+00
114230	Aipl1	aryl hydrocarbon receptor-interacting protein-like 1	0.159045	1.00E-05	1.40E+01
114304	Slc28a3	solute carrier family 28 (sodium-coupled nucleoside transporter), member 3	0.0513477	0.217061	-2.08E+00
114640	Pth2	parathyroid hormone 2	1.00E-05	0.456217	-1.55E+01
114774	Pawr	PRKC, apoptosis, WT1, regulator	1.0953	0.545442	1.01E+00
114875	Plcz1	phospholipase C, zeta 1	0.176694	0.58633	-1.73E+00
116903	Calcb	calcitonin-related polypeptide, beta	1.00E-05	0.150315	-1.39E+01
116904	Alpk3	alpha-kinase 3	0.0668792	0.017275	1.95E+00
117167	Steap4	STEAP family member 4	0.478541	0.0390084	3.62E+00
117229	Stk33	serine/threonine kinase 33	0.965534	0.465293	1.05E+00
117591	Slc2a9	solute carrier family 2 (facilitated glucose transporter), member 9	0.485687	0.231427	1.07E+00
140494	Atp6v0a4	ATPase, H+ transporting, lysosomal V0 subunit A4	0.102735	1.00E-05	1.33E+01
140806	Il25	interleukin 25	1.00E-05	0.293373	-1.48E+01
142681	Slc34a3	solute carrier family 34 (sodium phosphate), member 3	0.40501	0.158853	1.35E+00
170484	Nphs2	nephrosis 2 homolog, podocin (human)	0.107244	0.383995	-1.84E+00
170639	Olfr78	olfactory receptor 78	0.101682	0.485081	-2.25E+00
170722	Nxf7	nuclear RNA export factor 7	0.388268	0.833028	-1.10E+00
170734	Zscan5b	zinc finger and SCAN domain containing 5B	1.00E-05	0.0561108	-1.25E+01
170735	Arr3	arrestin 3, retinal	0.786476	0.273397	1.52E+00
170745	Xpnpep2	X-prolyl aminopeptidase (aminopeptidase P) 2, membrane-bound	0.0950251	0.196125	-1.05E+00
170761	Pdzd3	PDZ domain containing 3	0.529119	0.238368	1.15E+00
170765	Ripply3	ripply3 homolog (zebrafish)	0.164741	0.343905	-1.06E+00

170776	Cd209c	CD209c antigen	0.23619	0.652961	-1.47E+00
170829	Tram2	translocating chain-associating membrane protein 2	0.0997066	0.409465	-2.04E+00
171170	Mbnl3	muscleblind-like 3 (Drosophila)	0.0366205	0.013495	1.44E+00
171209	Asic3	acid-sensing (proton-gated) ion channel 3	1.00E-05	0.0726842	-1.28E+01
171211	Edaradd	EDAR (ectodysplasin-A receptor)-associated death domain	0.0784549	0.179804	-1.20E+00
171250	Vmn1r206	vomeronasal 1 receptor 206	0.300178	1.00E-05	1.49E+01
171504	Apobr	apolipoprotein B receptor	0.516858	0.251921	1.04E+00
192113	Atp12a	ATPase, H+/K+ transporting, nongastric, alpha polypeptide	0.0544065	1.00E-05	1.24E+01
192212	Prom2	prominin 2	0.0309585	0.094022	-1.60E+00
192653	Ttc36	tetratricopeptide repeat domain 36	1.00E-05	0.215279	-1.44E+01
193003	Pirt	phosphoinositide-interacting regulator of transient receptor potential channels	0.280578	0.595641	-1.09E+00
193034	Trpv1	transient receptor potential cation channel, subfamily V, member 1	1.00E-05	0.0468327	-1.22E+01
193217	BC018473	cDNA sequence BC018473	0.0323598	0.13934	-2.11E+00
193286	BC049762	cDNA sequence BC049762	1.00E-05	0.15979	-1.40E+01
194219	Slfnl1	schlafen like 1	1.00E-05	0.0769697	-1.29E+01
194292	Gm5	predicted gene 5	1.00E-05	0.745938	-1.62E+01
194908	Pld6	phospholipase D family, member 6	1.08759	0.508928	1.10E+00
194974	Sun3	Sad1 and UNC84 domain containing 3	0.281892	0.101663	1.47E+00
195564	Skint3	selection and upkeep of intraepithelial T cells 3	0.0794335	1.00E-05	1.30E+01
208084	Pif1	PIF1 5’-to-3’ DNA helicase homolog (S. cerevisiae)	0.0357249	0.10861	-1.60E+00
208372	Asb18	ankyrin repeat and SOCS box-containing 18	0.790484	0.296142	1.42E+00
208628	Kntc1	kinetochore associated 1	0.152036	0.0647199	1.23E+00
208634	Tspan10	tetraspanin 10	1.00E-05	0.0698707	-1.28E+01

208990	Npb	neuropeptide B	0.378553	1.12509	-1.57E+00
209294	Csta	cystatin A	0.0830226	1.00E-05	1.30E+01
209387	Trim30d	tripartite motif-containing 30D	0.221408	0.0884274	1.32E+00
209737	Kif15	kinesin family member 15	0.0538927	0.139798	-1.38E+00
210145	Irgc1	immunity-related GTPase family, cinema 1	0.0983548	0.476346	-2.28E+00
210198	Gprc6a	G protein-coupled receptor, family C, group 6, member A	0.0783433	1.00E-05	1.29E+01
210710	Gab3	growth factor receptor bound protein 2-associated protein 3	0.72867	0.312161	1.22E+00
210757	Themis	thymocyte selection associated	1.00E-05	0.0276873	-1.14E+01
211001	8030423F21Rik	RIKEN cDNA 8030423F21 gene	0.0387105	0.0833827	-1.11E+00
211228	Lrrc25	leucine rich repeat containing 25	0.229821	0.67608	-1.56E+00
211305	Fbxw13	F-box and WD-40 domain protein 13	1.00E-05	0.0779806	-1.29E+01
211480	Kcnj14	potassium inwardly-rectifying channel, subfamily J, member 14	1.11404	0.545231	1.03E+00
211535	Ccdc114	coiled-coil domain containing 114	0.337683	0.892598	-1.40E+00
211578	Mrgprd	MAS-related GPR, member D	1.00E-05	0.0393591	-1.19E+01
211623	Plac9a	placenta specific 9a	1.28614	4.33644	-1.75E+00
211666	Mgst2	microsomal glutathione S-transferase 2	0.265422	0.554051	-1.06E+00
212377	Mms221	MMS22-like, DNA repair protein	0.0526564	0.392252	-2.90E+00
212541	Rho	rhodopsin	0.0748026	0.155359	-1.05E+00
212980	Slc45a3	solute carrier family 45, member 3	0.265983	0.614747	-1.21E+00
212998	BC016579	cDNA sequence, BC016579	0.476966	0.228773	1.06E+00
213234	Zbbx	zinc finger, B-box domain containing	0.179551	0.0801997	1.16E+00
213272	Txndc2	thioredoxin domain containing 2 (spermatozoa)	0.0475482	0.116983	-1.30E+00
213393	8430408G22Rik	RIKEN cDNA 8430408G22 gene	0.216799	0.435495	-1.01E+00
213409	Lemd1	LEM domain containing 1	1.2559	0.520232	1.27E+00
213436	Zcchc5	zinc finger, CCHC domain containing 5	0.342042	0.0395883	3.11E+00

213765	Nutm1	NUT midline carcinoma, family member 1	0.0285403	0.0908396	-1.67E+00
213956	Fam83f	family with sequence similarity 83, member F	0.0879667	0.220384	-1.32E+00
214105	Sox30	SRY-box containing gene 30	1.00E-05	0.048391	-1.22E+01
214191	Ttc24	tetratricopeptide repeat domain 24	1.00E-05	0.0524479	-1.24E+01
214301	Crygn	crystallin, gamma N	1.00E-05	0.630856	-1.59E+01
214523	Tmprss4	transmembrane protease, serine 4	1.00E-05	0.0522986	-1.24E+01
214639	4930486L24Rik	RIKEN cDNA 4930486L24 gene	0.0730352	0.235757	-1.69E+00
214704	Iqub	IQ motif and ubiquitin domain containing	0.300126	0.862373	-1.52E+00
214763	Mb21d1	Mab-21 domain containing 1	0.133307	0.375306	-1.49E+00
214854	Neurl3	neuralized homolog 3 homolog (Drosophila)	0.481722	0.19974	1.27E+00
214901	Chtf18	CTF18, chromosome transmission fidelity factor 18	0.30663	0.646555	-1.08E+00
215031	Vgll2	vestigial like 2 homolog (Drosophila)	1.00E-05	0.11857	-1.35E+01
215384	Fcgbp	Fc fragment of IgG binding protein	0.0311964	0.12313	-1.98E+00
215493	A3galt2	alpha 1,3-galactosyltransferase 2 (isoglobotriaosylceramide synthase)	1.00E-05	0.0811357	-1.30E+01
215772	Adgb	androglobin	0.110985	0.259803	-1.23E+00
216225	Slc5a8	solute carrier family 5 (iodide transporter), member 8	0.174798	0.0238644	2.87E+00
216343	Tph2	tryptophan hydroxylase 2	3.76502	1.38714	1.44E+00
216350	Tspan8	tetraspanin 8	0.265728	1.01804	-1.94E+00
216749	Nmur2	neuromedin U receptor 2	0.219441	0.0773723	1.50E+00
216781	Trim58	tripartite motif-containing 58	0.114659	1.00E-05	1.35E+01
216783	Olfr320	olfactory receptor 320	1.00E-05	0.190645	-1.42E+01
216858	Kctd11	potassium channel tetramerisation domain containing 11	0.529857	1.08124	-1.03E+00
216859	Acap1	ArfGAP with coiled-coil, ankyrin repeat and PH domains 1	0.095411	1.00E-05	1.32E+01
216871	Gltpd2	glycolipid transfer protein domain containing 2	1.00E-05	0.347432	-1.51E+01

216974	Proca1	protein interacting with cyclin A1	2.02076	0.916899	1.14E+00
216984	Evi2b	ecotropic viral integration site 2b	1.00E-05	0.157869	-1.39E+01
217246	Ace3	angiotensin I converting enzyme (peptidyl-dipeptidase A) 3	1.00E-05	0.0559346	-1.24E+01
217294	BC006965	cDNA sequence BC006965	0.40208	0.124087	1.70E+00
217306	Cd300e	CD300e antigen	0.222222	1.00E-05	1.44E+01
217325	Llgl2	lethal giant larvae homolog 2 (Drosophila)	0.568768	0.251991	1.17E+00
217344	Rhbdf2	rhomboid 5 homolog 2 (Drosophila)	0.220355	0.76666	-1.80E+00
217369	Uts2r	urotensin 2 receptor	1.00E-05	0.191438	-1.42E+01
217845	Ifi27l2b	interferon, alpha-inducible protein 27 like 2B	0.329305	1.00E-05	1.50E+01
217847	Serpina10	serine (or cysteine) peptidase inhibitor, clade A (alpha-1 antiproteinase, antitrypsin), member 10	1.00E-05	0.0700876	-1.28E+01
218624	Il31ra	interleukin 31 receptor A	0.083438	0.0297942	1.49E+00
218739	Sntn	sentan, cilia apical structure protein	0.552094	0.198152	1.48E+00
219131	Phf11a	PHD finger protein 11A	0.249721	0.0895653	1.48E+00
223262	Timm8a2	translocase of inner mitochondrial membrane 8A2	1.00E-05	0.163276	-1.40E+01
223645	Mroh6	maestro heat-like repeat family member 6	1.00E-05	0.0857723	-1.31E+01
223672	Apol9a	apolipoprotein L 9a	0.0767315	0.456656	-2.57E+00
223917	Krt79	keratin 79	1.00E-05	0.0589399	-1.25E+01
223920	Soat2	sterol O-acyltransferase 2	1.00E-05	0.065559	-1.27E+01
223970	Rmi2	RMI2, RecQ mediated genome instability 2, homolog (S. cerevisiae)	0.106582	1.00E-05	1.34E+01
224055	Rtp2	receptor transporter protein 2	1.00E-05	0.113491	-1.35E+01
224291	Csnka2ip	casein kinase 2, alpha prime interacting protein	1.00E-05	0.115727	-1.35E+01
224419	Map3k7cl	Map3k7 C-terminal like	0.15071	0.553225	-1.88E+00
224480	Nox3	NADPH oxidase 3	1.00E-05	0.0699216	-1.28E+01

224840	Treml4	triggering receptor expressed on myeloid cells-like 4	0.0680071	0.286571	-2.08E+00
225004	BC027072	cDNA sequence BC027072	0.0216854	0.0445067	-1.04E+00
225518	Prdm6	PR domain containing 6	0.0583318	0.348444	-2.58E+00
225579	Slc27a6	solute carrier family 27 (fatty acid transporter), member 6	0.122903	0.0442881	1.47E+00
225825	Cd226	CD226 antigen	0.0477578	0.139089	-1.54E+00
226143	Cyp2c44	cytochrome P450, family 2, subfamily c, polypeptide 44	1.00E-05	0.20111	-1.43E+01
226245	Plekhs1	pleckstrin homology domain containing, family S member 1	0.129715	0.045966	1.50E+00
226278	Prlhr	prolactin releasing hormone receptor	0.593947	0.225046	1.40E+00
226439	Ascl5	achaete-scute complex homolog 5 (Drosophila)	1.00E-05	0.466864	-1.55E+01
227231	Cps1	carbamoyl-phosphate synthetase 1	0.0214999	0.0682665	-1.67E+00
227358	Fam132b	family with sequence similarity 132, member B	0.430487	0.143795	1.58E+00
227618	Lrrc26	leucine rich repeat containing 26	1.09017	0.517332	1.08E+00
227631	Sohlh1	spermatogenesis and oogenesis specific basic helix-loop-helix 1	0.216837	1.00E-05	1.44E+01
227671	Gbgt1	globoside alpha-1,3-N-acetylgalactosaminyltransferase 1	0.305877	0.627177	-1.04E+00
227733	Pip5kl1	phosphatidylinositol-4-phosphate 5-kinase-like 1	1.61395	3.53827	-1.13E+00
228151	4833423E24Rik	RIKEN cDNA 4833423E24 gene	0.198232	0.419499	-1.08E+00
228366	Gyltl1b	glycosyltransferase-like 1B	1.00E-05	0.0626665	-1.26E+01
228413	Prrg4	proline rich Gla (G-carboxyglutamic acid) 4 (transmembrane)	0.583437	0.126553	2.20E+00
228576	Mall	mal, T cell differentiation protein-like	0.13473	0.557839	-2.05E+00
228677	Sptlc3	serine palmitoyltransferase, long chain base subunit 3	0.251299	0.1181	1.09E+00
228731	Nkx2-4	NK2 transcription factor related, locus 4 (Drosophila)	0.598889	0.270311	1.15E+00
228756	Cstl1	cystatin-like 1	0.231479	0.471789	-1.03E+00
228770	Rspo4	R-spondin family, member 4	0.239602	0.0611596	1.97E+00
228993	Slc17a9	solute carrier family 17, member 9	0.19819	1.00E-05	1.43E+01

229600	BC028528	cDNA sequence BC028528	1.43011	3.50167	-1.29E+00
229697	Cym	chymosin	1.00E-05	0.110467	-1.34E+01
230025	Prdm13	PR domain containing 13	0.114514	0.0552256	1.05E+00
230099	Car9	carbonic anhydrase 9	0.131056	0.471	-1.85E+00
230726	Rhbdl2	rhomboid, veinlet-like 2 (Drosophila)	1.00E-05	0.226024	-1.45E+01
230738	Zc3h12a	zinc finger CCCH type containing 12A	0.285942	0.660822	-1.21E+00
230822	Ncmap	noncompact myelin associated protein	0.146674	0.453348	-1.63E+00
230828	Il22ra1	interleukin 22 receptor, alpha 1	0.0372755	1.00E-05	1.19E+01
230979	Tnfrsf14	tumor necrosis factor receptor superfamily, member 14 (herpesvirus entry mediator)	0.567856	0.14359	1.98E+00
231296	Lrrc66	leucine rich repeat containing 66	1.00E-05	0.0349448	-1.18E+01
231507	Plac8	placenta-specific 8	1.00E-05	0.460258	-1.55E+01
231602	P2rx2	purinergic receptor P2X, ligand-gated ion channel, 2	1.00E-05	0.148876	-1.39E+01
231603	A630023P12Rik	RIKEN cDNA A630023P12 gene	0.554032	1.00E-05	1.58E+01
231655	Oasl1	2'-5' oligoadenylate synthetase-like 1	1.00E-05	0.120931	-1.36E+01
231727	B3gnt4	UDP-GlcNAc:betaGal beta-1,3-N-acetylglucosaminyltransferase 4	1.00E-05	0.828583	-1.63E+01
231946	Fam221a	family with sequence similarity 221, member A	0.300701	0.716855	-1.25E+00
232016	Ccdc129	coiled-coil domain containing 129	0.22575	0.456713	-1.02E+00
232367	Vmn2r27	vomeronasal 2, receptor27	1.00E-05	0.0465574	-1.22E+01
232371	C1rl	complement component 1, r subcomponent-like	0.106157	0.259429	-1.29E+00
232414	Clec9a	C-type lectin domain family 9, member a	0.0397884	0.0860757	-1.11E+00
232585	Vwde	von Willebrand factor D and EGF domains	0.0528011	1.00E-05	1.24E+01
232714	Mgam	maltase-glucoamylase	0.0156526	0.0332378	-1.09E+00
232790	Oscar	osteoclast associated receptor	0.255309	0.603398	-1.24E+00

232801	Lilra5	leukocyte immunoglobulin-like receptor, subfamily A (with TM domain), member 5	0.325487	1.00E-05	1.50E+01
232889	Pla2g4c	phospholipase A2, group IVC (cytosolic, calcium-independent)	0.0303563	0.0672977	-1.15E+00
233038	Nccrp1	non-specific cytotoxic cell receptor protein 1 homolog (zebrafish)	0.417173	1.00E-05	1.53E+01
233079	Ffar2	free fatty acid receptor 2	0.0679591	0.212741	-1.65E+00
233231	Mrgprb1	MAS-related GPR, member B1	1.00E-05	0.0322701	-1.17E+01
233575	Pgap2	post-GPI attachment to proteins 2	3.44315	7.64258	-1.15E+00
233801	Acsm4	acyl-CoA synthetase medium-chain family member 4	1.00E-05	0.103044	-1.33E+01
234072	Adprhl1	ADP-ribosylhydrolase like 1	0.175743	1.00E-05	1.41E+01
234404	Nxnl1	nucleoredoxin-like 1	0.0872734	1.00E-05	1.31E+01
234479	Gm4890	predicted gene 4890	0.0931812	0.339596	-1.87E+00
234684	Lrrc29	leucine rich repeat containing 29	0.471695	0.228843	1.04E+00
234700	Nrn1l	neuritin 1-like	0.331245	0.979136	-1.56E+00
234724	Tat	tyrosine aminotransferase	0.101671	1.00E-05	1.33E+01
234788	Slc38a8	solute carrier family 38, member 8	0.0539034	0.162247	-1.59E+00
235533	Gk5	glycerol kinase 5 (putative)	0.338995	0.805352	-1.25E+00
236312	Pyhin1	pyrin and HIN domain family, member 1	1.00E-05	0.0560443	-1.25E+01
237091	Lhfpl1	lipoma HMGIC fusion partner-like 1	1.00E-05	0.145286	-1.38E+01
237360	Adamts14	a disintegrin-like and metallopeptidase (reprolysin type) with thrombospondin type 1 motif, 14	0.12446	1.00E-05	1.36E+01
237465	Ccdc38	coiled-coil domain containing 38	0.0507842	0.141526	-1.48E+00
237560	Lrrc10	leucine rich repeat containing 10	1.00E-05	0.103725	-1.33E+01
237636	Npc1l1	NPC1-like 1	0.154439	0.309669	-1.00E+00
237749	Gm4926	T-cell immunoglobulin and mucin domain containing 2	0.211019	0.0765633	1.46E+00

		pseudogene			
237880	1700071K01Rik	RIKEN cDNA 1700071K01 gene	0.395797	1.06128	-1.42E+00
238021	Fscn2	fascin homolog 2, actin-bundling protein, retinal (Strongylocentrotus purpuratus)	1.00E-05	0.085554	-1.31E+01
238555	Btn2a2	butyrophilin, subfamily 2, member A2	0.284992	0.764723	-1.42E+00
238564	Mylk4	myosin light chain kinase family, member 4	1.00E-05	0.0226458	-1.11E+01
238875	Gapt	Grb2-binding adaptor, transmembrane	1.00E-05	0.0663051	-1.27E+01
238896	Cdc20b	cell division cycle 20B	1.00E-05	0.0789135	-1.29E+01
239038	Lrit2	leucine-rich repeat, immunoglobulin-like and transmembrane domains 2	1.00E-05	0.0448547	-1.21E+01
239337	Adamts12	a disintegrin-like and metallopeptidase (reprolysin type) with thrombospondin type 1 motif, 12	0.112083	1.00E-05	1.35E+01
239436	Slc30a8	solute carrier family 30 (zinc transporter), member 8	1.00E-05	0.0687927	-1.27E+01
239447	Colec10	collectin sub-family member 10	0.0432168	1.00E-05	1.21E+01
239591	Ttll8	tubulin tyrosine ligase-like family, member 8	0.285143	0.599113	-1.07E+00
239611	Muc19	mucin 19	1.00E-05	0.0264031	-1.14E+01
239659	C1ql4	complement component 1, q subcomponent-like 4	1.13273	0.273247	2.05E+00
239673	4732456N10Rik	RIKEN cDNA 4732456N10 gene	0.089525	0.187174	-1.06E+00
239852	Zpld1	zona pellucida like domain containing 1	0.179262	1.00E-05	1.41E+01
240215	Slc4a9	solute carrier family 4, sodium bicarbonate cotransporter, member 9	1.00E-05	0.0454041	-1.21E+01
240322	Adamts19	a disintegrin-like and metallopeptidase (reprolysin type) with thrombospondin type 1 motif, 19	0.276832	0.120935	1.19E+00
240328	F830016B08Rik	RIKEN cDNA F830016B08 gene	0.0879688	0.0324127	1.44E+00

240411	Loxhd1	lipoxygenase homology domains 1	1.00E-05	0.031216	-1.16E+01
240549	Gm4952	predicted gene 4952	0.221624	1.00E-05	1.44E+01
240595	Kcnv2	potassium channel, subfamily V, member 2	1.00E-05	0.0460598	-1.22E+01
240667	Sec31b	Sec31 homolog B (S. cerevisiae)	0.0939581	0.190981	-1.02E+00
240754	Lax1	lymphocyte transmembrane adaptor 1	0.0453207	0.0996579	-1.14E+00
240873	Tnfsf18	tumor necrosis factor (ligand) superfamily, member 18	1.00E-05	0.0556679	-1.24E+01
240879	Mettl11b	methyltransferase like 11B	0.477295	1.00E-05	1.55E+01
240916	Vsig8	V-set and immunoglobulin domain containing 8	0.684375	0.109958	2.64E+00
241035	Pkhd1	polycystic kidney and hepatic disease 1	0.0159538	1.00E-05	1.06E+01
241070	Gpr1	G protein-coupled receptor 1	0.276005	0.579308	-1.07E+00
241113	Prkag3	protein kinase, AMP-activated, gamma 3 non-catatlytic subunit	0.0397611	0.168403	-2.08E+00
241128	Fam124b	family with sequence similarity 124, member B	1.00E-05	0.0435913	-1.21E+01
241431	Xirp2	xin actin-binding repeat containing 2	0.0200508	0.00751314	1.42E+00
241452	Dhrs9	dehydrogenase/reductase (SDR family) member 9	1.00E-05	0.0849223	-1.31E+01
241877	Slc10a5	solute carrier family 10 (sodium/bile acid cotransporter family), member 5	0.0577724	1.00E-05	1.25E+01
241943	Ccdc144b	coiled-coil domain containing 144B	0.0346062	0.116528	-1.75E+00
242151	Kcna10	potassium voltage-gated channel, shaker-related subfamily, member 10	1.00E-05	0.0677698	-1.27E+01
242316	Gdf6	growth differentiation factor 6	0.0712063	0.151949	-1.09E+00
242341	Atp6v0d2	ATPase, H+ transporting, lysosomal V0 subunit D2	0.124973	1.00E-05	1.36E+01
242408	Fam221b	family with sequence similarity 221, member B	0.45831	0.209606	1.13E+00
242523	Dmrta1	doublesex and mab-3 related transcription factor like family A1	0.0841945	0.214854	-1.35E+00
242602	BC055111	cDNA sequence BC055111	1.00E-05	0.0559787	-1.25E+01

242653	Cldn19	claudin 19	0.0842463	0.428323	-2.35E+00
242700	Ifnlr1	interferon lambda receptor 1	0.200385	0.0594703	1.75E+00
242707	Lactbl1	lactamase, beta-like 1	1.00E-05	0.0952066	-1.32E+01
243078	Tecrl	trans-2,3-enoyl-CoA reductase-like	0.149177	1.00E-05	1.39E+01
243168	Hsd17b13	hydroxysteroid (17-beta) dehydrogenase 13	0.356083	1.00E-05	1.51E+01
243262	Oas1f	2'-5' oligoadenylate synthetase 1F	1.00E-05	0.07852	-1.29E+01
243537	Uroc1	urocanase domain containing 1	1.00E-05	0.0702246	-1.28E+01
243897	Ggn	gametogenetin	0.917518	0.293758	1.64E+00
243967	Ntn5	netrin 5	1.30757	2.65583	-1.02E+00
244071	Agbl1	ATP/GTP binding protein-like 1	0.0639993	1.00E-05	1.26E+01
244180	E030002003Rik	RIKEN cDNA E030002003 gene	1.00E-05	0.0545825	-1.24E+01
244202	Nlrp10	NLR family, pyrin domain containing 10	0.151604	1.00E-05	1.39E+01
244209	Cyp2r1	cytochrome P450, family 2, subfamily r, polypeptide 1	0.583356	0.161439	1.85E+00
244237	Tnfrsf26	tumor necrosis factor receptor superfamily, member 26	1.00E-05	0.0408793	-1.20E+01
244550	Podnl1	podocan-like 1	1.00E-05	0.144023	-1.38E+01
245240	9930111J21Rik2	RIKEN cDNA 9930111J21 gene 2	0.286947	0.102419	1.49E+00
245381	Sowahd	sosondowah ankyrin repeat domain family member D	1.00E-05	0.0941335	-1.32E+01
245532	Awat2	acyl-CoA wax alcohol acyltransferase 2	0.16969	1.00E-05	1.41E+01
245536	Gm614	predicted gene 614	1.00E-05	0.182301	-1.42E+01
245615	Kir3dl2	killer cell immunoglobulin-like receptor, three domains, long cytoplasmic tail, 2	0.0556873	0.184681	-1.73E+00
245827	Fat2	FAT tumor suppressor homolog 2 (Drosophila)	0.0216796	0.0451343	-1.06E+00
245945	Rbm47	RNA binding motif protein 47	0.181125	0.388455	-1.10E+00
246048	Chodl	chondrolectin	1.22677	0.583339	1.07E+00
246082	Defb15	defensin beta 15	1.00E-05	0.77548	-1.62E+01

246256	Fcgr4	Fc receptor, IgG, low affinity IV	0.303055	0.631937	-1.06E+00
246709	Rgs13	regulator of G-protein signaling 13	0.276522	0.858443	-1.63E+00
246728	Oas2	2'-5' oligoadenylate synthetase 2	0.148409	0.340762	-1.20E+00
246730	Oas1a	2'-5' oligoadenylate synthetase 1A	1.00E-05	0.0643429	-1.27E+01
246788	Trpv3	transient receptor potential cation channel, subfamily V, member 3	0.0532102	0.325816	-2.61E+00
252868	Odf4	outer dense fiber of sperm tails 4	0.379229	0.131373	1.53E+00
252973	Grhl2	grainyhead-like 2 (Drosophila)	1.00E-05	0.0459785	-1.22E+01
257926	Olfr544	olfactory receptor 544	1.00E-05	0.158312	-1.40E+01
258019	Olfr212	olfactory receptor 212	0.0898115	1.00E-05	1.31E+01
258064	Olfr316	olfactory receptor 316	0.184005	0.740784	-2.01E+00
258095	Olfr119	olfactory receptor 119	1.00E-05	0.157661	-1.39E+01
258325	Olfr110	olfactory receptor 110	0.282596	1.00E-05	1.48E+01
258352	Olfr692	olfactory receptor 692	0.324406	0.161853	1.00E+00
258380	Olfr461	olfactory receptor 461	1.00E-05	0.186674	-1.42E+01
258407	Olfr464	olfactory receptor 464	0.145556	0.598459	-2.04E+00
258463	Olfr1393	olfactory receptor 1393	0.324376	0.836764	-1.37E+00
258783	Olfr920	olfactory receptor 920	0.0618719	0.342952	-2.47E+00
258954	Olfr522	olfactory receptor 522	1.00E-05	0.196872	-1.43E+01
258963	Olfr539	olfactory receptor 539	0.365102	1.00E-05	1.52E+01
259108	Olfr550	olfactory receptor 550	1.00E-05	0.183088	-1.42E+01
259114	Olfr570	olfactory receptor 570	1.00E-05	0.182993	-1.42E+01
259161	Olfr688	olfactory receptor 688	1.00E-05	0.347542	-1.51E+01
260408	Prss45	protease, serine, 45	0.324475	0.111992	1.53E+00
263803	Pkn3	protein kinase N3	0.225043	0.586226	-1.38E+00

266614	Ly6g5b	lymphocyte antigen 6 complex, locus G5B	1.00E-05	0.168875	-1.40E+01
266620	Defb36	defensin beta 36	1.00E-05	0.452056	-1.55E+01
266645	Acmsd	amino carboxymuconate semialdehyde decarboxylase	1.00E-05	0.0513308	-1.23E+01
268379	Abca13	ATP-binding cassette, sub-family A (ABC1), member 13	0.0064269	0.0208941	-1.70E+00
268816	Mroh5	maestro heat-like repeat family member 5	0.342018	0.0579491	2.56E+00
268857	Nlrc3	NLR family, CARD domain containing 3	0.0845303	0.201287	-1.25E+00
268949	Dpcr1	diffuse panbronchiolitis critical region 1 (human)	1.00E-05	0.0432095	-1.21E+01
268973	Nlrc4	NLR family, CARD domain containing 4	0.0755671	0.0273248	1.47E+00
269053	Gpr152	G protein-coupled receptor 152	1.00E-05	0.0320044	-1.16E+01
269589	Sytl1	synaptotagmin-like 1	2.28114	1.13865	1.00E+00
269855	Ssc5d	scavenger receptor cysteine rich domain containing (5 domains)	0.526038	0.200336	1.39E+00
270711	Fam26d	family with sequence similarity 26, member D	1.00E-05	0.0344578	-1.18E+01
271424	Ip6k3	inositol hexaphosphate kinase 3	0.320233	0.704835	-1.14E+00
271639	Adcy10	adenylate cyclase 10	1.00E-05	0.0209695	-1.10E+01
271697	Cdk15	cyclin-dependent kinase 15	0.505611	0.0872333	2.54E+00
272382	Spib	Spi-B transcription factor (Spi-1/PU.1 related)	1.00E-05	0.0521136	-1.23E+01
272411	B3gnt6	UDP-GlcNAc:betaGal beta-1,3-N-acetylglucosaminyltransferase 6 (core 3 synthase)	0.144725	0.357592	-1.31E+00
276950	Slfn8	schlafen 8	0.210706	0.526654	-1.32E+00
277753	Cyp4a12a	cytochrome P450, family 4, subfamily a, polypeptide 12a	1.00E-05	0.0525865	-1.24E+01
278672	Duxbl1	double homeobox B-like 1	0.640807	1.00E-05	1.60E+01
278679	Apol7b	apolipoprotein L 7b	0.145335	1.00E-05	1.38E+01
278725	E130310I04Rik	RIKEN cDNA E130310I04 gene	1.00E-05	0.034912	-1.18E+01
279029	Gm711	predicted gene 711	0.263471	0.10981	1.26E+00

280408	Rilp	Rab interacting lysosomal protein	1.1063	0.34732	1.67E+00
280621	BC089491	cDNA sequence BC089491	0.782465	0.259982	1.59E+00
317757	Gimap5	GTPase, IMAP family member 5	0.267206	0.114184	1.23E+00
319146	Ifnz	interferon zeta	1.00E-05	0.110609	-1.34E+01
319152	Hist1h3h	histone cluster 1, H3h	1.88956	0.594275	1.67E+00
319155	Hist1h4c	histone cluster 1, H4c	1.00E-05	1.03914	-1.67E+01
319159	Hist1h4j	histone cluster 1, H4j	7.06124	14.464	-1.03E+00
319160	Hist1h4k	histone cluster 1, H4k	1.00E-05	2.00537	-1.76E+01
319168	Hist1h2ah	histone cluster 1, H2ah	1.00E-05	1.55569	-1.72E+01
319182	Hist1h2bh	histone cluster 1, H2bh	1.2719	3.20975	-1.34E+00
319184	Hist1h2bk	histone cluster 1, H2bk	2.60316	1.00E-05	1.80E+01
319189	Hist2h2bb	histone cluster 2, H2bb	1.00E-05	0.40672	-1.53E+01
319191	Hist1h2ai	histone cluster 1, H2ai	2.33762	9.76752	-2.06E+00
319192	Hist2h2aa2	histone cluster 2, H2aa2	0.0020267	3.17029	-1.06E+01
319216	4932441J04Rik	RIKEN cDNA 4932441J04 gene	0.30928	0.847565	-1.45E+00
319314	A930001C03Rik	RIKEN cDNA A930001C03 gene	1.00E-05	0.168679	-1.40E+01
319446	Dpep2	dipeptidase 2	1.00E-05	0.0753254	-1.29E+01
319482	9530053A07Rik	RIKEN cDNA 9530053A07 gene	0.0151146	0.0452458	-1.58E+00
319562	9630028B13Rik	RIKEN cDNA 9630028B13 gene	0.196384	0.0695551	1.50E+00
319581	Xkr5	X Kell blood group precursor-related family, member 5	0.0859806	0.218022	-1.34E+00
319646	D630013N20Rik	RIKEN cDNA D630013N20 gene	1.00E-05	0.131222	-1.37E+01
319682	D830026I12Rik	RIKEN cDNA D830026I12 gene	1.00E-05	0.0883379	-1.31E+01
319684	5031425F14Rik	RIKEN cDNA 5031425F14 gene	1.00E-05	0.0700709	-1.28E+01
319701	Fbxo48	F-box protein 48	0.503058	0.0556303	3.18E+00
319707	C430002N11Rik	RIKEN cDNA C430002N11 gene	1.00E-05	0.0809253	-1.30E+01

319727	A330035P11Rik	RIKEN cDNA A330035P11 gene	0.450491	0.0437973	3.36E+00
319772	C130050018Rik	RIKEN cDNA C130050018 gene	0.218286	0.071675	1.61E+00
319803	A430090L17Rik	RIKEN cDNA A430090L17 gene	1.00E-05	0.0310569	-1.16E+01
319812	9430018G01Rik	RIKEN cDNA 9430018G01 gene	0.0482619	1.00E-05	1.22E+01
319819	4932435022Rik	RIKEN cDNA 4932435022 gene	1.00E-05	0.0324838	-1.17E+01
319859	E030011005Rik	RIKEN cDNA E030011005 gene	0.188083	1.38869	-2.88E+00
319942	A530016L24Rik	RIKEN cDNA A530016L24 gene	0.36305	0.0464525	2.97E+00
319960	4930513N10Rik	RIKEN cDNA 4930513N10 gene	0.7762	0.324231	1.26E+00
319973	A630077J23Rik	RIKEN cDNA A630077J23 gene	1.00E-05	0.153221	-1.39E+01
319981	C530044C16Rik	RIKEN cDNA C530044C16 gene	0.381723	0.101891	1.91E+00
319997	A630001G21Rik	RIKEN cDNA A630001G21 gene	1.00E-05	0.17095	-1.41E+01
320019	7530420F21Rik	RIKEN cDNA 7530420F21 gene	1.00E-05	0.113189	-1.35E+01
320022	Ccdc79	coiled-coil domain containing 79	0.128898	0.324101	-1.33E+00
320054	9230116N13Rik	RIKEN cDNA 9230116N13 gene	0.10719	0.228293	-1.09E+00
320072	A830019L24Rik	RIKEN cDNA A830019L24 gene	0.0707421	0.157654	-1.16E+00
320088	C030034L19Rik	RIKEN cDNA C030034L19 gene	0.0720498	1.00E-05	1.28E+01
320132	A230108P19Rik	RIKEN cDNA A230108P19 gene	0.0850842	1.00E-05	1.31E+01
320135	BC049715	cDNA sequence BC049715	0.379882	0.103642	1.87E+00
320209	Ddx11	DEAD/H (Asp-Glu-Ala-Asp/His) box helicase 11	0.184871	0.443321	-1.26E+00
320249	D130009I18Rik	RIKEN cDNA D130009I18 gene	0.082085	1.00E-05	1.30E+01
320273	B230208H11Rik	RIKEN cDNA B230208H11 gene	0.280518	0.590358	-1.07E+00
320277	Spef2	sperm flagellar 2	0.497259	0.173018	1.52E+00
320355	Lipi	lipase, member I	1.00E-05	0.0800291	-1.30E+01
320363	D030045P18Rik	RIKEN cDNA D030045P18 gene	1.00E-05	0.178963	-1.41E+01
320407	Klri2	killer cell lectin-like receptor family I member 2	0.0259258	0.0570549	-1.14E+00

320440	9530091C08Rik	RIKEN cDNA 9530091C08 gene	1.00E-05	0.0151521	-1.06E+01
320463	F630111L10Rik	RIKEN cDNA F630111L10 gene	1.00E-05	0.0159144	-1.06E+01
320479	C330024D21Rik	RIKEN cDNA C330024D21 gene	1.00E-05	0.0996965	-1.33E+01
320581	Idi2	isopentenyl-diphosphate delta isomerase 2	0.129124	1.00E-05	1.37E+01
320614	A330033J07Rik	RIKEN cDNA A330033J07 gene	0.393093	0.184224	1.09E+00
320635	Cyb5r2	cytochrome b5 reductase 2	0.243048	0.087103	1.48E+00
320842	C230035I16Rik	RIKEN cDNA C230035I16 gene	0.261171	2.48986	-3.25E+00
320864	Krt26	keratin 26	0.0388405	0.12548	-1.69E+00
320893	6430562O15Rik	RIKEN cDNA 6430562O15 gene	0.650416	1.00E-05	1.60E+01
321014	4930473A02Rik	RIKEN cDNA 4930473A02 gene	0.607525	1.00E-05	1.59E+01
326620	Hist1h4b	histone cluster 1, H4b	1.00E-05	0.98967	-1.66E+01
326623	Tnfsf15	tumor necrosis factor (ligand) superfamily, member 15	0.0168062	0.0370707	-1.14E+00
327743	Wisp3	WNT1 inducible signaling pathway protein 3	1.00E-05	0.157808	-1.39E+01
327766	Tmem26	transmembrane protein 26	0.199864	0.0717554	1.48E+00
327799	Usp44	ubiquitin specific peptidase 44	0.0934868	1.00E-05	1.32E+01
327946	Gm12295	predicted gene 12295	0.061449	1.00E-05	1.26E+01
327957	Scimp	SLP adaptor and CSK interacting membrane protein	0.123945	0.262973	-1.09E+00
327992	Hsf5	heat shock transcription factor family member 5	0.0308144	0.260149	-3.08E+00
328258	Slc25a48	solute carrier family 25, member 48	1.00E-05	0.36194	-1.51E+01
328314	Gm5086	predicted gene 5086	0.560986	0.0997999	2.49E+00
328381	Sh2d4b	SH2 domain containing 4B	0.0839399	0.177874	-1.08E+00
328440	Npm2	nucleophosmin/nucleoplasmin 2	0.109554	0.355814	-1.70E+00
328489	A630020A06	uncharacterized A630020A06	0.0610975	0.204512	-1.74E+00
328561	Apol10b	apolipoprotein L 10B	0.0479858	0.156723	-1.71E+00
328695	Gm813	predicted gene 813	0.381708	1.00E-05	1.52E+01

328783	Mslnl	mesothelin-like	0.0992026	0.04856	1.03E+00
328906	Gm15328	predicted gene 15328	0.327775	0.72194	-1.14E+00
328967	Arhgef37	Rho guanine nucleotide exchange factor (GEF) 37	0.466058	0.20487	1.19E+00
329065	Scd4	stearoyl-coenzyme A desaturase 4	0.416327	0.203864	1.03E+00
329159	9130227L01Rik	RIKEN cDNA 9130227L01 gene	1.00E-05	0.0662134	-1.27E+01
329271	C230024C17Rik	RIKEN cDNA C230024C17 gene	1.00E-05	0.0323687	-1.17E+01
329278	Tnn	tenascin N	0.0840801	0.0348956	1.27E+00
329384	Ptrh1	peptidyl-tRNA hydrolase 1 homolog (S. cerevisiae)	0.913725	1.97236	-1.11E+00
329483	Gm14015	predicted gene 14015	1.00E-05	0.0877917	-1.31E+01
329509	1810024B03Rik	RIKEN cDNA 1810024B03 gene	0.560353	0.0989436	2.50E+00
329513	A730036I17Rik	RIKEN cDNA A730036I17 gene	0.124568	0.265377	-1.09E+00
329514	Gm14047	predicted gene 14047	0.224729	1.00E-05	1.45E+01
329554	Gm826	predicted gene 826	1.00E-05	0.235521	-1.45E+01
329562	A530013C23Rik	RIKEN cDNA A530013C23 gene	0.197704	1.00E-05	1.43E+01
329727	Dennd2c	DENN/MADD domain containing 2C	0.215118	0.0701058	1.62E+00
330086	Gm10440	predicted gene 10440	1.00E-05	0.0416502	-1.20E+01
330385	9530026P05Rik	RIKEN cDNA 9530026P05 gene	0.799854	1.00E-05	1.63E+01
330440	Gm766	predicted gene 766	0.0818609	0.175436	-1.10E+00
330721	Nek5	NIMA (never in mitosis gene a)-related expressed kinase 5	0.149403	0.366436	-1.29E+00
330921	Pate2	prostate and testis expressed 2	0.0455186	0.153429	-1.75E+00
330951	Gm16130	predicted gene 16130	1.00E-05	0.0432443	-1.21E+01
330956	Gm5122	predicted gene 5122	1.00E-05	0.077449	-1.29E+01
330958	A730043L09Rik	RIKEN cDNA A730043L09 gene	0.337457	0.113565	1.57E+00
331493	Gm5127	predicted gene 5127	0.0743466	0.161255	-1.12E+00
331531	AV320801	expressed sequence AV320801	1.00E-05	0.0322717	-1.17E+01

331537	E230019M04Rik	RIKEN cDNA E230019M04 gene	0.0717941	0.158082	-1.14E+00
332309	Grxcr2	glutaredoxin, cysteine rich 2	1.00E-05	0.267803	-1.47E+01
332427	Lyg2	lysozyme G-like 2	0.083324	1.00E-05	1.30E+01
332713	BC051628	cDNA sequence BC051628	1.00E-05	0.0952336	-1.32E+01
332937	Tfap2e	transcription factor AP-2, epsilon	1.00E-05	0.0810839	-1.30E+01
332942	Gm853	predicted gene 853	1.00E-05	0.116326	-1.35E+01
333424	A4gnt	alpha-1,4-N-acetylglucosaminyltransferase	0.0408935	0.131333	-1.68E+00
333473	Zfp3613	zinc finger protein 36, C3H type-like 3	1.00E-05	0.0655039	-1.27E+01
333564	Fndc3c1	fibronectin type III domain containing 3C1	1.00E-05	0.0223109	-1.11E+01
333588	Gm15104	predicted gene 15104	1.00E-05	0.409056	-1.53E+01
338350	Acad12	acyl-Coenzyme A dehydrogenase family, member 12	0.867756	1.76493	-1.02E+00
338403	Cndp1	carnosine dipeptidase 1 (metallopeptidase M20 family)	0.0778011	0.205204	-1.40E+00
353130	Prss33	protease, serine, 33	1.00E-05	0.104533	-1.34E+01
353188	Adam32	a disintegrin and metallopeptidase domain 32	0.0422481	0.229454	-2.44E+00
353204	Aldoart1	aldolase 1 A retrogene 1	0.168749	0.415325	-1.30E+00
353371	Oxct2b	3-oxoacid CoA transferase 2B	0.4511	0.0890591	2.34E+00
379043	Raet1e	retinoic acid early transcript 1E	1.00E-05	0.181784	-1.41E+01
380842	Stmnd1	stathmin domain containing 1	0.219678	0.07939	1.47E+00
380878	AF067063	cDNA sequence AF067063	1.00E-05	0.133646	-1.37E+01
380930	9330188P03Rik	RIKEN cDNA 9330188P03 gene	1.34282	0.515378	1.38E+00
381073	Npw	neuropeptide W	1.00E-05	0.3916	-1.53E+01
381091	H2-Eb2	histocompatibility 2, class II antigen E beta2	0.0643296	1.00E-05	1.27E+01
381101	Dnph1	2'-deoxynucleoside 5'-phosphate N-hydrolase 1	1.3508	2.73007	-1.02E+00
381175	Ccdc68	coiled-coil domain containing 68	0.0580547	0.259571	-2.16E+00
381272	A630095N17Rik	RIKEN cDNA A630095N17 gene	0.474808	1.44226	-1.60E+00

381293	Kif14	kinesin family member 14	0.059235	0.0255591	1.21E+00
381308	Mnda	myeloid cell nuclear differentiation antigen	1.00E-05	0.0598881	-1.25E+01
381319	Batf3	basic leucine zipper transcription factor, ATF-like 3	0.441389	0.216758	1.03E+00
381347	4930412O13Rik	RIKEN cDNA 4930412O13 gene	0.0285435	0.239464	-3.07E+00
381409	Cdh26	cadherin-like 26	0.0381106	0.0802174	-1.07E+00
381476	Stpg2	sperm tail PG rich repeat containing 2	1.00E-05	0.08708	-1.31E+01
381493	S100a7a	S100 calcium binding protein A7A	0.400264	1.00E-05	1.53E+01
381546	Ccdc24	coiled-coil domain containing 24	1.26929	0.537633	1.24E+00
381549	Zfp69	zinc finger protein 69	0.519987	0.182341	1.51E+00
381591	L1td1	LINE-1 type transposase domain containing 1	1.00E-05	0.0655071	-1.27E+01
381680	Nxpe5	neurexophilin and PC-esterase domain family, member 5	1.00E-05	0.0532612	-1.24E+01
381693	Wdr95	WD40 repeat domain 95	0.186869	0.048115	1.96E+00
381714	Gm9758	predicted gene 9758	0.23928	0.088592	1.43E+00
381792	2310040G24Rik	RIKEN cDNA 2310040G24 gene	0.720772	1.47879	-1.04E+00
381798	4930590J08Rik	RIKEN cDNA 4930590J08 gene	0.105554	1.00E-05	1.34E+01
381827	1700073E17Rik	ribosomal protein L7 pseudogene	0.0229032	0.0989249	-2.11E+00
381845	2310014L17Rik	RIKEN cDNA 2310014L17 gene	0.193485	0.479317	-1.31E+00
381853	Gipr	gastric inhibitory polypeptide receptor	0.355609	0.965135	-1.44E+00
382045	Gpr114	G protein-coupled receptor 114	1.00E-05	0.051137	-1.23E+01
382064	Gm1110	predicted gene 1110	0.165791	0.0447532	1.89E+00
382097	Gm1123	predicted gene 1123	1.00E-05	0.177077	-1.41E+01
382139	Gm1715	predicted gene 1715	1.00E-05	0.279382	-1.48E+01
382384	Odf3l2	outer dense fiber of sperm tails 3-like 2	0.116065	0.336832	-1.54E+00
382864	Colq	collagen-like tail subunit (single strand of homotrimer) of asymmetric acetylcholinesterase	0.489995	0.228753	1.10E+00

382913	Neil2	nei like 2 (E. coli)	0.862772	0.420397	1.04E+00
383258	Vmn2r118	vomeronasal 2, receptor 118	1.00E-05	0.0490272	-1.23E+01
384198	Fam47e	family with sequence similarity 47, member E	0.490222	0.206262	1.25E+00
384619	Ccdc155	coiled-coil domain containing 155	1.06652	0.487222	1.13E+00
384724	Cyp2t4	cytochrome P450, family 2, subfamily t, polypeptide 4	1.00E-05	0.0964372	-1.32E+01
385668	Lca5l	Leber congenital amaurosis 5-like	0.168758	0.390321	-1.21E+00
399570	A730085A09Rik	RIKEN cDNA A730085A09 gene	0.146753	0.0523301	1.49E+00
402757	9830166K06Rik	RIKEN cDNA 9830166K06 gene	0.198288	0.0825256	1.26E+00
403183	Mettl21e	methyltransferase like 21E	1.00E-05	0.0348239	-1.18E+01
403202	A430093F15Rik	RIKEN cDNA A430093F15 gene	0.233495	0.0615729	1.92E+00
403395	Clec3a	C-type lectin domain family 3, member a	1.00E-05	0.0393177	-1.19E+01
404337	Olfr1383	olfactory receptor 1383	1.00E-05	0.319136	-1.50E+01
404710	Iqgap3	IQ motif containing GTPase activating protein 3	0.0624769	0.168644	-1.43E+00
406219	Krt83	keratin 83	0.215197	1.00E-05	1.44E+01
407789	BC048644	cDNA sequence BC048644	0.10456	1.00E-05	1.34E+01
407790	Ndufa4l2	NADH dehydrogenase (ubiquinone) 1 alpha subcomplex, 4-like 2	4.15393	1.79749	1.21E+00
408254	A630019I02Rik	RIKEN cDNA A630019I02 gene	0.228352	0.048561	2.23E+00
432479	4930404N11Rik	RIKEN cDNA 4930404N11 gene	3.5983	9.15151	-1.35E+00
432488	Gm17745	predicted gene, 17745	1.00E-05	0.153451	-1.39E+01
432555	Gm5431	predicted gene 5431	0.23542	0.480257	-1.03E+00
432677	Vrtn	vertebrae development associated	0.133703	1.00E-05	1.37E+01
432770	Rslcan18	regulator of sex-limitation candidate 18	0.200821	0.470692	-1.23E+00
432839	Gprin2	G protein regulated inducer of neurite outgrowth 2	1.31651	0.540117	1.29E+00
432870	Gm5464	predicted gene 5464	1.00E-05	0.184566	-1.42E+01
433091	Pnpla1	patatin-like phospholipase domain containing 1	0.244532	0.0754521	1.70E+00

433171	Gm10549	predicted gene 10549	1.00E-05	0.0896268	-1.31E+01
433292	Nms	neuromedin S	1.00E-05	0.297834	-1.49E+01
433315	Gm13749	predicted gene 13749	0.185033	0.385835	-1.06E+00
433374	Gm20743	predicted gene, 20743	1.04128	0.468652	1.15E+00
433470	AA467197	expressed sequence AA467197	0.525487	1.00E-05	1.57E+01
433632	Gm5544	predicted gene 5544	1.00E-05	0.136837	-1.37E+01
433637	Gm5547	predicted gene 5547	1.00E-05	0.406396	-1.53E+01
433638	I830077J02Rik	RIKEN cDNA I830077J02 gene	0.561622	0.236598	1.25E+00
433698	Gm12429	predicted gene 12429	0.0801799	0.0279818	1.52E+00
433791	Gm13251	predicted gene 13251	0.227799	0.483101	-1.08E+00
433855	AI506816	expressed sequence AI506816	0.426276	0.047619	3.16E+00
433944	Gm16065	predicted gene 16065	1.00E-05	0.0996504	-1.33E+01
434203	Slc28a1	solute carrier family 28 (sodium-coupled nucleoside transporter), member 1	0.106602	1.00E-05	1.34E+01
434218	Trim34b	tripartite motif-containing 34B	0.18511	1.00E-05	1.42E+01
434234	2610020H08Rik	RIKEN cDNA 2610020H08 gene	0.386394	0.18139	1.09E+00
434341	Nlrc5	NLR family, CARD domain containing 5	0.125426	0.0484322	1.37E+00
434423	Dppa5a	developmental pluripotency associated 5A	1.00E-05	0.221223	-1.44E+01
434689	Gm10220	predicted gene 10220	0.0666105	1.00E-05	1.27E+01
434756	Akap14	A kinase (PRKA) anchor protein 14	0.0637308	0.404358	-2.67E+00
434768	Rhox8	reproductive homeobox 8	1.00E-05	0.0661601	-1.27E+01
434858	Gm5643	heterogeneous nuclear ribonucleoprotein A1 pseudogene	7.15396	1.69055	2.08E+00
435391	Dupd1	dual specificity phosphatase and pro isomerase domain containing 1	0.412584	1.00E-05	1.53E+01
435766	Tnni3k	TNNI3 interacting kinase	0.192109	0.0399995	2.26E+00

435811	Ldlrad2	low density lipoprotein receptor A domain containing 2	0.0644948	0.136237	-1.08E+00
442827	Rab44	RAB44, member RAS oncogene family	0.0523501	1.00E-05	1.24E+01
442829	Ccin	calicin	1.00E-05	0.0675984	-1.27E+01
497071	Rnase13	ribonuclease, RNase A family, 13 (non-active)	1.00E-05	0.265525	-1.47E+01
544791	Myh13	myosin, heavy polypeptide 13, skeletal muscle	0.0294361	0.0634317	-1.11E+00
544881	BB287469	expressed sequence BB287469	7.29E-07	0.0627282	-1.64E+01
545055	Cma2	chymase 2, mast cell	1.00E-05	0.137847	-1.38E+01
545123	Cyp2d11	cytochrome P450, family 2, subfamily d, polypeptide 11	1.00E-05	0.0887332	-1.31E+01
545136	Fam186b	family with sequence similarity 186, member B	0.154409	0.388222	-1.33E+00
545253	Gm5820	predicted gene 5820	0.631212	0.195916	1.69E+00
545260	Arsi	arylsulfatase i	0.251518	0.0810705	1.63E+00
545291	Hpse2	heparanase 2	1.00E-05	0.0606675	-1.26E+01
545562	Amy2b	amylase 2b	0.360699	0.0793528	2.18E+00
545646	Gm13290	predicted gene 13290	1.00E-05	0.164162	-1.40E+01
545652	Gm13275	predicted gene 13275	1.00E-05	0.102323	-1.33E+01
545824	Zar1l	zygote arrest 1-like	0.148938	0.307217	-1.04E+00
545861	Gm5878	predicted gene 5878	0.0509419	1.00E-05	1.23E+01
545893	Mansc4	MANSC domain containing 4	0.587174	0.238929	1.30E+00
546519	Tmem235	transmembrane protein 235	1.00E-05	0.0772723	-1.29E+01
546886	Ccdc42b	coiled-coil domain containing 42B	0.32579	0.80648	-1.31E+00
547109	Trim43a	tripartite motif-containing 43A	0.547779	0.24266	1.17E+00
554292	AB099516	cDNA sequence AB099516	1.00E-05	0.0984876	-1.33E+01
574428	Zmynd15	zinc finger, MYND-type containing 15	0.0919269	0.231397	-1.33E+00
574519	Vax2os	Vax2 opposite strand transcript	1.00E-05	0.121707	-1.36E+01
606735	A330069E16Rik	RIKEN cDNA A330069E16 gene	0.710163	0.303923	1.22E+00

606736	C920009B18Rik	RIKEN cDNA C920009B18 gene	1.00E-05	0.0292228	-1.15E+01
619301	Tmem253	transmembrane protein 253	0.123349	0.247931	-1.01E+00
619321	9530052E02Rik	RIKEN cDNA 9530052E02 gene	1.00E-05	0.667287	-1.60E+01
619327	G630093K05Rik	RIKEN cDNA G630093K05 gene	0.0301713	0.0977094	-1.70E+00
619441	BC096441	cDNA sequence BC096441	1.42009	0.575289	1.30E+00
619597	Gm6086	predicted gene 6086	1.00E-05	0.0272727	-1.14E+01
619665	Klf14	Kruppel-like factor 14	0.242272	0.100446	1.27E+00
620078	C130026I21Rik	RIKEN cDNA C130026I21 gene	0.0520438	0.172673	-1.73E+00
620079	Gm17751	predicted gene, 17751	0.184356	0.418658	-1.18E+00
620631	Ttc30a2	tetratricopeptide repeat domain 30A2	0.054555	0.289779	-2.41E+00
620779	Gm12695	predicted gene 12695	0.0608544	0.132034	-1.12E+00
620913	Gm12185	predicted gene 12185	0.0554791	0.122658	-1.14E+00
620966	Gm6194	transmembrane protein 189 pseudogene	1.00E-05	0.0850556	-1.31E+01
621156	Apoo-ps	apolipoprotein O, pseudogene	5.48415	0.00306856	1.08E+01
621239	Nhlrc4	NHL repeat containing 4	0.479424	0.12271	1.97E+00
621893	Hist2h2ab	histone cluster 2, H2ab	1.00E-05	0.715262	-1.61E+01
622408	Mcidas	multiciliate differentiation and DNA synthesis associated cell cycle protein	0.280862	0.114095	1.30E+00
622552	Gm20751	predicted gene, 20751	0.0974885	0.206727	-1.08E+00
622554	1700123I01Rik	RIKEN cDNA 1700123I01 gene	1.00E-05	0.0817203	-1.30E+01
622665	Ccdc17	coiled-coil domain containing 17	0.293233	0.785035	-1.42E+00
622976	Gm6377	predicted gene 6377	1.00E-05	0.0432716	-1.21E+01
623131	Prr19	proline rich 19	0.302187	0.102321	1.56E+00
623273	Alms1-ps2	Alstrom syndrome 1, pseudogene 2	1.00E-05	0.0983787	-1.33E+01
623534	Nme9	NME/NM23 family member 9	0.151856	0.320583	-1.08E+00

624224	Clrn2	clarin 2	1.00E-05	0.312686	-1.49E+01
625109	Vmn2r86	vomeronasal 2, receptor 86	1.00E-05	0.0452801	-1.21E+01
625175	1700028E10Rik	RIKEN cDNA 1700028E10 gene	1.00E-05	0.150268	-1.39E+01
625253	Gm6568	DnaJ (Hsp40) homolog, subfamily B, member 9 pseudogene	0.726086	1.00E-05	1.61E+01
625286	Tmem236	transmembrane protein 236	0.0615885	0.134041	-1.12E+00
625464	Gm6588	predicted gene 6588	1.00E-05	0.0465946	-1.22E+01
625850	Gm11166	predicted gene 11166	1.00E-05	0.0702045	-1.28E+01
626009	Gm6644	Akr1b3 pseudogene	13.6297	0.100805	7.08E+00
626055	Gm15645	predicted gene 15645	0.0793543	0.166307	-1.07E+00
626359	Wdr93	WD repeat domain 93	0.192287	0.464777	-1.27E+00
626415	4930467E23Rik	RIKEN cDNA 4930467E23 gene	0.384314	0.171159	1.17E+00
626596	Rgs22	regulator of G-protein signalling 22	0.336002	0.685776	-1.03E+00
627081	Xlr5b	X-linked lymphocyte-regulated 5B	1.00E-05	0.088971	-1.31E+01
627962	Gm6815	zinc finger, BED domain containing 4 pseudogene	1.00E-05	0.0317282	-1.16E+01
628705	Phf11c	PHD finger protein 11C	0.13011	1.00E-05	1.37E+01
628900	Serpina3i	serine (or cysteine) peptidase inhibitor, clade A, member 3I	1.00E-05	0.104176	-1.33E+01
629524	Olfr286	olfactory receptor 286	1.00E-05	0.104378	-1.33E+01
632687	10-Mar	membrane-associated ring finger (C3HC4) 10	0.376316	0.980241	-1.38E+00
633285	Rbm46	RNA binding motif protein 46	0.228378	0.0317713	2.85E+00
633395	Gm10548	ribosomal protein L29 pseudogene	0.372021	1.04576	-1.49E+00
633947	Gm6225	predicted gene 6225	0.149449	1.00E-05	1.39E+01
636104	Gm7173	predicted gene 7173	0.276809	0.115532	1.26E+00
639658	Gm13807	predicted gene 13807	1.00E-05	0.106054	-1.34E+01
640543	Tgm7	transglutaminase 7	0.0571196	0.117043	-1.03E+00
653030	Gm11648	predicted gene 11648	1.00E-05	0.129729	-1.37E+01

654362	Dear1	dual endothelin 1/angiotensin II receptor 1	1.0467	2.90275	-1.47E+00
654462	Kncn	kinocilin	1.00E-05	0.143012	-1.38E+01
654796	9530036O11Rik	RIKEN cDNA 9530036O11Rik	0.0359013	0.193919	-2.43E+00
654800	A730085K08Rik	RIKEN cDNA A730085K08 gene	0.557463	1.00E-05	1.58E+01
654802	1700105P06Rik	RIKEN cDNA 1700105P06 gene	1.00E-05	0.370769	-1.52E+01
654821	Gcnt7	glucosaminyl (N-acetyl) transferase family member 7	0.157656	1.00E-05	1.39E+01
664799	Ctcfl	CCCTC-binding factor (zinc finger protein)-like	0.0354607	0.147386	-2.06E+00
664903	Rps15a-ps4	ribosomal protein S15A, pseudogene 4	0.54664	0.193619	1.50E+00
665037	Gm7457	predicted gene 7457	1.00E-05	0.0480649	-1.22E+01
665268	1600029O15Rik	ribosomal protein L17 pseudogene	0.501807	1.06871	-1.09E+00
665306	3930402G23Rik	RIKEN cDNA 3930402G23 gene	1.00E-05	0.0434555	-1.21E+01
665378	Gm7609	predicted pseudogene 7609	0.266101	0.639443	-1.26E+00
665562	Rpl31-ps12	ribosomal protein L31, pseudogene 1 2	0.348624	2.51197	-2.85E+00
665622	Hist1h2br	histone cluster 1 H2br	0.193822	0.067444	1.52E+00
666040	Gm7903	predicted gene 7903	1.00E-05	0.060924	-1.26E+01
666085	Vmn2r54	vomeronasal 2, receptor 54	1.00E-05	0.0527638	-1.24E+01
666168	Cyp4a31	cytochrome P450, family 4, subfamily a, polypeptide 31	0.0989264	1.00E-05	1.33E+01
666244	Tmsb15b1	thymosin beta 15b1	1.55642	0.716982	1.12E+00
666279	Dspp	dentin sialophosphoprotein	1.00E-05	0.0220493	-1.11E+01
666348	Apol7e	apolipoprotein L 7e	1.00E-05	0.0499909	-1.23E+01
666737	4632427E13Rik	RIKEN cDNA 4632427E13 gene	1.01476	0.252658	2.01E+00
667055	Gm9992	predicted gene 9992	0.180135	0.0893102	1.01E+00
667281	H60b	histocompatibility 60b	0.221765	0.481422	-1.12E+00
667410	Gm8615	glucosamine-6-phosphate deaminase 1 pseudogene	0.201652	0.0703348	1.52E+00
667705	Gm8773	predicted gene 8773	0.109265	0.347236	-1.67E+00

667803	C920025E04Rik	RIKEN cDNA C920025E04 gene	0.388342	0.805211	-1.05E+00
668108	Gm8979	very large inducible GTPase 1 pseudogene	0.0253025	0.0577171	-1.19E+00
668128	Gm8989	very large inducible GTPase 1 pseudogene	1.00E-05	0.050343	-1.23E+01
668178	Mettl7a3	methyltransferase like 7A3	1.00E-05	0.292936	-1.48E+01
668224	Gm9054	predicted gene 9054	1.00E-05	0.225037	-1.45E+01
668257	Dgat2l6	diacylglycerol O-acyltransferase 2-like 6	0.357497	0.157747	1.18E+00
671232	Topaz1	testis and ovary specific PAZ domain containing 1	1.00E-05	0.0202976	-1.10E+01
677044	Gm10653	ribsomal protein S2 pseudogene	0.576136	0.117506	2.29E+00
768252	Foxl2os	forkhead box L2 opposite strand transcript	1.00E-05	0.139319	-1.38E+01
790912	Gm11202	predicted gene 11202	1.00E-05	0.0536406	-1.24E+01
791383	Gm9962	predicted gene 9962	1.27006	2.5499	-1.01E+00
791423	Gm11190	predicted gene 11190	0.0339982	0.0710856	-1.06E+00
1E+08	Wfdc17	WAP four-disulfide core domain 17	3.57695	1.63011	1.13E+00
1E+08	9430060I03Rik	RIKEN cDNA 9430060I03 gene	0.194628	0.394136	-1.02E+00
1E+08	Gm12359	predicted gene 12359	0.666301	0.113336	2.56E+00
1E+08	Rnaset2a	ribonuclease T2A	0.0016739	0.394685	-7.88E+00
1E+08	F630028O10Rik	RIKEN cDNA F630028O10 gene	1.00E-05	0.217984	-1.44E+01
1E+08	Gm10536	predicted gene 10536	0.045663	1.00E-05	1.22E+01
1E+08	Spink13	serine peptidase inhibitor, Kazal type 13	1.00E-05	0.203836	-1.43E+01
1E+08	Gm12522	predicted gene 12522	0.557199	0.230797	1.27E+00
1E+08	Gm15441	predicted gene 15441	1.00E-05	0.0897836	-1.31E+01
1E+08	Gm11837	predicted gene 11837	1.78111	0.824554	1.11E+00
1E+08	Gm10635	predicted gene 10635	0.0292907	0.0974766	-1.73E+00
1E+08	Gm11529	predicted gene 11529	1.00E-05	0.0604938	-1.26E+01
1E+08	Gm10474	predicted gene 10474	1.00E-05	0.0767349	-1.29E+01

1E+08	Gm10789	predicted gene 10789	0.110058	0.251822	-1.19E+00
1E+08	Gm10494	predicted gene 10494	0.0452322	0.0971337	-1.10E+00
1E+08	Ceacam-ps1	carcinoembryonic antigen-related cell adhesion molecule pseudogene 1	1.00E-05	0.207775	-1.43E+01
1E+08	Mup9	major urinary protein 9	1.00E-05	0.145723	-1.38E+01
1E+08	Gm10731	FUN14 domain containing 2 pseudogene	1.00E-05	0.0737112	-1.28E+01
1E+08	Gm2109	predicted gene 2109	0.180397	1.00E-05	1.41E+01
1E+08	Plac9b	placenta specific 9b	4.67584	0.531634	3.14E+00
1E+08	Duxbl3	double homeobox B-like 3	4.40E-05	0.404568	-1.32E+01
1E+08	Gm15706	predicted gene 15706	4.12717	8.58961	-1.06E+00
1E+08	Atp6v0c-ps2	ATPase, H+ transporting, lysosomal V0 subunit C, pseudogene 2	96.8379	0.0149242	1.27E+01
1E+08	D5Ertd605e	DNA segment, Chr 5, ERATO Doi 605, expressed	0.35849	1.00E-05	1.51E+01
1E+08	Gm581	predicted gene 581	0.0692375	0.139758	-1.01E+00
1E+08	Gm2762	predicted gene 2762	0.0888306	0.189403	-1.09E+00
1E+08	Dynlt1f	dynein light chain Tctex-type 1F	1.00469	2.56173	-1.35E+00
1E+08	Dynlt1c	dynein light chain Tctex-type 1C	2.50744	0.845988	1.57E+00
1E+08	Gm12409	predicted gene 12409	1.00E-05	0.231353	-1.45E+01
1E+08	Gm11627	predicted gene 11627	4.2488	1.16003	1.87E+00
1E+08	Gm3086	RuvB-like protein 1 pseudogene	0.192932	0.404741	-1.07E+00
1E+08	Hist1h4m	histone cluster 1, H4m	1.00E-05	1.24423	-1.69E+01
1E+08	D4Ertd617e	DNA segment, Chr 4, ERATO Doi 617, expressed	0.376706	1.00E-05	1.52E+01
1E+08	Gm11985	predicted gene 11985	1.00E-05	0.131048	-1.37E+01
1E+08	Gm13247	predicted gene 13247	0.092242	0.0334412	1.46E+00
1E+08	Ly6c2	lymphocyte antigen 6 complex, locus C2	0.198367	1.02253	-2.37E+00

1E+08	Gm3417	predicted gene 3417	0.914669	0.327998	1.48E+00
1E+08	Mup7	major urinary protein 7	1.00E-05	0.117738	-1.35E+01
1E+08	Oaz1-ps	ornithine decarboxylase antizyme 1, pseudogene	21.8618	49.2897	-1.17E+00
1E+08	Gm15421	ribosomal protein L22 like 1 pseudogene	0.980561	0.358378	1.45E+00
1E+08	Gm3716	predicted gene 3716	1.00E-05	0.0400901	-1.20E+01
1E+08	Gm3833	meiotic nuclear divisions 1 homolog pseudogene	0.134865	0.0378632	1.83E+00
1E+08	Gm16523	mitochondrial ribosomal protein L40 pseudogene	1.00E-05	0.0494607	-1.23E+01
1E+08	Gm3985	predicted gene 3985	0.253573	0.648888	-1.36E+00
1E+08	C920021L13Rik	RIKEN cDNA C920021L13 gene	0.554469	1.00E-05	1.58E+01
1E+08	4632428C04Rik	RIKEN cDNA 4632428C04 gene	0.501587	1.10231	-1.14E+00
1E+08	Gm10012	cytochrome c oxidase, subunit VIIc pseudogene	1.00E-05	1.23688	-1.69E+01
1E+08	Gm4349	SET domain, bifurcated 1 pseudogene	0.343319	0.743381	-1.11E+00
1E+08	Ankrd66	ankyrin repeat domain 66	1.00E-05	0.178287	-1.41E+01
1E+08	AI662270	expressed sequence AI662270	0.0850188	0.369485	-2.12E+00
1E+08	A630075F10Rik	RIKEN cDNA A630075F10 gene	0.061815	0.195632	-1.66E+00
1E+08	Gm16894	predicted gene, 16894	1.3161	0.46726	1.49E+00
1E+08	Pet117	PET117 homolog (S. cerevisiae)	10.3305	5.00931	1.04E+00
1E+08	AI847159	expressed sequence AI847159	0.126739	0.26916	-1.09E+00
1E+08	Gm14873	predicted gene 14873	0.867605	0.290473	1.58E+00
1E+08	Gm10364	glyceraldehyde-3-phosphate dehydrogenase pseudogene	1.00E-05	0.0921031	-1.32E+01
1E+08	Gm11110	predicted gene 11110	0.237123	0.760015	-1.68E+00
1E+08	Gm10941	predicted gene 10941	1.00E-05	0.956549	-1.65E+01
1E+08	EU599041	expressed sequence EU599041	1.00E-05	0.0754526	-1.29E+01
1E+08	Ttll2	tubulin tyrosine ligase-like family, member 2	0.135227	0.28344	-1.07E+00
1E+08	Snora81	small nucleolar RNA, H/ACA box 81	1.00E-05	9.14404	-1.98E+01

1E+08	Gm13718	predicted gene 13718	1.00E-05	0.0316618	-1.16E+01
1E+08	Gm11944	predicted gene 11944	1.00E-05	0.154477	-1.39E+01
1E+08	Gm17455	predicted gene, 17455	0.425956	0.206589	1.04E+00
1E+08	Gm17821	DDB1 and CUL4 associated factor 5 pseudogene	0.0908943	0.1944	-1.10E+00
1E+08	Snora28	small nucleolar RNA, H/ACA box 28	1.00E-05	197.964	-2.42E+01
1E+08	Gm17384	predicted gene, 17384	0.158478	1.00E-05	1.40E+01
1E+08	Nup62-il4i1	Nup62-Il4i1 protein	1.00E-05	0.389632	-1.52E+01
1E+08	Nlrp5-ps	NLR family, pyrin domain containing 5, pseudogene	0.052282	0.167011	-1.68E+00
1.01E+08	4930469G21Rik	RIKEN cDNA 4930469G21 gene	1.00E-05	0.137784	-1.38E+01
1.01E+08	AW046200	expressed sequence AW046200	0.575163	0.175865	1.71E+00
1.01E+08	Gm13483	predicted gene 13483	0.103748	0.528457	-2.35E+00
1.01E+08	C130060C02Rik	RIKEN cDNA C130060C02 gene	0.520483	0.221082	1.24E+00
1.01E+08	Gm13826	predicted gene 13826	0.755161	1.8413	-1.29E+00
1.01E+08	Gm19434	predicted gene, 19434	1.00E-05	0.0447237	-1.21E+01
1.01E+08	Gm16998	predicted gene, 16998	0.107532	0.681048	-2.66E+00
1.01E+08	Gm16796	predicted gene, 16796	0.112174	0.0390568	1.52E+00
1.01E+08	AV051173	expressed sequence AV051173	1.00E-05	0.548005	-1.57E+01
1.01E+08	BC037704	cDNA sequence BC037704	0.311191	1.11883	-1.85E+00
1.01E+08	Gm10814	predicted gene 10814	0.211049	0.432716	-1.04E+00
1.01E+08	Gm16551	predicted gene 16551	0.0750798	1.00E-05	1.29E+01
1.01E+08	Gm19557	predicted gene, 19557	0.0432923	0.0894753	-1.05E+00
1.01E+08	Gm15471	predicted gene 15471	1.00E-05	0.245301	-1.46E+01
1.01E+08	Gm19589	predicted gene, 19589	0.126683	0.0441814	1.52E+00
1.01E+08	Gm15408	predicted gene 15408	0.0822459	0.349348	-2.09E+00
1.01E+08	Gm10390	predicted gene 10390	0.0329364	0.0983621	-1.58E+00

1.01E+08	Gm14023	predicted gene 14023	0.0697038	0.305424	-2.13E+00
1.01E+08	A930016022Rik	RIKEN cDNA A930016022 gene	0.426486	0.166876	1.35E+00
1.01E+08	LOC100503496	uncharacterized LOC100503496	0.0694826	0.150544	-1.12E+00
1.01E+08	Gm15941	predicted gene 15941	0.137242	1.00E-05	1.37E+01
1.01E+08	Nuggc	nuclear GTPase, germinal center associated	1.00E-05	0.038975	-1.19E+01
1.01E+08	Gm9926	predicted gene 9926	1.00E-05	0.0254096	-1.13E+01
1.01E+08	Zfp534	zinc finger protein 534	1.00E-05	0.0450775	-1.21E+01
1.01E+08	A330076C08Rik	RIKEN cDNA A330076C08 gene	0.533076	0.213139	1.32E+00
1.01E+08	Gm5084	predicted gene 5084	1.00E-05	0.0299814	-1.15E+01
1.01E+08	Gm16701	predicted gene, 16701	0.630287	1.49974	-1.25E+00
1.01E+08	Gm6040	predicted gene 6040	1.00E-05	0.15864	-1.40E+01
1.01E+08	Gm15787	predicted gene 15787	0.17464	0.378458	-1.12E+00
1.01E+08	Gm16157	predicted gene 16157	1.00E-05	0.110732	-1.34E+01
1.01E+08	LOC100504039	uncharacterized LOC100504039	1.00E-05	0.190016	-1.42E+01
1.01E+08	Gm15915	predicted gene 15915	1.00E-05	0.155954	-1.39E+01
1.01E+08	Gm16062	predicted gene 16062	0.925647	0.247017	1.91E+00
1.01E+08	Gm6537	predicted gene 6537	2.07342	1.00092	1.05E+00
1.01E+08	Gm20187	predicted gene, 20187	0.0611691	0.197195	-1.69E+00
1.01E+08	Gm15880	predicted gene 15880	0.11404	0.237956	-1.06E+00
1.01E+08	2900076A07Rik	RIKEN cDNA 2900076A07 gene	0.933059	2.09924	-1.17E+00
1.01E+08	Smlr1	small leucine-rich protein 1	1.00E-05	0.22557	-1.45E+01
1.01E+08	Gm20257	caspase 8 pseudogene	1.79139	0.655464	1.45E+00
1.01E+08	3425401B19Rik	RIKEN cDNA 3425401B19 gene	0.0189523	0.118	-2.64E+00
1.01E+08	Gm15910	predicted gene 15910	0.0397319	0.129685	-1.71E+00
1.01E+08	Gm11127	predicted gene 11127	0.0840422	0.43569	-2.37E+00

1.01E+08	C230037L18Rik	RIKEN cDNA C230037L18 gene	0.80205	2.007	-1.32E+00
1.01E+08	Raver1-fdx1l	Raver1-Fdx1l readthrough	0.948636	1.00E-05	1.65E+01
1.01E+08	Gm21950	predicted gene, 21950	1.00E-05	0.0599357	-1.25E+01
1.01E+08	Gm21671	predicted gene, 21671	0.221503	0.485551	-1.13E+00
1.01E+08	LOC101243624	uncharacterized LOC101243624	0.214133	0.0756653	1.50E+00
252837	Ackr4	atypical chemokine receptor 4	0.37981	0.171483	1.15E+00
-	Cutal	-	0.284834	1.00E-05	1.48E+01
69926	Dnah17	dynein, axonemal, heavy chain 17	0.0143579	0.0368983	-1.36E+00
1.01E+08	Evi2a-evi2b	Evi2a-Evi2b readthrough	0.265941	1.00E-05	1.47E+01
1.01E+08	Hbb-bs	hemoglobin, beta adult s chain	35.1749	0.0645795	9.09E+00
111975	Igf2os	insulin-like growth factor 2, opposite strand	0.166223	0.0561697	1.57E+00
1.01E+08	LOC100503676	uncharacterized LOC100503676	0.299761	0.736126	-1.30E+00
-	LOC100504703	-	1.00E-05	2.19669	-1.77E+01
1.02E+08	LOC101669761	NADH dehydrogenase (ubiquinone) 1 alpha subcomplex, 7 (B14.5a), pseudogene	1.00E-05	0.440861	-1.54E+01
78906	Misp	mitotic spindle positioning	0.0476553	0.0956731	-1.01E+00
19065	Npy4r	neuropeptide Y receptor Y4	1.00E-05	0.0770367	-1.29E+01
69129	Pex11g	peroxisomal biogenesis factor 11 gamma	1.00E-05	0.388862	-1.52E+01
432995	Smim22	small integral membrane protein 22	1.00E-05	1.28414	-1.70E+01
1E+08	Tmem51os1	Tmem51 opposite strand 1	1.00E-05	0.0802783	-1.30E+01
1.01E+08	Uchl1os	Uchl1 opposite strand transcript (head to head)	1.00E-05	0.0674071	-1.27E+01
