# Supplementary material for: Histone Deacetylase Inhibitor Alleviates the Neurodegenerative Phenotypes and Histone Dysregulation in Presenilins-Deficient Mice
Source: Front Aging Neurosci. 2018 May 15;10:137. doi: 10.3389/fnagi.2018.00137 (PMC5962686; doi:10.3389/fnagi.2018.00137)
Supplement: Supplementary file 3 [file Table_3.pdf]

Histone deacetylase inhibitor alleviates the neurodegenerative phenotypes and histone dysregulation in presenilins-deficient mice

Ting Cao[1†], Xiaojuan Zhou[1†,] Xianjie Zheng[1†], Yue Cui[1], Joe Z. Tsien[2], Chunxia Li[1*], Huimin Wang[1, 3, 4*]

[†] **These authors have contributed equally to this work.**
***Correspondence: Dr. Chunxia Li, cxli@brain.ecnu.edu.cn; Dr. Huimin Wang hmwang@nbic.ecnu.edu.cn**

[1] Shanghai Key Laboratory of Brain Functional Genomics, Key Laboratory of Brain Functional Genomics, Ministry of Education, School of Psychology and Cognitive Science, East China Normal University, Shanghai, China.
[2] Brain and Behavior Discovery Institute and Department of Neurology, Medical College of Georgia at Augusta University, Augusta, USA.
[3] NYU-ECNU Institute of Brain and Cognitive Science at NYU Shanghai, Shanghai, China.
[4] Shanghai Changning-ECNU Mental Health Center, Shanghai, China.

Supplementary Table S3: GO category enrichment based on biological process for differentially expressed genes in NaB-treated cDKO mice

Term	Count	PValue	Genes ID	Fold Enrichment	FDR
GO:0006955~immune response	44	4.99E-09	12458, 57349, 26944, 18106, 15945, 56221, 21936, 60505, 545055, 15061, 243262, 20304, 16992, 20303, 22362, 240754, 244237, 246728, 231655, 20307, 16184, 16163, 22637, 23961, 17907, 381091, 619441, 240873, 22163, 21897, 326623, 20296, 83430, 14103, 56838, 20293, 16176, 21822, 56066, 16191, 14980, 17329, 15000, 16193	2.69588	9.13E-06

GO:0002376~immune system process	54	1.04E-08	434341, 12479, 14133, 21935, 18843, 16854, 20186, 233079, 109032, 246728, 16184, 20202, 20201, 22637, 23961, 14063, 100689, 12229, 53791, 218624, 16534, 17857, 12480, 14728, 75766, 18106, 69379, 52685, 240754, 231655, 16666, 27218, 214763, 58203, 232371, 240873, 667281, 217306, 17002, 210757, 27007, 21897, 12902, 83430, 268973, 17087, 269855, 77647, 230738, 56620, 26888, 57444, 244202, 14980	2.349696	1.90E-05
GO:0006954~inflammatory response	48	1.05E-07	12766, 16621, 15945, 22226, 56221, 21936, 14293, 20186, 18126, 20304, 233079, 16992, 20303, 244237, 20307, 20344, 20202, 16666, 12273, 19222, 16163, 20201, 22637, 14063, 22163, 21897, 230979, 57890, 53791, 98870, 20296, 83430, 381493, 140806, 97895, 268973, 12310, 12061, 17087, 20293, 16176, 230738, 56066, 11609, 16191, 17329, 11607, 16193	2.32541	1.91E-04
GO:0032496~response to lipopolysaccharide	30	6.63E-06	21418, 57349, 15945, 21936, 50701, 66166, 227231, 18126, 16992, 244237, 12273, 19109, 19222, 20201, 16163, 100689, 19219, 22163, 230979, 13035, 14103, 12061, 17087, 16176, 56066, 211666, 17329, 268857, 13076, 84544	2.537884	0.012119
GO:0045087~innate immune response	48	7.82E-06	20459, 18106, 434341, 12479, 69379, 14133, 19288, 13214, 18843, 16854, 20186, 109032, 246728, 231655, 20202, 16666, 214763, 246082, 27218, 20201, 22637, 23961, 58203, 14063, 232371, 12229, 100689, 266620, 27007, 21897, 12902, 53791, 83430, 12796, 545652, 74735, 319146, 545646, 268973, 17087, 269855, 17857, 12480, 26888, 56620, 244202, 57444, 246730	1.999853	0.014289
GO:0090280~positive regulation of calcium ion import	9	1.64E-05	16854, 15464, 246788, 20293, 18787, 108072, 22226, 69047, 20296	7.14233	0.029962
