# Supplementary material for: Histone Deacetylase Inhibitor Alleviates the Neurodegenerative Phenotypes and Histone Dysregulation in Presenilins-Deficient Mice
Source: Front Aging Neurosci. 2018 May 15;10:137. doi: 10.3389/fnagi.2018.00137 (PMC5962686; doi:10.3389/fnagi.2018.00137)
Supplement: Supplementary file 4 [file Table_4.pdf]

Histone deacetylase inhibitor alleviates the neurodegenerative phenotypes and histone dysregulation in presenilins-deficient mice

Ting Cao[1†], Xiaojuan Zhou[1†,] Xianjie Zheng[1†], Yue Cui[1], Joe Z. Tsien[2], Chunxia Li[1*], Huimin Wang[1, 3, 4*]

[†] **These authors have contributed equally to this work.**
***Correspondence: Dr. Chunxia Li, cxli@brain.ecnu.edu.cn; Dr. Huimin Wang hmwang@nbic.ecnu.edu.cn**

[1] Shanghai Key Laboratory of Brain Functional Genomics, Key Laboratory of Brain Functional Genomics, Ministry of Education, School of Psychology and Cognitive Science, East China Normal University, Shanghai, China.
[2] Brain and Behavior Discovery Institute and Department of Neurology, Medical College of Georgia at Augusta University, Augusta, USA.
[3] NYU-ECNU Institute of Brain and Cognitive Science at NYU Shanghai, Shanghai, China.
[4] Shanghai Changning-ECNU Mental Health Center, Shanghai, China.

Supplementary Table S4: Pathway enrichment for differentially expressed genes in NaB-treated cDKO mice

Term	Count	PValue	Genes ID	Fold Enrichment	FDR
mmu04060:Cytokine-cytokine receptor interaction	38	4.15E-08	57349, 12458, 12766, 230828, 15945, 53603, 60505, 21936, 21935, 20304, 16992, 20303, 16185, 20307, 16184, 16182, 19109, 16163, 14256, 240873, 22163, 230979, 326623, 11705, 20296, 83430, 14103, 140806, 56838, 16178, 20293, 16176, 242700, 56066, 16191, 17329, 16156, 16193	2.70291	5.40E-05

mmu04080:Neuroactive ligand-receptor interaction	41	9.17E-08	64095, 65086, 226278, 14938, 217369, 18169, 216749, 14293, 12640, 13491, 193034, 11443, 19065, 21337, 14065, 12273, 19109, 19222, 26361, 14063, 17199, 381853, 19219, 231602, 108072, 13035, 17200, 14829, 17203, 14408, 14409, 12061, 14599, 14429, 18815, 15559, 14602, 14308, 11609, 18436, 11607	2.50699	1.19E-04
