# Supplementary material for: Histone Deacetylase Inhibitor Alleviates the Neurodegenerative Phenotypes and Histone Dysregulation in Presenilins-Deficient Mice
Source: Front Aging Neurosci. 2018 May 15;10:137. doi: 10.3389/fnagi.2018.00137 (PMC5962686; doi:10.3389/fnagi.2018.00137)
Supplement: Supplementary file 5 [file Image_1.pdf]

Histone deacetylase inhibitor alleviates the neurodegenerative phenotypes and histone dysregulation in presenilins-deficient mice

Ting Cao[1†], Xiaojuan Zhou[1†,] Xianjie Zheng[1†], Yue Cui[1], Joe Z. Tsien[2], Chunxia Li[1*], Huimin Wang[1, 3, 4*]

[†] **These authors have contributed equally to this work.**
***Correspondence: Dr. Chunxia Li, cxli@brain.ecnu.edu.cn; Dr. Huimin Wang hmwang@nbic.ecnu.edu.cn**

[1] Shanghai Key Laboratory of Brain Functional Genomics, Key Laboratory of Brain Functional Genomics, Ministry of Education, School of Psychology and Cognitive Science, East China Normal University, Shanghai, China.
[2] Brain and Behavior Discovery Institute and Department of Neurology, Medical College of Georgia at Augusta University, Augusta, USA.
[3] NYU-ECNU Institute of Brain and Cognitive Science at NYU Shanghai, Shanghai, China.
[4] Shanghai Changning-ECNU Mental Health Center, Shanghai, China.

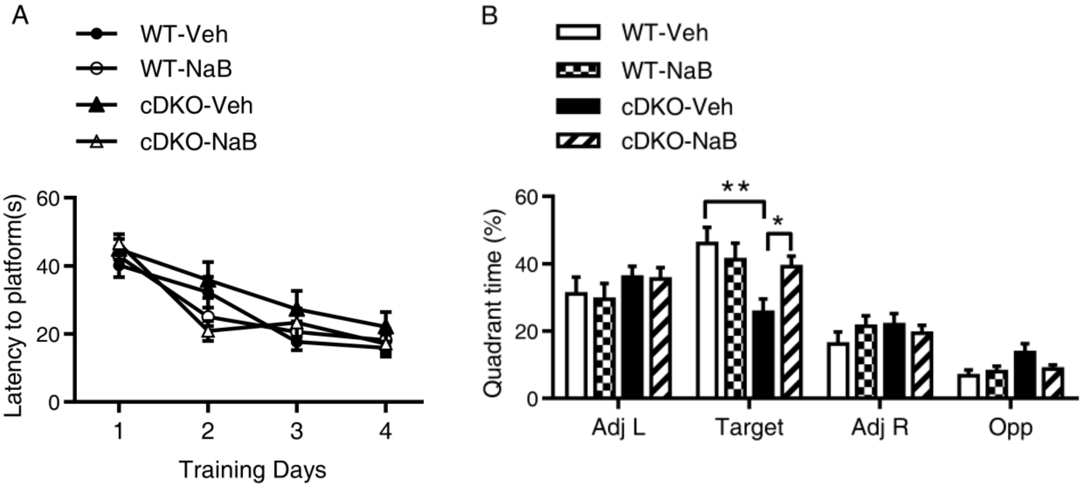

Supplementary FIGURE S1 Impaired spatial memory in cDKO mice was improved by NaB treatment. The water maze is a circular pool (120cm in diameter).The training protocol consisted of four sessions (4 trials per session per day, 5min interval intertrial interval).The movement of mice was tracked by an automated tracking system and the escape latency to the platform was recorded. Following training days 4, mice were subjected to a probe trial on day 5. During the probe test, the platform was removed and the mice were allowed to swim in the pool for 60s. The time spent in each quadrant was recorded. (A) Mean escape latency to the platform during training day 1-4 (B) During the probe trial on day 5, reduced quadrant time in cDKO mice was significantly increased by NaB treatment (Veh: WT n =10,cDKO n = 8; NaB: WT n = 10,cDKO n=12). Adi L, adjacent left; Target, target quadrant; Adj R, adjacent right; Opp, opposite quadrant. Data was expressed as mean ± SEM. Student's t test. $^*P<0.05$, $^{**}P<0.01$.
